# Management of triplet excitons transition: fine regulation of Förster and dexter energy transfer simultaneously

**DOI:** 10.1038/s41377-023-01366-1

**Published:** 2024-01-30

**Authors:** Jiaqiang Wang, Yujie Yang, Xinnan Sun, Xiaoning Li, Liyao Zhang, Zhen Li

**Affiliations:** 1 Institute of Molecular Aggregation Science, Tianjin University, Tianjin, 300072 China; 2 School of Life Sciences, Tianjin University, Tianjin, 300072 China; 3 Hubei Key Lab on Organic and Polymeric Opto-Electronic Materials, Department of Chemistry, Wuhan University, Wuhan, 430072 China; 4 Wuhan National Laboratory for Optoelectronics, Huazhong University of Science and Technology, Wuhan, 430072 China; 5 Joint School of National University of Singapore, Tianjin University, International Campus of Tianjin University, Binhai New City, Fuzhou 350207 China

**Keywords:** Optical materials and structures, Other photonics

## Abstract

Understanding and management of triplet excitons transition in the same molecule remain a great challenge. Hence, for the first time, by host engineering, manageable transitions of triplet excitons in a naphthalimide derivative **NDOH** were achieved, and monitored through the intensity ratio (I_TADF_/I_RTP_) between thermally activated delayed fluorescence (TADF) and room-temperature phosphorescence (RTP). Energy differences between lowest triplet excited states of host and guest were changed from 0.03 to 0.17 eV, and I_TADF_/I_RTP_ of **NDOH** decreased by 200 times, thus red shifting the afterglow color. It was proposed that shorter conjugation length led to larger band gaps of host materials, thus contributing to efficient Dexter and inefficient Förster energy transfer. Interestingly, no transition to singlet state and only strongest RTP with quantum yield of 13.9% could be observed, when **PBNC** with loosest stacking and largest band gap acted as host. This work provides novel insight for the management and prediction of triplet exciton transitions and the development of smart afterglow materials.

## Abstract (web summary)

Through the fine regulation of Förster and Dexter energy transfer, we managed triplet excitons, and the intensity ratio between thermally activated delayed fluorescence and room-temperature phosphorescence exhibited a great change.

## Graphical abstract

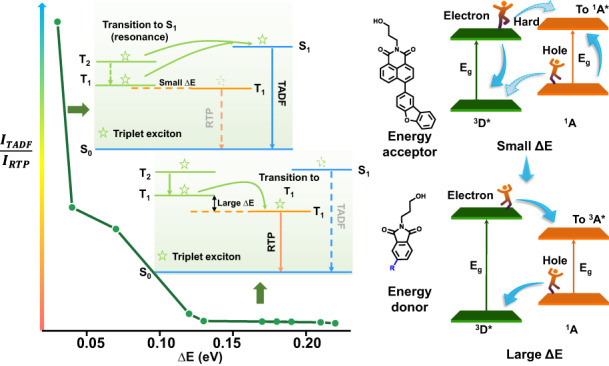

## Introduction

**Fig. 1:**
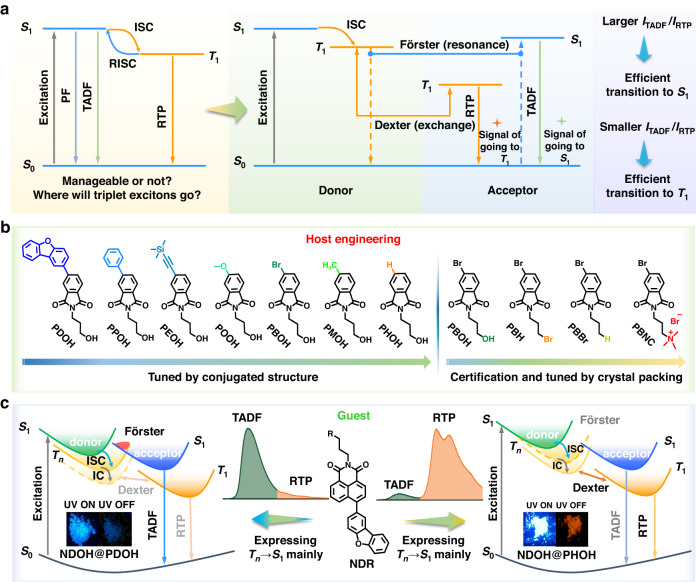
Proof of concept for management of triplet excitons. **a** The simplified Jablonski diagram and design schematic of how to manage triplet exciton transitions without changing the luminophore structures based on Dexter triplet-triplet energy transfer (TTET) and Förster triplet-singlet energy transfer (TSET). PF prompt fluorescence, TADF thermally activated delayed fluorescence, RTP room-temperature phosphorescence, ISC intersystem crossing, RISC reverse intersystem crossing. **b** Chemical structures of the host molecules in host engineering. **c** The schematic diagram of management of triplet exciton transitions to express T_n_ → S_1_ and T_n_ → T_1_ mainly and the chemical structure of guest. IC internal conversion

Organic luminescent materials have achieved a significant increase in interest for their lighting^1–3^, display^4,5^, anti-counterfeiting^6,7^ and bioimaging applications^8–10^, and the luminescent process is heavily related to excitons. Generally, excitons of the materials generated upon excitation, can be simply described as the composition of a hole and an electron. Different exciton states, including singlet and triplet states, can be defined according to the spin states of electrons. The spin-allowed relaxation of singlet excitons is more commonly observed, resulting in fluorescent emission, which have been thoroughly investigated with the singlet excitons being well managed^11–14^. However, difficulties in observation of emission involving triplet excitons due to the spin-forbidden nature of them led to only partially understanding of management of triplet excitons. So far, the emission involving triplet states mainly includes thermally activated delayed fluorescence (TADF), triplet-triplet annihilation (TTA) and phosphorescence (Fig. 1a), with great attention attracted for their wide applications^15–18^. Generally, to achieve efficient TADF, researchers try hard to change molecular structures for narrowing the energy gap between lowest singlet excited state (S_1_) and lowest triplet excited state (T_1_)^19–21^. Nevertheless, there is still a lack of a good rule of thumb for regulating the energy levels to a certain value to manage triplet excitons transition channels. As to organic room temperature phosphorescence (RTP), the management of triplet excitons is still a big challenge. So far, the good or bad management of triplet excitons could be only reflected by the performance of TADF or RTP luminogens, which, actually, is determined by many parameters^22–24^. Thus, if some other properties could directly and sensitively correlate with triplet excitons, the deep understanding of managing triplet excitons might be much convenient.

Previously, different organic luminogens with TADF or RTP properties have been successfully developed^25–37^, and few of them could exhibit both TADF and RTP with different emissive wavelength^33–37^. Considering that both TADF and RTP are related to triplet excitons, thus, perhaps, the emission intensity at different wavelength might help us to explore the management of triplet excitons. But, for an isolated system, management of triplet excitons with unchanged energy levels of emissive S_1_ and T_1_ is hardly realized, no matter it helps to study the inherent mechanism of excitons management and make the evaluation of transition more accurate. Fortunately, inspired by and based on host-guest systems with excellent RTP performance we recently developed^38–41^, the modification of non-emissive energy levels of host could be realized for the managing of triplet excitons. The different energy transfer mechanisms of Dexter exchange energy transfer and Förster resonance energy transfer (FRET) could help to control the transitions of triplet excitons to singlet or triplet states, according to different energy levels^42–48^.

Hence, we try to manage triplet excitons and study exciton transition process through host-guest systems with dual emission of TADF and RTP. Distinct from the traditional RTP host-guest systems to change the guest molecules for different afterglow colors, in this manuscript, host engineering was utilized to do the management of triplet excitons. Accordingly, a dibenzofuran substituted 1,8-naphthalimide derivative (**NDOH**) was synthesized to be the guest molecule and seven phthalimide derivatives were prepared to act as host (Fig. 1b). The management results could be evaluated based on the intensity ratios between TADF and RTP. By finely tuning the conjugated structures of phthalimide derivatives, energy differences (ΔE) between T_1_ of host and guest were tuned from 0.03 to 0.17 eV, and the resultant host-guest system exhibited different intensity ratios between TADF and RTP (I_TADF_/I_RTP_) from 20 to 0.1, which decreased by 200 times, while the afterglow color also changed from cyan to orange-red (Fig. 1c).

Moreover, it was found that larger ΔE would result in more efficient transition from T_1_ of host to that of guest. When ΔE was > 0.07 eV, I_TADF_/I_RTP_ would be < 1 and transitions to triplet state would dominate rather than to singlet state. Furthermore, the terminal group on the N-substituted alkyl chain of **PBOH** was further tuned from hydroxyl group to other groups. Interestingly, **PBNC** exhibited the loosest crystal packing and strongest spin-orbital coupling (SOC) originating from the bromide ions, resulting in no triplet-singlet transition accompanying with no TADF emission but only strongest RTP and further proving our mechanism of management of triplet excitons. Accordingly, we can not only predict the triplet-singlet and triplet-triplet transitions based on ΔE, but also develop efficient TADF or RTP materials by regulating ΔE to promote FRET or Dexter energy transfer.

## Results

### Photophysical properties

**Fig. 2:**
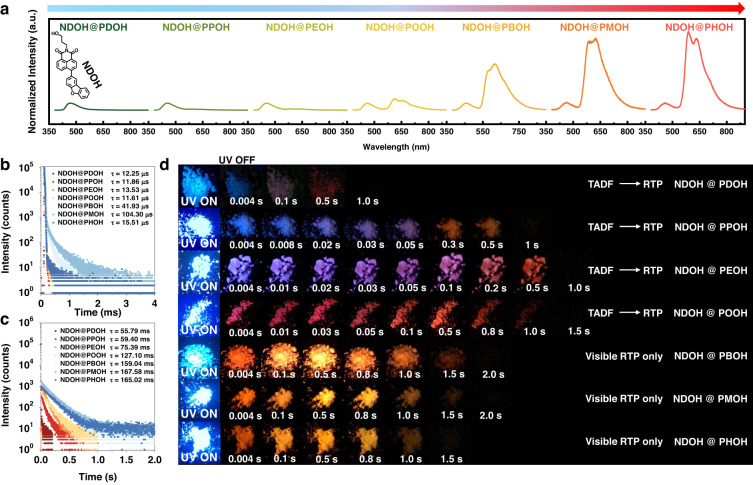
Photophysical properties of the host-guest systems with different conjugation length of host molecules. **a** Delayed spectra of **NDOH** doped in different host molecules after stopping excitation. λ_ex_ = 365 nm. Delayed time = 8 ms. The maximum emission intensities in short wavelength region are normalized. **b**, **c** Decay curves of **NDOH** doped in different host molecules monitored at maximum emission wavelength of short wavelength region (**b**) and long wavelength region (**c**). **d** Photographs of **NDOH** doped in different host molecules under UV light and after removal of UV light

1,8-Naphthalimide and phthalimide derivatives with similar structures have been reported to exhibit efficient ISC due to the promotion of n→π* transition (Figs. S1 and S2)^49–54^. However, dual emission of TADF and RTP on them or their doped materials has not been reported. Herein, ten phthalimide derivatives and four 1,8-naphthalimide derivatives were synthesized (Scheme S1). Photophysical properties of **NDOH** were first investigated (Figs. S3 and S4). **NDOH** was doped into polymethyl methacrylate (PMMA) with the mass ratio of 0.1%. Although **NDOH**@PMMA film did not exhibit RTP emission in original state, the phosphorescence emission was gradually enhanced upon UV irradiation at 365 nm, which reached maximum after about 90 s (Fig. S4). The photoactivated phosphorescence of **NDOH**@PMMA film could be quenched again after being kept in air for 3 min, but only showed a slight decrease after being placed in nitrogen for the same time. It demonstrated that the mechanism of the photoactivated process should be the consumption of oxygen upon UV irradiation, which has also been reported before by our group^18^. The large energy difference between S_1_ and T_1_ ( > 0.37 eV) of **NDOH** results in a great challenge in achieving reverse intersystem crossing (RISC). Thus, triplet excitons could only be utilized through radiative transition pathway and negligible TADF emission could be observed on **NDOH**@PMMA film. In other words, the management of triplet exciton transitions in PMMA matrix was limited. **NDOH** exhibited broad absorption range extending to 450 nm in PMMA, which had a large overlap with the PL spectra of the phthalimide derivatives with different conjugated structures (Fig. S5). Weak RTP invisible to naked eyes could also be found for the phthalimide derivatives (Figs. S6 and S7). It was supposed that Förster and Dexter energy transfer could occur between **NDOH** and the phthalimide derivatives. The spectral overlap between the phosphorescence emission of the host materials and the absorption of **NDOH** is provided in Fig. S8, demonstrating that energy transfer could occur from the triplet states of host materials to the guest molecule. Thus, to manage the triplet excitons and study the transition process, **NDOH** was then doped into different phthalimide derivatives with the molar ratio of 0.1%. Obviously, though similar fluorescence emission could be observed for the resultant host-guest systems (Fig. S9), entirely different afterglow emissions were found. To make a comprehensive comparison of TADF and RTP, the delayed spectra of the powder were investigated carefully first. As presented in Fig. 2a, all of the delayed spectra exhibited two emission peaks in short wavelength region (400–520 nm) and long wavelength region (520–850 nm). The emission in short wavelength region was normalized. For **NDOH**@**PDOH,** **NDOH**@**PPOH** and **NDOH**@**PEOH** powder, much stronger emission peak in short wavelength region could be observed. The relative intensity of the emission in long wavelength region increased as **NDOH**@**PDOH** < **NDOH**@**PPOH** < **NDOH**@**PEOH**, which was opposite to the number of the π electrons in the host molecules. When the π-electron donor was replaced by other groups with much weaker π-electron donor capacity, great enhancement of the relative intensity could be observed for the emission in long wavelength region, which increased from **NDOH**@**POOH** to **NDOH**@**PBOH,** **NDOH**@**PMOH**, and finally to **NDOH**@**PHOH**. It accorded with the decrease of electron donor capacity, since strong p-π conjugation can be found in methoxy substituted phthalimide **POOH**, and weak p-π conjugation can be found in bromine substituted phthalimide **PBOH**, while σ-π conjugation can be found in methyl substituted phthalimide **PMOH** and no conjugation should be found between hydrogen atom and the phthalimide core of **PHOH**.

Lifetimes of emission in short wavelength and long wavelength regions of all the host-guest systems were then measured. Emission in short wavelength region exhibited microsecond lifetimes (Fig. 2b) and that in long wavelength region had much longer millisecond lifetimes (Fig. 2c). It is worth mentioning that the afterglow in short wavelength region is consistent with the fluorescence of the host-guest system. Thus, the short wavelength region afterglow might be ascribed to TADF emission. As presented in Fig. S10, the emission in long wavelength region of the energy transfer systems is almost identical to the RTP emission of the photoactivated **NDOH**@PMMA film, and the phosphorescence emission of **NDOH** solution in THF at 77 K. Thus, the emission in long wavelength region, that is, the RTP emission, of all of the seven host-guest systems, belonged to the same guest molecule **NDOH**, which originated from Dexter triplet-triplet energy transfer (TTET) process. Indeed, **NDOH**@**PDOH** powder exhibited the shortest phosphorescence lifetime of 55.79 ms, while **NDOH**@**PHOH** powder demonstrated the longest phosphorescence lifetime of 165.02 ms, which increased by > 100 ms. The phosphorescence lifetime also increased following the decrease of the extent of electron delocalization on phthalimide derivatives. In other words, most efficient transitions to triplet state could be found on **NDOH**@**PHOH** powder.

At the same time, different relative intensities and lifetimes of TADF and RTP for the host-guest systems resulted in different afterglow color (Fig. 2d). Visible color switches between TADF and RTP could be observed when **PDOH,** **PPOH,** **PEOH** and **POOH** acted as hosts. For instances, the afterglow color of **NDOH**@**PEOH** powder was bluish violet (with CIE coordinates of (0.20, 0.16)) during the delay time from 4 to 20 ms. Then the color changed to pale violet red (with CIE coordinates of (0.36, 0.26)) during the delay time from 20 to 100 ms and finally to orange (with CIE coordinates of (0.53, 0.46)). For the other three host-guest systems, minor changes in afterglow color were observed due to their strong RTP emission. Besides, chromatic CIE coordinates used to describe color changes of afterglow quantitatively were presented in Figs. S11–S17. Thus, tunable afterglow from the same molecule was achieved after the regulation of TTET and triplet-singlet energy transfer (TSET).

**Fig. 3:**
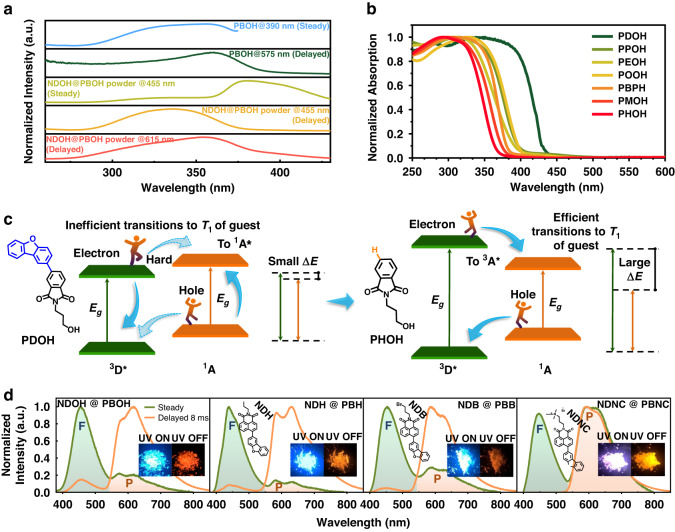
Photophysical properties of host materials and host-guest systems with different terminal groups, and cartoon illustration of exciton transitions with different ΔE values. **a** Excitation spectra of **PBOH** and **NDOH**@**PBOH** powder monitored at maximum fluorescence or phosphorescence emission wavelengths. **b** Solid UV-Vis diffuse reflection spectra of crystalline powder of the host molecules. **c** The schematic diagram of management of triplet exciton transitions to triplet state in Dexter energy transfer process through regulation of band gap and ΔE value. 3D*: Triplet excitons of host; 1D: Ground state of host;1 A: Ground state of guest; 1 A*: Singlet excitons of guest; 3 A*: Triplet excitons of guest. **d** Steady and delayed spectra of **NDOH**@**PBOH,** **NDH**@**PBH,** **NDB**@**PBB** and **NDNC**@**PBNC** powder upon 365 nm light excitation. Inset: The corresponding photographs under UV light and after removal of UV light

To verify whether the afterglow emissions in short and long wavelength regions resulted from different energy transfer process, excitation spectra of host **PBOH** and host-guest system **NDOH**@**PBOH** were first conducted (Fig. 3a). Phosphorescent excitation spectrum of **NDOH**@**PBOH** powder monitored at 615 nm was much similar to that of host **PBOH** rather than its fluorescent excitation spectra, suggesting that the phosphorescence of **NDOH**@**PBOH** powder came from the T_1_ state of **PBOH**, namely, TTET process. Excitation spectra of **NDOH**@**PBOH** powder monitored at 455 nm in steady mode and delayed mode were different, indicating the different photophysical processes. In delayed mode, excitation spectrum monitored at 455 nm had higher maximum excitation energy compared with that monitored at 615 nm for **NDOH**@**PBOH** powder, suggesting that the delayed fluorescence might come from higher triplet state of **PBOH**, namely, TSET process.

In order to get insight into the inherent mechanism of the regulation of TTET and TSET, solid UV-Vis diffuse reflection spectra and phosphorescence spectra of crystalline samples of host molecules were carried out. As presented in Fig. 3b, with the extent of electron delocalization increasing, the absorption edge of the phthalimide derivative was red shifted and the band gap was decreased. In other words, energy gaps between highest occupied crystal orbital (HOCO, analogs of the highest occupied molecular orbital, HOMO) and lowest unoccupied crystal orbital (LUCO, analogs of the lowest unoccupied molecular orbital, LUMO) were decreased with the extent of electron delocalization increasing. Since HOMO → LUMO transition always relates to T_1_ state, large HOMO-LUMO gap will also indicate the large optical gap between T_1_ and ground state. As revealed in the phosphorescence spectra of the host materials, the emission energy of T_1_ also decreased with the extent of electron delocalization increasing (Fig. S18). At 77 K, the maximum emission peaks in the phosphorescence spectra of **PDOH,** **PPOH** and **POOH** should be ascribed to emission from T_2_ of the sample (Fig. S18a), since the monomer phosphorescence emission of them was observed at even longer wavelengths (Fig. S18b). Thus, the existence of the higher triplet state was also proved. Based on the delayed spectra of host materials and guest molecule, ΔE values in different host-guest systems were calculated to be varied from 0.03 to 0.17 eV. It was supposed that larger ΔE with higher T_1_ of host would facilitate hole and electron transfer between the T_1_ states of host and guest materials (Fig. 3c), giving rise to efficient TTET and inefficient TSET. Without changing the guest molecule, the larger energy differences between T_1_ of host materials and the guest molecule also indicate the blue-shifted phosphorescence emission of host materials, which will lead to a larger spectral overlap integral between the phosphorescence emission of host materials and the absorption of the guest molecule. In this way, management of triplet excitons was achieved. When a weak π-electron donating group, such as methoxy group, was connected to phthalimide, and the resultant ΔE of host-guest system was > 0.07 eV, more efficient transition to triplet state rather than singlet state would occur with stronger RTP emission intensity.

In addition to the direct manipulation of conjugated structures for band gap tuning, an effective approach is the regulation of crystal stacking modes. As presented in Fig. S18b, the completely different phosphorescence emission in aggregated state and monomer state indicated the great influence of aggregation on the energy levels of the triplet states of **PDOH,** **PPOH** and **POOH**. Accordingly, by changing the stacking modes of host materials, it becomes possible to control the triplet states of these materials and subsequently manipulate triplet excitons. We tried to further verify the mechanism by changing the crystal stacking modes. Bromine substituted phthalimide was chosen due to its obvious heavy atom effect, which assisted in efficient RTP on **NDOH**@**PBOH** powder with the highest phosphorescence quantum yield of 3.9% among the seven host-guest systems. Though terminal groups on the N-substituted alkyl chains of phthalimide derivatives did not influence the conjugated structures, intermolecular interactions formed by them could change the crystal packing modes, thus changing the band gaps. Hydroxyl groups on the alkyl chains of **PBOH** and **NDOH** were replaced by hydrogen atom, bromine atom and quaternary ammonium bromide group. With the terminal groups changed from hydroxyl group to hydrogen atom, then to bromine atom and finally to quaternary ammonium bromide group, the RTP spectra of the samples were gradually blue shifted (Fig. S19). At the same time, the same changing trend could be found in absorption edges of the samples (Fig. S20). It suggested that when **PBNC** acted as host, most efficient TTET should happen.

Hence, here we mainly compared the relative intensities of TADF and RTP of **NDOH**@**PBOH,** **NDH**@**PBH,** **NDB**@**PBB** and **NDNC**@**PBNC** powder (Figs. S21–S29). Figure 3d revealed that in comparison with **NDOH**@**PBOH** powder, **NDH**@**PBH** and **NDB**@**PBB** powder exhibited evidently and relatively weaker intensities of TADF, while no TADF emission could be found from the delayed spectrum of **NDNC**@**PBNC** powder. Interestingly, the strongest RTP emission was observed on **NDNC**@**PBNC** powder with quantum yield of 13.9%, which was at least twice of that of other host-guest systems. Its shorter phosphorescence lifetime of 49.17 ms compared to that of **NDOH**@**PBOH** powder (159.04 ms) suggested that synergism of heavy atom effect and efficient TTET were responsible for the brightest RTP. With the highest T_1_ of **PBNC,** **NDNC**@**PBNC** exhibited the largest ΔE and most efficient transition to triplet state for triplet exciton of **PBNC**, while the transition to singlet state was absent as demonstrated by the delayed spectrum without TADF emission. Apparently, our mechanism of management of triplet excitons transitions was further verified.

**Fig. 4:**
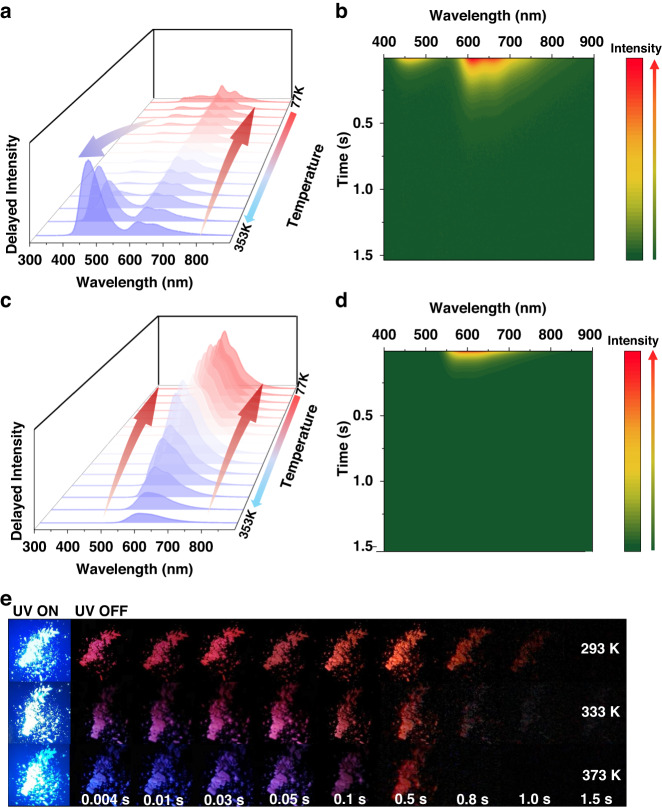
Photophysical properties of NDOH@POOH and NDNC@PBNC at different temperatures. **a** Delayed spectra of **NDOH**@**POOH** from 77 to 353 K. λ_ex_ = 365 nm. **b** The time-resolved delayed spectra (λ_ex_ = 365 nm, at 298 K) of **NDOH**@**POOH**. **c** Delayed spectra of **NDNC**@**PBNC** from 77 to 353 K. λ_ex_ = 365 nm. **d** The time-resolved delayed spectra (λ_ex_ = 365 nm, at 298 K) of **NDNC**@**PBNC**. **e** Photographs of **NDOH**@**POOH** under UV light and after removal of UV light taken at 293, 333 and 373 K

Admittedly, the TADF and RTP nature of host-guest systems needed to be further proved by temperature-dependent emission spectra. As consequence, in situ temperature-dependent delayed spectra of host-guest systems were explored, which also established the temperature responsive behaviors of exciton transitions. **NDOH**@**POOH** powder with strong emission in both short and long wavelength regions was first investigated carefully. Figure 4a shows the delayed spectra of **NDOH**@**POOH** powder from 77 to 353 K. It was clear that the emission around 460 nm was thermally activated while the emission around 610 nm was thermally quenched, which corresponded to TADF and RTP nature of the sample, respectively. The line chart displaying the intensity changes at 460 and 610 nm with increasing temperature illustrated the different tendencies clearly (Fig. S30). The intensity decay curves at 460 nm also revealed a slower decay rate at higher temperature (Fig. S31). Similar temperature responsive behaviors could be found in other host-guest systems using **PDOH,** **PPOH,** **PEOH,** **POOH,** **PBOH,** **PMOH,** **PHOH,** **PBH** and **PBB** as host (Figs. S32–S49). It is worth mentioning that the delayed emission of **NDOH**@**PDOH** powder around 460 nm exhibited a phenomenon where it was initially thermally quenched and subsequently became thermally activated. The delayed emission spectra of **NDOH**@**PDOH** and **PDOH** powder at 77 K was similar (Figure S33), indicating that the emission of **NDOH**@**PDOH** powder around 460 nm at 77 K should be attributed to phosphorescence emission from **PDOH**. Thus, when temperature was enhanced from 77 K, the phosphorescence was initially thermally quenched. However, at higher temperature, the endothermic Förster TSET process from **PDOH** to **NDOH** was promoted, resulting in TADF emission of **NDOH**. And the emission of **NDOH**@**PDOH** powder around 460 nm subsequently became thermally activated. When **PBNC** acted as host, no thermally activated emission was observed (Figs. S50–S53). Taking **NDNC**@**PBNC** powder as example, emission intensity around 448 and 599 nm both decreased with temperature increasing (Fig. 4c), further confirming that no TADF emission could be found on it. The time-resolved delayed spectra of **NDOH**@**POOH** powder at room temperature exhibited obvious two emission peaks with different decay rates (Fig. 4b). Indeed, similar phenomenon could be observed in other host-guest systems with TADF and RTP dual emission (Figs. S54–S62), while single emission band was observed on the time-resolved delayed spectra of **NDNC**@**PBNC** powder (Fig. 4d), as well as other **PBNC**-based host-guest systems (Fig. S63). At high temperature, triplet excitons of host absorbed surrounding thermal energy to undergo RISC process. Thus, transitions to singlet state were promoted at high temperature, suggesting that the management of triplet excitons could also be achieved by adjusting temperature. On the basis of it, the heat effect on afterglow color was studied. As presented in Fig. 4e, for **NDOH**@**POOH** powder, the afterglow colors during 4–10 ms after removal of UV light changed from pink to purple and blue with temperature rising from 293 to 333 and 373 K. It should be ascribed to the promotion of transitions to singlet state for triplet excitons of host and the enhancement of TADF intensity. The different afterglow colors could also be illustrated quantitatively by CIE coordinates (Fig. S64).

### Crystal Structures Analysis and Theoretical Calculation

**Fig. 5:**
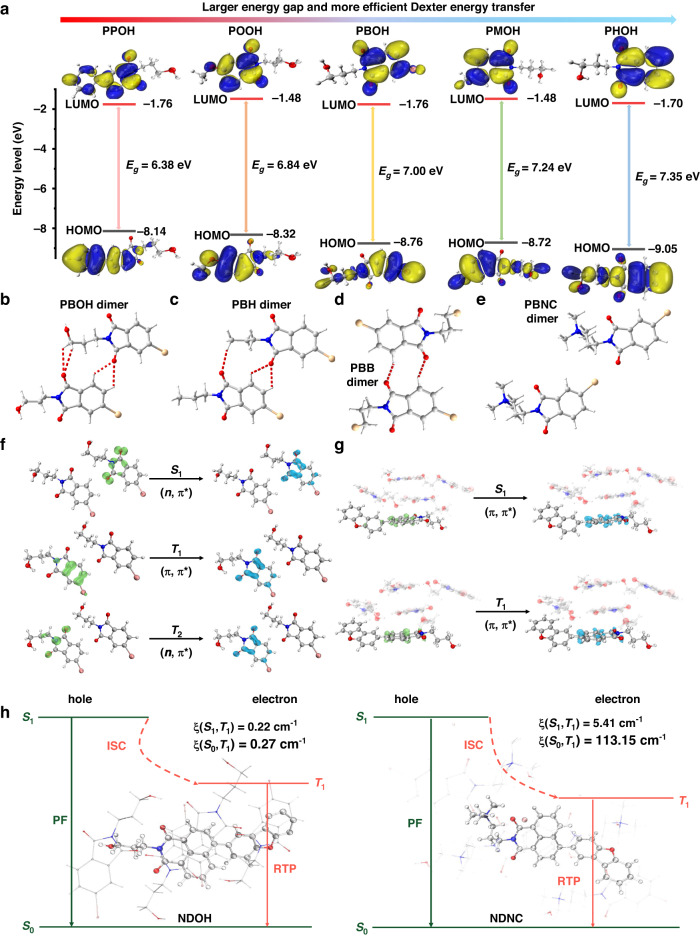
Theoretical calculation and crystal structures analysis. **a** Frontier molecular orbitals and energy gaps of **PPOH,** **POOH,** **PBOH,** **PMOH** and **PHOH** based on the monomer in single crystals. **b**–**e** C-H···O interactions (red dash lines) in parallel dimers of single crystals of **PBOH** (**b**) **PBH** (**c**) **PBB** (**d**) and **PBNC** (**e**). **f**, **g** Hole-electron analyses of S_0_ → S_1_, T_1_ → S_0_ and T_2_ → S_0_ transitions of PBOH dimer (**f**) and S_0_ → S_1_ and S_0_ → T_1_ transitions of **NDOH** coupled with **PBOH** crystal cell (**g**) and the corresponding transition characteristics of them. **h** Diagrams of the calculated energy levels and the SOC constants between singlet and triplet excited states of **NDOH** coupled with **PBOH** crystal cell and **NDNC** coupled with **PBNC** crystal cell

Next, the single-crystal structure and molecular stacking of the host molecules were examined carefully. Theoretical calculations were also conducted so as to provide more proofs for the mechanism. Single crystals of **PPOH,** **POOH,** **PBOH,** **PMOH,** **PHOH,** **PBH** and **PBB** were cultured from mixture solvents of chloroform and methanol, while that of **PBNC** was cultured from methanol. Frontier molecular orbitals of **PPOH,** **POOH,** **PBOH,** **PMOH** and **PHOH** based on the molecular configuration in single crystals were calculated to investigate the influence of conjugated structures on molecular orbitals. When the substituents on 4-position of phthalimide derivatives changed from phenyl group to hydrogen atom, the extent of electron delocalization was decreased and lower HOMO could be found (Fig. 5a), resulting in larger energy gap as identified by the solid UV-vis diffuse reflection spectra.

The abundant intermolecular interactions in phthalimide derivatives could provide a rigid environment to suppress non-radiative transitions and isolate the luminogen from oxygen for the afterglow emission. Along with energy transfer process and triplet excitons from the phthalimide derivatives, bright afterglow could be observed without the photoactivated process. The same diffraction peaks existing on powder X-ray diffraction (PXRD) patterns of single crystals and host-guest systems suggested the similar packing modes between them (Fig. S65). Molecules of **PBOH,** **PBH,** **PBB** and **PBNC** stack layer by layer in their single crystals (Fig. S66). Due to the presence of hydroxyl groups, strong -O-H···H hydrogen bonds with distances of 1.99 and 2.01 Å could be found in **PBOH** crystal, implying the tighter stacking mode. It could also be proved by the high melting point of **PBOH** crystal compared with other crystals, as demonstrated by DSC curves (Fig. S67). The introduction of the hydroxyl group into the host molecules enabled the formation of strong -O-H···H hydrogen bonds in the aggregated state of the host molecules. When the materials served as hosts for the guest molecule, the resultant rigid matrix consequently prolonged the RTP lifetime. As presented in Fig. 5b–e, **PBOH** dimer exhibited four -C-H···O bonds ranging from 2.53 to 2.92 Å, and **PBH** dimer exhibited three -C-H···O bonds ranging from 2.52 to 2.81 Å, while only two -C-H···O bonds were found in **PBB** dimer and no -C-H···O bond was found in **PBNC** dimer. Strong intermolecular interactions will extend the conjugated structures to some extent, thus reducing band gap and ΔE value, which is unfavorable to Dexter energy transfer process and transitions to triplet state (Fig. 3c).

Hole-electron analyses were also manipulated to study the electronic structure of the singlet and triplet excited state. They were performed via Multiwfn^55^ and VMD according to literature^56^. Figure 5f revealed that for **PBOH** dimer, S_0_ → S_1_ excitation should be mainly n-π* transition, and T_1_ → S_0_ should be π-π* transition, while T_2_ → S_0_ should be n-π* transition. The structures of **NDOH** coupled with **PBOH** crystal cell in the ground state and triplet state were optimized employing the ONIOM model. **NDOH** was set as high layer and **PBOH** crystal cell was set as low layer. Accordingly, both S_0_ → S_1_ and S_0_ → T_1_ transitions were found to exhibit π-π* characteristic (Fig. 5g). Efficient ISC occurred between ^1^nπ* and ^3^ππ* as well as ^3^nπ* and ^1^ππ*. ISC process between S_1_ and T_1_ of **NDOH** was less efficient due to the same transition characteristic of them. It was speculated that TSET process might occur from T_2_ of host materials to S_1_ of the guest molecules. TTET process could proceed efficiently between T_1_ of **NDOH** and **PBOH**, leading to strong RTP emission. In addition, transition characteristics of other host and guest molecules and SOC constants between singlet states and low-lying triplet states (Fig. 5h and Figs. S68–S83) also supported the ease of phosphorescence emission process of **NDOH,** **NDH** and **NDB** coupled with host molecules, and the most efficient S_1_ → T_1_ and T_1_ → S_0_ transitions of **NDNC**. For instance, the SOC constants of (S_1_, T_1_) and (T_1_, S_0_) of **NDOH** were 0.22 and 0.27 cm^-1^, respectively, and the corresponding values of **NDNC** increased to 5.41 and 113.15 cm^-1^, which were hundreds of times larger than those of other guest molecules. It should arise from the heavy atom effect of bromide ions, which contributed to the largest phosphorescence quantum yield of **NDNC**@**PBNC** powder. Indeed, **NDB**@**PBB** powder exhibited a high phosphorescence quantum yield of 5.1%, which was only lower than that of **NDNC**@**PBNC** powder among all the host-guest systems, further validating the important role of heavy atom effect in the phosphorescence emission. Furthermore, the heavy atom effect of the bromide ion of **NDNC** could also enhance the SOC between S_1_ and T_1_ as revealed by the theoretical calculation result. Thus, the ISC process in **NDNC** was promoted, reducing the fluorescence emission efficiency. With the large SOC between T_1_ and S_0_, TADF emission was hardly observed when **NDNC** acted as the guest molecule (Figs. S22–S25).

### A summary of management of triplet excitons

**Fig. 6:**
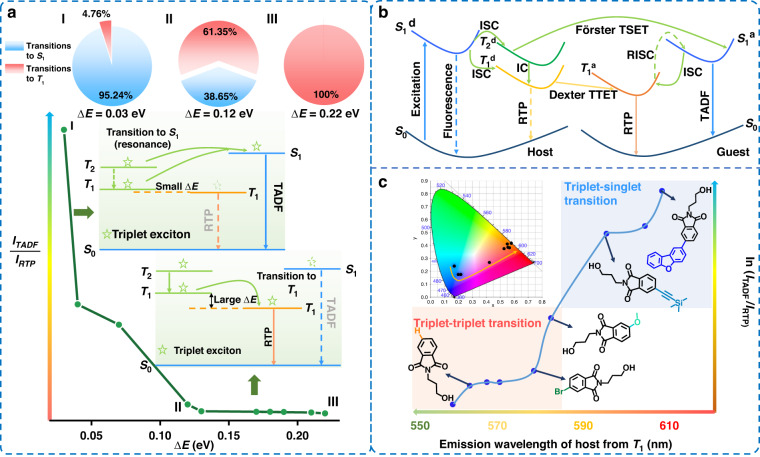
A summary of management of triplet excitons. **a** The pie charts, line chart and schematic diagram describing the relationship between ΔE values and triplet exciton transitions. **b** Schematic diagram of the energy transfer process in host-guest systems. **c** The line chart based on the emission wavelengths of host molecules from T_1_ and ln(I_TADF_/I_RTP_). Inset: CIE chromaticity coordinates based on the delayed spectra of host-guest systems

Photophysical properties of all of the sample were characterized (Figs. S84–S92) and summarized in Tables S1 and S2. The host-guest systems with low PLQYs of the energy donors (**PBOH,** **PMOH,** **PHOH,** **PBH,** **PBB** and **PBNC**) showed high phosphorescence quantum yields, indicating the occurrence of non-radiative energy transfer rather than radiative energy transfer. For example, **PBNC** crystal exhibited low PLQY of < 0.1%. However, efficient TTET was observed when **PBNC** acted as the host. It provides further evidence for the occurrence of Dexter energy transfer, which is not dependent on the radiative oscillator strength. The longest phosphorescence lifetime is found when the molar ratio between **NDOH** and **PBOH** was 1:10,000. The shorter phosphorescence lifetime of the host-guest systems with higher concentration of **NDOH** should be ascribed to the concentration quenching effect of triplet excitons, which consumed triplet excitons in non-radiative pathways. A line chart based on ΔE value and intensity ratio between TADF and RTP was then presented (Fig. 6a). Obviously, efficient transitions to triplet state could be observed with a large ΔE value. The pie charts also described the estimated percentages of transitions to triplet state with different ΔE values. Overall, the management of triplet excitons in host-guest systems can be summarized as follows (Fig. 6b). First, after photoexcitation process, excitons in lowest singlet excited states of donor (S_1_^d^) were formed, which could be converted to triplet excitons (T_1_^d^ and T_2_^d^). Then, Förster TSET process might occur and generate TADF emission. Dexter TTET process could occurr between T_1_^d^ and lowest triplet excited states of acceptor (T_1_^a^), generating RTP emission. Decreased extent of electron delocalization with larger band gap and ΔE value with higher T_1_^d^ energy could promote the TTET process, and also accounted for less efficient TSET process, as revealed by the intensity ratios between TADF and RTP. The management of triplet excitons was thus achieved and the mechanism was clear. A line chart of the relationship between emission wavelength of T_1_^d^ and ln(I_TADF_/I_RTP_) was then provided (Fig. 6c), where I_TADF_ and I_RTP_ were maximum emission intensities of TADF and RTP in the 8-ms-delayed spectra of host-guest systems, respectively. It will be conducive to predict the triplet-triplet and triplet-singlet transitions on the basis of the phosphorescence emission of host materials. Benefiting from it, we can just finely regulate the movement of triplet excitons and afterglow color by changing the host structure, as well as develop efficient TADF or RTP materials after the regulation of ΔE for promotion of FRET or Dexter energy transfer. We also replaced the dibenzofuran group in **NDOH** with a pyrene group to synthesize **NPOH** and modify the energy of the T_1_ state of the guest molecule. As demonstrated in Figs. S93a, S93b and S93d, when **PDOH** acted as the host material, the host-guest system showed concurrent strong TADF and RTP emission at room temperature. As expected, when **PBOH** with larger energy gap was employed as the host material, only strong RTP emission could be observed in the host-guest system at room temperature (Figs. S93a, S93c and S93e), further validating the effectiveness and feasibility of the proposed approach for managing triplet excitons.

### Practical applications

**Fig. 7:**
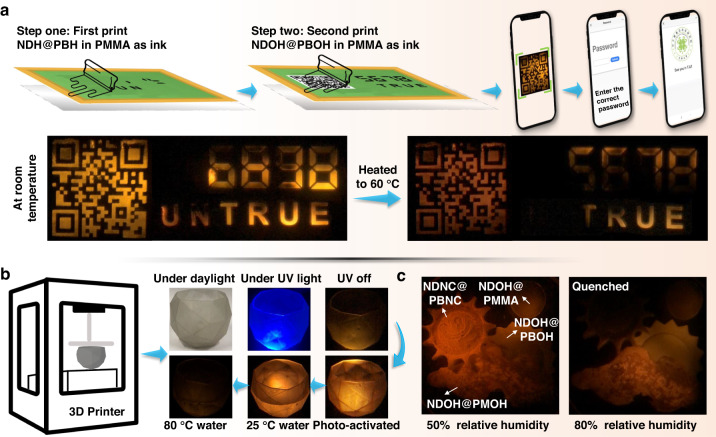
Practical applications based on the host-guest systems. **a** Upper: The schematic diagram of the screen printing process. Lower: Afterglow photographs of the glass taken at room temperature and 60 ^o^C. **b** The schematic diagram of 3D print based on **NDOH**@**PBOH** to get a temperature-sensitive bowl. **c** Photographs of afterglow of the three-dimensional models constructed by **NDOH**@PMMA and **NDNC**@**PBNC,** **NDOH**@**PMOH** and **NDOH**@**PBOH** in agarose, respectively

Finally, taking advantage of the different temperature and humidity responsive behaviors of the host-guest systems, a series of applications were carried out. Screen printing has been widely applied in different industries and a wide variety of inks can be used for it. In view of the high melt point of **PBOH** and low melt point of **PBH**, phosphorescence of **NDOH**@**PBOH** can still be observed at high temperature while that of **NDH**@**PBH** is quenched. Therefore, using **NDOH**@**PBOH** and **NDH**@**PBH** as luminophores in inks, multiple anti-counterfeiting could be realized. PMMA in THF was used to provide viscosity for the ink. Upper part in Fig. 7a shows the schematic diagram of the screen printing process.

First, **NDH**@**PBH** was dissolved in THF solution of PMMA as ink and the pattern was recorded onto the glass layer. Secondly, **NDOH**@**PBOH** was dissolved in THF solution of PMMA as ink and another pattern was recorded. Under UV irradiation, the QR code, the number “6898” and the word “untrue” could be read. After turning off UV light at room temperature, the same information with no background noise was read (Left of lower part of Fig. 7a). At this time, if we scan the QR code and enter the wrong password “6898”, no document would be obtained. After heating the glass to 60 ^o^C, only the QR code, the number “5678” and the word “true” could show afterglow (Right of lower part of Fig. 7a). After scanning the QR code and entering the correct password “5678”, the document could be downloaded.

Furthermore, the introduction of the hydroxyl group improved the solubility of the materials in alcohol solvents, thus contributing to environmentally friendly applications. For example, information could also be encrypted by spraying the ethanol solution of **PBNC** and **NDNC** (Fig. S94). There are great advantages for 3D printing in manufacturing something with small size and complex structure. The addition of phosphorescent chromophores would further expand the applications of 3D printing. As a result, **NDOH**@**PBOH** was added into photocurable resin to print a temperature-sensitive bowl (Fig. 7b). The bowl exhibited blue fluorescence and orange afterglow at room temperature, which could be further photo-activated. When holding water at 25 ^o^C, the bowl still showed orange afterglow. When holding hot water at 80 ^o^C, the bowl showed almost no afterglow. Thus, the printed bowl could be used as a container for liquids and heat warning (≥80 ^o^C). Besides, in consideration of the water-solubility of **NDNC** and **PBNC,** **NDNC**@**PBNC** was used for humidity monitoring. Four three-dimensional models were constructed from **NDOH**@PMMA and **NDNC**@**PBNC,** **NDOH**@**PMOH** and **NDOH**@**PBOH** in agarose (Fig. 7c). Afterglow of them at 50% relative humidity was presented on the left of Fig. 7c. When the relative humidity was risen to 80%, afterglow from **NDNC**@**PBNC** was quenched because of the invasion of water to the host-guest system. However, for **NDOH**@PMMA and **NDOH**@**PMOH** and **NDOH**@**PBOH** in agarose, the hydrophobicity of the compounds made the afterglow from them more difficult to be quenched by water. Therefore, **NDNC**@**PBNC** model could monitor the high humidity (≥80% relative humidity).

## Discussion

In this work, we finely managed the triplet excitons and tuned the afterglow color of the same molecule by host engineering for the first time. The transitions to singlet or triplet states could be managed by controlling the TSET and TTET process, which could be evaluated according to TADF and RTP emission intensity. With the electron donating groups on host molecules changing from dibenzofuran to hydrogen atom, energy differences between T_1_ of host materials and guest molecule increased from 0.03 to 0.17 eV, and intensity ratios between TADF and RTP from **NDOH** decreased by 200 times. Thus, the mechanism was deeply understood. Larger ΔE with higher T_1_ facilitated Dexter TTET process and weakened Förster TSET process, which could be realized by shortening the conjugation length. The mechanism was further verified by regulating crystal stacking modes to tune the band gap and ΔE value. **PBNC** crystal with loosest stacking mode and largest ΔE (0.22 eV) exhibited most efficient TTET process when serving as host and no transition to singlet state was observed. On the basis of the multiple stimulus-responsive exciton transitions, the host-guest systems were utilized in screen printing, information encryption, 3D printing and model construction for multiple anti-counterfeiting and temperature or humidity monitoring.

In our research, it was found that the management of triplet exciton transition could be achieved by simultaneously regulating the Dexter and Förster energy transfer processes. Consequently, to develop organic RTP materials, we could design energy transfer systems with efficient Dexter TTET process. The energy difference between triplet states of energy donor and acceptor should be enlarged to enhance Dexter TTET efficiency and obtain efficient RTP emission. One way to increase this energy difference is by shortening the conjugation length of the host molecules. It is convenient to enhance RTP emission of the guest molecule without altering the emission wavelength by host engineering. Furthermore, a rigid environment is advantageous in suppressing non-radiative transitions and isolating luminogens from oxygen. So it is beneficial for the host molecules to incorporate appropriate functional groups that can manipulate abundant intermolecular interactions and create a rigid environment for efficient RTP emission. This work will also push the boundaries for the development of pure organic smart afterglow materials with tunable afterglow colors through management of triplet excitons, since Dexter energy transfer and FRET can be regulated by changing the ΔE values and will result in efficient TADF or RTP emission. Also, the prediction of triplet-singlet and triplet-triplet transitions can be realized, which is of great importance for practical applications.

## Materials and methods

### Synthesis

The synthesis route to the target compounds is presented in Scheme S1. The experimental details on the synthesis are provided in the Supplementary Information.

### Characterization

^1^H and ^13^C NMR spectra were recorded on a 400 MHz Bruker Ascend spectrometer. High resolution mass spectrometry (HRMS) data were obtained on Bruker UltiMate3000 & Compact. UV-Vis spectra were measured on a Shimadzu UV-2600 and an Ocean Optics QE65 Pro spectrometer. Time-resolved delayed spectra were also collected from an Ocean Optics QE65 Pro spectrometer. If not otherwise specified, the delayed spectra mentioned in this work were measured at 8 ms after stopping excitation. Photoluminescence (PL) quantum yields and lifetimes were determined with FLS1000 spectrometer. The powder X-ray diffraction patterns were recorded by a Rigaku MiniFlex600. The single-crystal X-ray diffraction data was collected in an XtaLAB SuperNova X-ray diffractometer. Differential scanning calorimetry (DSC) were measured by a Netzsch DSC-214. High-performance liquid chromatography (HPLC) spectra were recorded on an Agilent 1100 HPLC chromatograph.

### Theoretical methods

TD-DFT calculations were performed on Gaussian 09 program (Revision D01)^57^ with the functional of M06-2X and 6-311 + G(d, p) basis sets and the T_1_ geometry was optimized via closed shell method at S_0_ configuration. Hole-electron analysis was performed via Multiwfn^47^ and VMD. The SOC constants between S_0_/S_1_ and triplet states were determined by PySOC package.

### Supplementary information

supporting information

crystal data

crystal data

crystal data

crystal data

crystal data

crystal data

crystal data

crystal data

## Data Availability

The authors declare that the data supporting the findings of this study are available within the article and its Supplementary Information file. All data are available from the authors upon reasonable request.
