# Supplementary material for: Management of triplet excitons transition: fine regulation of Förster and dexter energy transfer simultaneously
Source: Light Sci Appl. 2024 Jan 30;13:35. doi: 10.1038/s41377-023-01366-1 (PMC10828450; doi:10.1038/s41377-023-01366-1)
Supplement: Supplementary file 1 — supporting information [file 41377_2023_1366_MOESM1_ESM.pdf]

Supplementary Information for

Management of Triplet Excitons Transition: Fine Regulation of Förster and Dexter Energy Transfer Simultaneously

Jiaqiang Wang,[1] Yujie Yang,[1] Xinnan Sun,[2] Xiaoning Li,[1] Liyao Zhang,[2]* and Zhen Li[1,3,4,5]*

[1]Institute of Molecular Aggregation Science, Tianjin University, Tianjin 300072, China.

[2]School of Life Sciences, Tianjin University, Tianjin 300072, China

[3]Hubei Key Lab on Organic and Polymeric Opto-Electronic Materials, Department of Chemistry, Wuhan University, Wuhan 430072, China.

[4]Wuhan National Laboratory for Optoelectronics, Huazhong University of Science and Technology, Wuhan 430072, China

[5]Joint School of National University of Singapore, Tianjin University, International Campus of Tianjin University, Binhai New City, Fuzhou 350207, China.

E-mail: lyzhang26@whu.edu.cn; lizhen@whu.edu.cn

Table of Contents

I Experimental Methods S2

II Results and Discussion.. S11

III References... S86

I. Experimental Methods

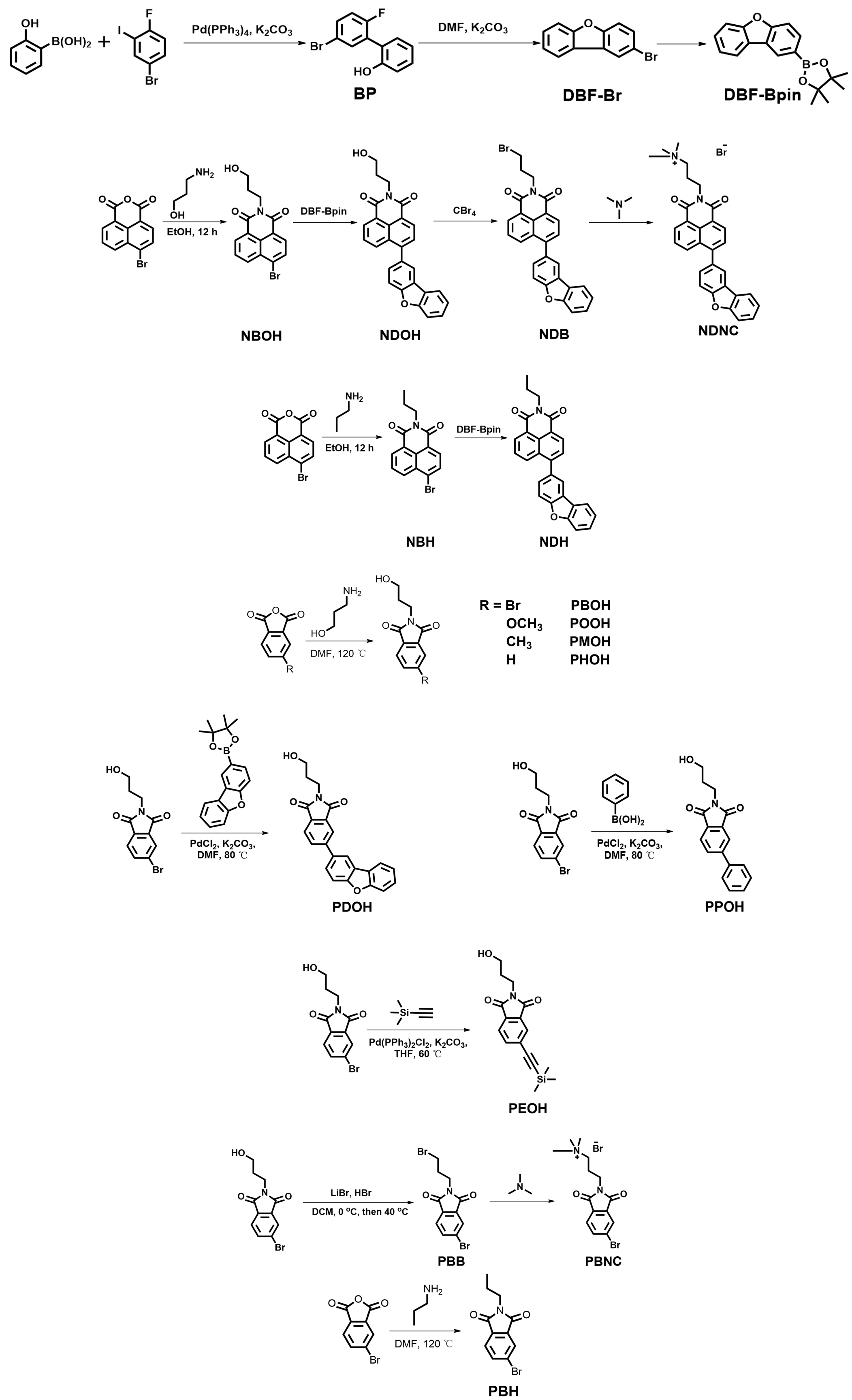

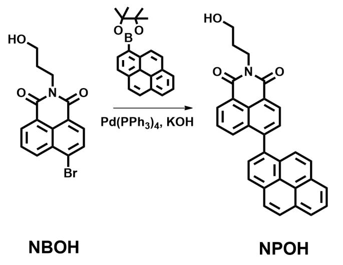

Scheme S1. Synthetic approach to the target compounds.

Synthesis

Preparation of BP

To a 250 ml Schlenk tube containing 2-Hydroxybenzeneboronic acid (3.32 g, 24 mmol) and 4-bromo-1-fluoro-2-iodobenzene (6.00 g, 20 mmol), K_2CO_3 (4.14 g, 30 mmol), $Pd(PPh_3)_4$ (0.12 g, 0.1 mmol), 40 mL toluene, 20 mL ethanol and 20 mL deionized water were added under nitrogen atmosphere. The solution was refluxed for 24 h and then poured into water. The mixture was extracted with ethyl acetate (EA) for several times and washed with brine. After dried over anhydrous sodium sulfate, the solvent was removed in vacuo, and the pure product was isolated by column chromatography on silica gel. The crude product was then recrystallized from ethanol to give BP (2.6 g, 50%) as a light yellow solid. ^{1}H NMR (400 MHz, DMSO-d_6) δ (ppm): 9.73 – 9.68 (m, 1H), 7.61 – 7.50 (m, 2H), 7.29 – 7.21 (m, 2H), 7.21 – 7.15 (m, 1H), 6.99 – 6.92 (m, 1H), 6.92 – 6.84 (m, 1H). ^{13}C NMR (101 MHz, DMSO-d_6) δ (ppm): 160.40, 157.95, 155.14, 134.63, 134.59, 132.15, 132.06, 131.39, 130.25, 129.23, 129.06, 121.46, 119.49, 118.32, 118.07, 116.15, 116.09, 116.05.

Preparation of DBF-Br

BP (1.34 g, 5 mmol) and potassium carbonate (1.38 g, 10 mmol) were stirred in a two-necked round-bottomed flask filled with DMF (20 mL) under a nitrogen atmosphere at 140 °C overnight. After cooling to room temperature, the reaction mixture was poured into water and filtered. The crude product was purified by column chromatography and recrystallized from ethanol to afford **DBF-Br** as a white product (1.0 g, 81 %). ^{1}H NMR (400 MHz, DMSO-d_6) δ (ppm): 8.48 – 8.42 (m, 1H), 8.25 – 8.17 (m,

1H), 7.77 – 7.63 (m, 3H), 7.62 – 7.53 (m, 1H), 7.44 (tt, *J* = 7.4, 1.3 Hz, 1H). ^{13}C NMR (101 MHz, DMSO-d_6) δ (ppm): 156.39, 154.75, 130.52, 128.93, 126.36, 124.45, 123.92, 123.03, 122.23, 115.70, 114.23, 112.30.

Preparation of DBF-Bpin

To a 250 ml Schlenk flask containing **DBF-Br** (1.24 g, 5 mmol) and bis(pinacolato)diboron (1.52 g, 6 mmol), Pd(dppf)Cl_2 (0.03 g, 0.04 mmol), KOAc (0.98 g, 10 mmol) and 15 mL of 1,4-dioxane were added under argon atmosphere. The suspension was stirred at 80 °C for 12 h. After completion of the reaction, the mixture was cooled to room temperature and the filtrate was then evaporated under vacuum and purified by column chromatography. After recrystallization from petroleum ether, **DBF-Bpin** (1.2 g, 80 %) was afforded as a white product. ^{1}H NMR (400 MHz, DMSO-d_6) δ (ppm): 8.51 – 8.46 (m, 1H), 8.30 – 8.23 (m, 1H), 7.83 (dd, *J* = 8.3, 1.3 Hz, 1H), 7.75 – 7.66 (m, 2H), 7.58 – 7.49 (m, 1H), 7.46 – 7.36 (m, 1H), 1.33 (s, 12H). ^{13}C NMR (101 MHz, DMSO-d_6) δ (ppm): 158.07, 155.94, 134.27, 128.31, 128.24, 123.94, 123.86, 123.72, 121.99, 112.11, 111.80, 84.26, 25.21.

Preparation of NBOH

To a stirred solution of 4-bromo-1,8-naphthalic anhydride (2.77 g, 10 mmol) in EtOH (30 mL) was added 3-aminopropanol (0.83 g, 11 mmol). The resulting mixture was heated at the refluxing temperature for 12 h. After completion, the reaction mixture was cooled to room temperature and concentrated under vacuum to give a solid, which was further purified by column chromatography. The crude product was then recrystallized from EtOH to give the pure product **NBOH**. White solid (yield 92%, 3.1 g). ^{1}H NMR (400 MHz, DMSO-d_6) δ (ppm): 8.47 (dd, *J* = 7.3, 1.1 Hz, 1H), 8.42 (dd, *J* = 8.5, 1.1 Hz, 1H), 8.22 (d, *J* = 7.9 Hz, 1H), 8.12 (d, *J* = 7.8 Hz, 1H), 7.91 (dd, *J* = 8.5, 7.3 Hz, 1H), 4.53 (t, *J* = 5.1 Hz, 1H), 4.10 – 4.02 (m, 2H), 3.51 (td, *J* = 6.3, 5.1 Hz, 2H), 1.84 – 1.73 (m, 2H). ^{13}C NMR (101

MHz, DMSO-d_6) δ (ppm): 163.25, 163.20, 132.90, 131.90, 131.71, 131.28, 130.10, 129.47, 129.14, 128.58, 123.13, 122.36, 59.46, 38.21, 31.33.

Preparation of NDOH

To a 100 ml Schlenk tube containing **NBOH** (1.00 g, 3 mmol) and **DBF-Bpin** (1.32 g, 4.5 mmol), KOH (0.34 g, 6 mmol), $Pd(PPh_3)_4$ (0.06 g, 0.05 mmol), 15 mL of THF and 3 mL of deionized water were added under nitrogen atmosphere. The solution was refluxed for 24 h and then poured into water. The mixture was extracted with dichloromethane for several times and washed with brine. After dried over anhydrous sodium sulfate, the solvent was removed in vacuo, and the pure product was isolated by column chromatography on silica gel. The crude product was then recrystallized from ethanol to give **NDOH** (1 g, 75%) as a green solid. ^1H NMR (400 MHz, DMSO-d_6) δ (ppm): 8.58 – 8.50 (m, 2H), 8.36 (dd, J = 1.8, 0.6 Hz, 1H), 8.29 (dd, J = 8.5, 1.2 Hz, 1H), 8.25 – 8.18 (m, 1H), 7.93 – 7.87 (m, 2H), 7.84 (dd, J = 8.5, 7.3 Hz, 1H), 7.80 – 7.73 (m, 1H), 7.67 (dd, J = 8.5, 1.9 Hz, 1H), 7.62 – 7.54 (m, 1H), 7.43 (td, J = 7.4, 1.0 Hz, 1H), 4.55 (t, J = 5.2 Hz, 1H), 4.19 – 4.10 (m, 2H), 3.54 (td, J = 6.3, 5.1 Hz, 2H), 1.89 – 1.78 (m, 2H). ^{13}C NMR (101 MHz, DMSO-d_6) δ (ppm): 163.94, 163.73, 156.45, 155.84, 146.40, 133.78, 132.81, 131.19, 130.75, 130.05, 129.81, 128.90, 128.56, 128.40, 128.00, 124.57, 123.80, 123.06, 122.91, 122.10, 121.74, 112.36, 112.27, 59.50, 38.13, 31.48. HRMS (ESI) m/z calcd for $C_{27}H_{20}NO_4^+$ $(M+H)^+$ 422.1392, found 422.1387.

Preparation of NDB

Carbon tetrabromide (1.37 g, 4 mmol) was added to a stirred solution of **NDOH** (0.84 g, 2 mmol), triphenylphoshpine (1.05 g, 4 mmol) and anhydrous THF (30 mL) in portions within 40 min at 0 °C. The solution was stirred for 3 h under nitrogen at room temperature and then wash with water (100 mL) and brine (100 mL). After dried over anhydrous sodium sulfate, the solvent was removed in vacuo, and the pure product was isolated by column chromatography on silica gel. The crude product is

recrystallized from ethanol to get **NDB** as a green solid (1 g, 98%). ^{1}H NMR (400 MHz, DMSO-d_6) δ (ppm): 8.62 – 8.50 (m, 2H), 8.37 (t, J = 3.3 Hz, 1H), 8.34 – 8.26 (m, 1H), 8.26 – 8.19 (m, 1H), 7.96 – 7.81 (m, 3H), 7.81 – 7.74 (m, 1H), 7.68 (dt, J = 8.4, 2.6 Hz, 1H), 7.63 – 7.54 (m, 1H), 7.48 – 7.39 (m, 1H), 4.26 – 4.17 (m, 2H), 3.65 (t, J = 6.7 Hz, 2H), 2.30 – 2.19 (m, 2H). ^{13}C NMR (101 MHz, DMSO-d_6) δ (ppm): 164.13, 163.91, 156.47, 155.86, 146.46, 133.80, 132.86, 131.22, 130.78, 130.09, 129.83, 128.92, 128.59, 128.03, 124.60, 123.82, 123.75, 123.08, 122.14, 121.85, 112.39, 112.29, 39.25, 32.85, 31.39. HRMS (ESI) m/z calcd for $C_{27}H_{19}BrNO_3^+$ $(M+H)^+$ 484.0543, found 484.0536.

Preparation of NDNC

NDB (0.48 g, 1 mmol), trimethylamine (4.2 M in ethanol, 4.8 mL) and ethanol (20 mL) were refluxed at 80 °C for 24 h. The solvent was removed in vacuo, and the pure product was isolated by column chromatography on silica gel. The crude product is recrystallized from ethanol to get **NDNC** as a green solid (0.4 g, 72%). ^{1}H NMR (400 MHz, DMSO-d_6) δ (ppm): 8.65 – 8.56 (m, 2H), 8.41 – 8.32 (m, 2H), 8.30 – 8.23 (m, 1H), 7.98 – 7.87 (m, 3H), 7.83 – 7.77 (m, 1H), 7.72 – 7.65 (m, 1H), 7.65 – 7.56 (m, 1H), 7.46 (td, J = 7.5, 0.9 Hz, 1H), 4.20 (t, J = 6.3 Hz, 2H), 3.52 – 3.44 (m, 2H), 3.05 (s, 9H), 2.24 – 2.12 (m, 2H). ^{13}C NMR (101 MHz, DMSO-d_6) δ (ppm): 164.33, 164.11, 156.49, 146.64, 133.76, 133.05, 131.39, 130.92, 130.16, 129.84, 129.00, 128.64, 128.15, 124.65, 123.85, 123.73, 123.05, 123.02, 122.18, 121.86, 112.46, 112.35, 63.70, 52.64, 37.39, 22.16. HRMS (ESI) m/z calcd for $C_{30}H_{27}N_2O_3^+$ $(M+H)^+$ 463.2022, found 463.2016.

Preparation of NBH

NBH was prepared according to the similar procedure to that of **NBOH**. A white solid was obtained (yield: 65%). ^{1}H NMR (400 MHz, DMSO-d_6) δ (ppm): 8.51 (dd, J = 13.2, 7.7 Hz, 2H), 8.29 (d, J = 7.7 Hz, 1H), 8.18 (d, J = 7.9 Hz, 1H), 7.96 (t, J = 7.6 Hz, 1H), 3.98 (t, J = 7.4 Hz, 2H), 1.65 (q, J = 7.6 Hz,

2H), 0.92 (t, J = 7.2 Hz, 3H). ^{13}C NMR (101 MHz, DMSO-d_6) δ (ppm): 163.35, 133.04, 132.03, 131.81, 131.42, 130.24, 129.55, 129.26, 128.74, 123.21, 122.43, 41.81, 21.25, 11.85.

Preparation of NDH

NDH was prepared according to the similar procedure to that of **NDOH**. A light green solid was obtained (yield: 76%). ^1H NMR (400 MHz, DMSO-d_6) δ (ppm): 8.63 – 8.54 (m, 2H), 8.40 (d, J = 1.9 Hz, 1H), 8.32 (dd, J = 8.5, 1.2 Hz, 1H), 8.28 – 8.21 (m, 1H), 7.92 (dd, J = 8.0, 2.6 Hz, 2H), 7.87 (dd, J = 8.5, 7.2 Hz, 1H), 7.79 (dt, J = 8.3, 0.9 Hz, 1H), 7.70 (dd, J = 8.5, 1.9 Hz, 1H), 7.64 – 7.55 (m, 1H), 7.45 (td, J = 7.5, 1.0 Hz, 1H), 4.11 – 4.02 (m, 2H), 1.78 – 1.64 (m, 2H), 0.96 (t, J = 7.4 Hz, 3H). ^{13}C NMR (101 MHz, DMSO-d_6) δ (ppm): 163.99, 163.78, 156.48, 155.87, 146.51, 132.92, 131.30, 130.86, 130.14, 129.84, 128.98, 128.60, 128.47, 128.09, 124.60, 123.83, 123.77, 123.10, 122.92, 122.15, 121.75, 112.40, 112.31, 41.73, 21.36, 11.87. HRMS (ESI) m/z calcd for $C_{27}H_{20}NO_3^+$ $(M+H)^+$ 406.1438, found 406.1438.

Preparation of PBOH

To a stirred solution of 4-bromophthalic acid anhydride (2.27 g, 10 mmol) in *N, N*-dimethylformamide (DMF, 50 mL) was added 3-aminopropanol (0.84 mL, 11 mmol). The resulting mixture was heated at 120 °C for 12 h with a Dean-Stark trap. After completion, the reaction mixture was cooled to room temperature and poured into saturated NaCl aqueous solution. After filtration the residue was purified by column chromatography. The crude product was then recrystallized from EtOH to give the pure product. White solid (yield 71%, 2.0 g). ^1H NMR (400 MHz, DMSO-d_6) δ (ppm): 8.08 – 7.97 (m, 2H), 7.83 – 7.74 (m, 1H), 4.52 (t, J = 5.0 Hz, 1H), 3.66 – 3.58 (m, 2H), 3.44 (td, J = 6.1, 5.0 Hz, 2H), 1.79 – 1.68 (m, 2H). ^{13}C NMR (101 MHz, DMSO-d_6) δ (ppm): 167.71, 167.14, 137.44, 134.22, 131.16, 128.30, 126.33, 125.28, 59.00, 35.91, 31.51. HRMS (ESI) m/z calcd for $C_{11}H_{11}BrNO_3^+$ $(M+H)^+$ 283.9917, found 283.9911.

Preparation of POOH

POOH was prepared according to the similar procedure to that of **PBOH**. A white solid was obtained (yield: 32%). ^{1}H NMR (400 MHz, DMSO-d_6) δ (ppm): 7.78 (dd, J = 8.3, 1.2 Hz, 1H), 7.38 (t, J = 1.9 Hz, 1H), 7.35 – 7.26 (m, 1H), 4.52 (t, J = 5.0 Hz, 1H), 3.92 (s, 3H), 3.60 (dd, J = 8.0, 6.5 Hz, 2H), 3.43 (td, J = 6.2, 5.0 Hz, 2H), 1.78 – 1.67 (m, 2H). ^{13}C NMR (101 MHz, DMSO-d_6) δ (ppm): 168.11, 168.07, 164.76, 134.77, 125.30, 123.92, 120.03, 108.68, 59.01, 56.72, 35.60, 31.71. HRMS (ESI) m/z calcd for $C_{12}H_{14}NO_4^+$ $(M+H)^+$ 236.0923, found 236.0917.

Preparation of PMOH

PMOH was prepared according to the similar procedure to that of **PBOH**. A white solid was obtained (yield: 78%). ^{1}H NMR (400 MHz, DMSO-d_6) δ (ppm): 7.75 (d, J = 7.6 Hz, 1H), 7.69 (s, 1H), 7.66 – 7.60 (m, 1H), 4.52 (t, J = 5.1 Hz, 1H), 3.65 – 3.57 (m, 2H), 3.43 (td, J = 6.2, 5.0 Hz, 2H), 2.48 (s, 3H), 1.78 – 1.67 (m, 2H). ^{13}C NMR (101 MHz, DMSO-d_6) δ (ppm): 168.50, 168.41, 145.70, 135.10, 132.49, 129.51, 123.87, 123.34, 59.02, 35.57, 31.67, 21.81. HRMS (ESI) m/z calcd for $C_{12}H_{14}NO_3^+$ $(M+H)^+$ 220.0974, found 220.0968.

Preparation of PHOH

PHOH was prepared according to the similar procedure to that of **PBOH**. A white solid was obtained (yield: 82%). ^{1}H NMR (400 MHz, DMSO-d_6) δ (ppm): 7.90 – 7.78 (m, 4H), 4.53 (t, J = 5.0 Hz, 1H), 3.67 – 3.57 (m, 2H), 3.44 (td, J = 6.1, 5.0 Hz, 2H), 1.79 – 1.68 (m, 2H). ^{13}C NMR (101 MHz, DMSO-d_6) δ (ppm): 168.42, 134.78, 132.11, 123.40, 59.03, 35.64, 31.62. HRMS (ESI) m/z calcd for $C_{11}H_{12}NO_3^+$ $(M+H)^+$ 206.0817, found 206.0812.

Preparation of PDOH

To a 100 ml Schlenk tube containing **PBOH** (1.42 g, 5 mmol) and **DBF-Bpin** (1.76 g, 6 mmol), K_2CO_3 (1.38 g, 10 mmol), $PdCl_2$ (0.04 g, 0.25 mmol), 15 mL of DMF and 3 mL of deionized water were added

under nitrogen atmosphere. The solution was heated at 80 °C for 24 h and then poured into water. The mixture was extracted with EA for several times and washed with brine. After dried over anhydrous sodium sulfate, the solvent was removed in vacuo, and the pure product was isolated by column chromatography on silica gel. The crude product was then recrystallized from ethanol to give PDOH (1.0 g, 52%) as a light green solid. ^{1}H NMR (400 MHz, DMSO-d_6) δ (ppm): 8.73 (d, J = 2.0 Hz, 1H), 8.32 – 8.21 (m, 3H), 8.04 – 7.94 (m, 2H), 7.84 (dd, J = 8.5, 3.6 Hz, 1H), 7.79 – 7.71 (m, 1H), 7.62 – 7.53 (m, 1H), 7.51 – 7.42 (m, 1H), 4.56 (t, J = 5.0 Hz, 1H), 3.72 – 3.64 (m, 2H), 3.48 (td, J = 6.2, 5.0 Hz, 2H), 1.84 – 1.72 (m, 2H). ^{13}C NMR (101 MHz, DMSO-d_6) δ (ppm): 168.30, 168.25, 156.46, 156.20, 146.51, 134.00, 133.24, 132.98, 130.46, 128.53, 127.38, 124.99, 124.06, 123.89, 123.84, 122.08, 121.60, 120.73, 112.73, 112.26, 59.06, 35.75, 31.67. HRMS (ESI) m/z calcd for $C_{23}H_{18}NO_4^+$ $(M+H)^+$ 372.1230, found 372.1220.

Preparation of PPOH

PPOH was prepared according to the similar procedure to that of **PDOH**. A white solid was obtained (yield: 26%).^{1}H NMR (400 MHz, DMSO-d_6) δ (ppm): 8.15 – 8.07 (m, 2H), 7.93 (dd, J = 7.6, 0.8 Hz, 1H), 7.85 – 7.78 (m, 2H), 7.58 – 7.50 (m, 2H), 7.50 – 7.43 (m, 1H), 4.55 (t, J = 5.0 Hz, 1H), 3.70 – 3.56 (m, 2H), 3.46 (td, J = 6.2, 5.0 Hz, 2H), 1.82 – 1.70 (m, 2H). ^{13}C NMR (101 MHz, DMSO-d_6) δ (ppm): 168.26, 168.24, 146.62, 138.73, 133.21, 132.95, 130.79, 129.73, 129.36, 127.72, 124.10, 121.39, 59.04, 35.73, 31.62. HRMS (ESI) m/z calcd for $C_{17}H_{16}NO_3^+$ $(M+H)^+$ 282.1130, found 282.1125.

Preparation of PEOH

To a 100 ml Schlenk tube containing **PBOH** (1.42 g, 5 mmol), trimethylsilylacetylene (0.85 mL, 6 mmol), CuI (0.1 g, 0.5 mmol), $Pd(PPh_3)_2Cl_2$ (0.10 g, 0.15 mmol) and PPh_3 (0.04 g, 0.15 mmol), 15 mL of THF and 7.5 mL of triethylamine were added under nitrogen atmosphere. The solution was stirred at 60 °C for 8 h and then cooled to room temperature. After filtration the filtrate was then evaporated

under vacuum and purified by column chromatography to give **PEOH** (0.9 g, 62%) as a light green solid. ^{1}H NMR (400 MHz, DMSO-d_6) δ (ppm): 7.88 – 7.85 (m, 1H), 7.85 – 7.82 (m, 2H), 4.53 (t, J = 5.0 Hz, 1H), 3.67 – 3.59 (m, 2H), 3.44 (td, J = 6.2, 5.0 Hz, 2H), 1.79 – 1.68 (m, 2H), 0.27 (s, 9H). ^{13}C NMR (101 MHz, DMSO-d_6) δ (ppm): 167.58, 167.43, 137.65, 132.57, 131.60, 128.07, 125.79, 123.65, 103.66, 99.30, 58.89, 35.73, 31.41, 0.00. HRMS (ESI) m/z calcd for $C_{16}H_{20}NO_3Si^+$ $(M+H)^+$ 302.1207, found 302.1204.

Preparation of PBH

PBH was prepared according to the similar procedure to that of **PBOH**. A white solid was obtained (yield: 86%). ^{1}H NMR (400 MHz, DMSO-d_6) δ (ppm): 8.08 – 7.99 (m, 2H), 7.80 (dd, J = 7.9, 0.6 Hz, 1H), 3.52 (dd, J = 7.7, 6.5 Hz, 2H), 1.67 – 1.53 (m, 2H), 0.86 (t, J = 7.5 Hz, 3H). ^{13}C NMR (101 MHz, DMSO-d_6) δ (ppm): 167.75, 167.18, 137.50, 134.10, 131.04, 128.37, 126.38, 125.32, 21.72, 11.66. MS (ESI) m/z calcd for $C_{11}H_{11}BrNO_2^+$ $(M+H)^+$ 268.0, found 268.0.

Preparation of PBB

PBOH (2.84 g, 10 mmol, dissolved in a minimal amount of $CHCl_3$) was added LiBr (1.80 g, 20 mmol) in 48 wt% aqueous HBr (10 mL) at 0 °C. The reaction mixture was allowed to warm to room temperature and reflux for overnight. The reaction mixture was diluted with ethyl acetate, washed with water and saturated $NaHCO_3$. The organic layer was collected, washed with brine, dried over $MgSO_4$, and concentrated. The residue was purified by column chromatography to afford **PBB** (1.9 g, 56%) as white solid. ^{1}H NMR (400 MHz, DMSO-d_6) δ (ppm): 8.10 – 7.99 (m, 2H), 7.80 (dd, 1H), 3.69 (t, J = 6.8 Hz, 2H), 3.56 (t, J = 6.5 Hz, 2H), 2.19 – 2.08 (m, 2H). ^{13}C NMR (101 MHz, DMSO-d_6) δ (ppm): 167.75, 167.19, 137.42, 134.31, 131.24, 128.28, 126.36, 125.31, 36.86, 32.36, 31.48. HRMS (ESI) m/z calcd for $C_{11}H_{10}Br_2NO_2^+$ $(M+H)^+$ 345.9073, found 345.9071.

Preparation of PBNC

PBB (1.73 g, 5 mmol), trimethylamine (2 M in THF, 12.5 mL) and THF (10 mL) were stirred at 55 ^{o}C for 24 h. The solvent was removed in vacuo, and the pure product was isolated by column chromatography on silica gel. The crude product is recrystallized from methanol to get **PBNC** as a white solid (1.3 g, 66%). ^{1}H NMR (400 MHz, DMSO-d_6) δ (ppm): 8.13 – 8.03 (m, 2H), 7.84 (dd, *J* = 7.9, 0.6 Hz, 1H), 3.65 (t, *J* = 6.2 Hz, 2H), 3.42 – 3.36 (m, 2H), 3.03 (s, 9H), 2.11 – 1.99 (m, 2H). ^{13}C NMR (101 MHz, DMSO-d_6) δ(ppm): 167.78, 167.22, 137.55, 134.28, 131.19, 128.43, 126.45, 125.44, 63.37, 52.69, 35.23, 22.36. HRMS (ESI) m/z calcd for $C_{14}H_{18}BrN_2O_2^+$ $(M+H)^+$ 325.0552, found 325.0546.

Preparation of NPOH

NPOH was prepared according to the similar procedure to that of **NDOH**. A yellow solid was obtained (yield: 49%).^{1}H NMR (400 MHz, DMSO-d_6) δ (ppm): 8.71 (d, *J* = 7.4 Hz, 1H), 8.55 (dd, *J* = 5.9, 2.3 Hz, 1H), 8.51 (d, *J* = 7.8 Hz, 1H), 8.42 (dd, *J* = 7.7, 1.1 Hz, 1H), 8.37 – 8.29 (m, 3H), 8.17 – 8.07 (m, 3H), 8.02 (d, *J* = 7.3 Hz, 1H), 7.74 – 7.67 (m, 2H), 7.52 (d, *J* = 9.2 Hz, 1H), 4.60 (t, *J* = 5.2 Hz, 1H), 4.23 – 4.18 (m, 2H), 3.57 (q, *J* = 6.2 Hz, 2H), 1.88 (dq, *J* = 8.6, 6.4 Hz, 2H). ^{13}C NMR (101 MHz, $CDCl_3$) δ (ppm): 165.06, 164.93, 146.46, 133.54, 133.21, 131.73, 131.61, 131.51, 131.41, 131.15, 130.77, 129.56, 128.53, 128.31, 128.28, 127.88, 127.34, 127.10, 126.46, 125.85, 125.54, 124.75, 124.58, 122.53, 121.85, 58.90, 36.86, 31.09. HRMS (ESI) m/z calcd for $C_{31}H_{22}NO_3^+$ $(M+H)^+$ 456.1594, found 456.1593.

II. Results and Discussion

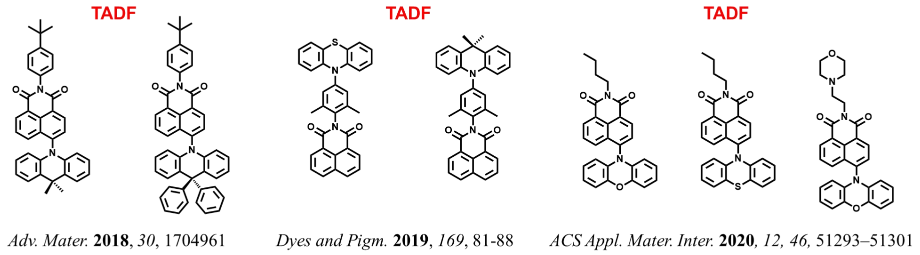

Fig. S1. Molecular structures of previously reported 1,8-naphthalimide derivatives[1-3] with thermally activated delayed fluorescence (TADF).

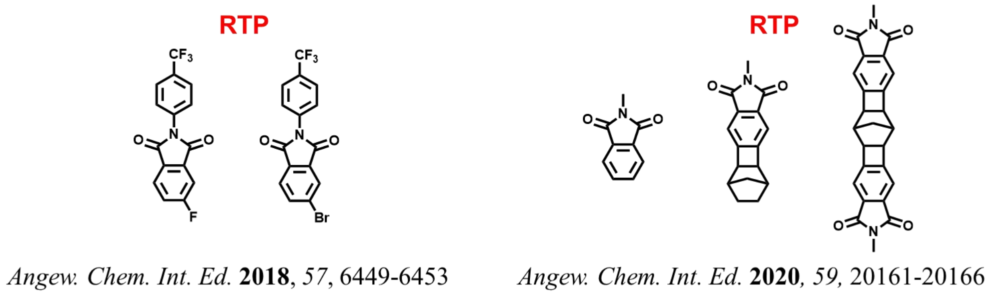

Fig. S2. Molecular structures of previously reported phthalimide derivatives[4-5] with room-temperature phosphorescence (RTP).

Photophysical properties of **NDOH** were first investigated. In dilute tetrahydrofuran (THF) solution (10^{-5} M), **NDOH** showed cyan fluorescence without phosphorescence at room temperature. However, orange phosphorescence could be observed at 77 K (Fig. S3), suggesting that radiative transitions of triplet excitons could be active in a rigid matrix.

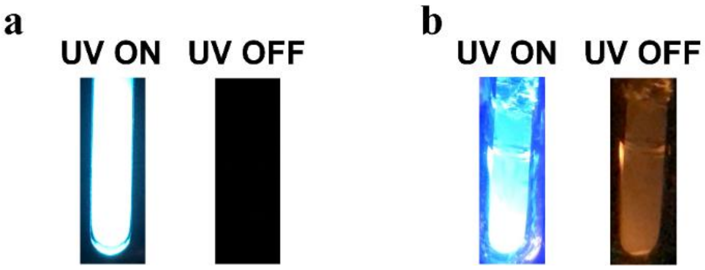

Fig. S3. Photographs of **NDOH** solution (50 μM) under UV light and after the removal of UV light at room temperature (a) and 77 K (b).

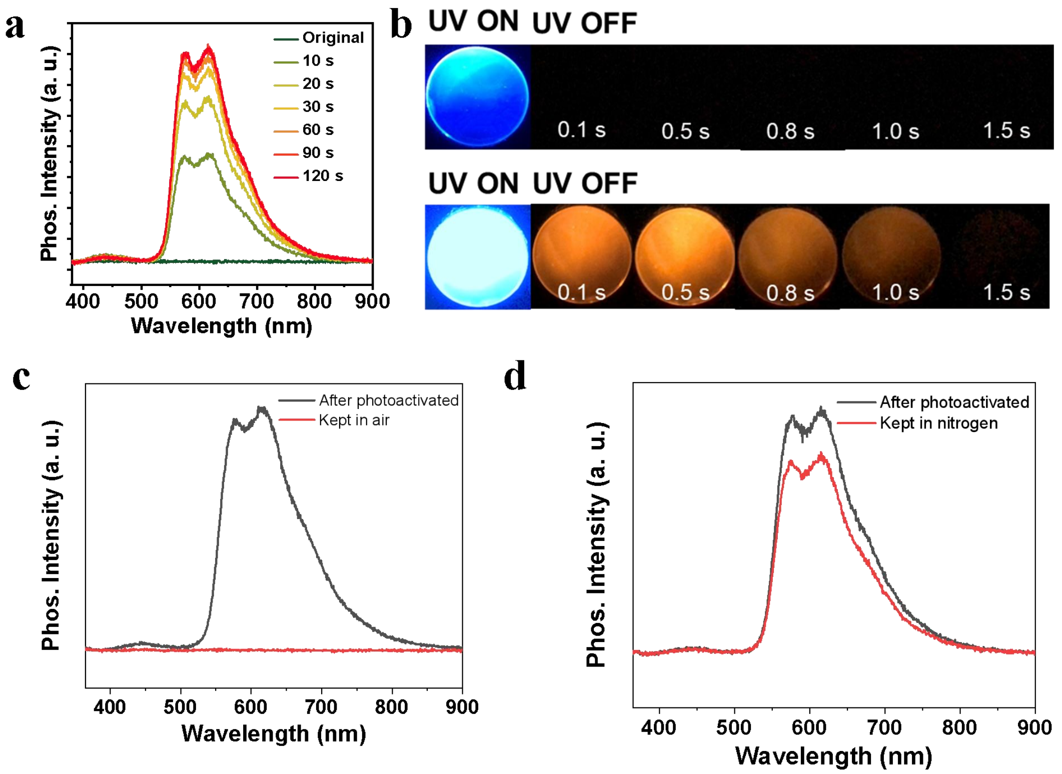

Fig. S4. a) Delayed spectra of **NDOH** doped in polymethyl methacrylate (PMMA) upon different UV irradiation time. λ_{ex}=365 nm. Inset: The chemical structure of **NDOH**. b) Photographs of **NDOH** doped in PMMA before (upper) and after (lower) 365 nm UV irradiation. c) Delayed spectra of NDOH doped in polymethyl methacrylate (PMMA) after photoactivation and kept in air. λ_{ex}=365 nm. d) Delayed spectra of **NDOH** doped in PMMA after photoactivation and kept in nitrogen. λ_{ex}=365 nm.

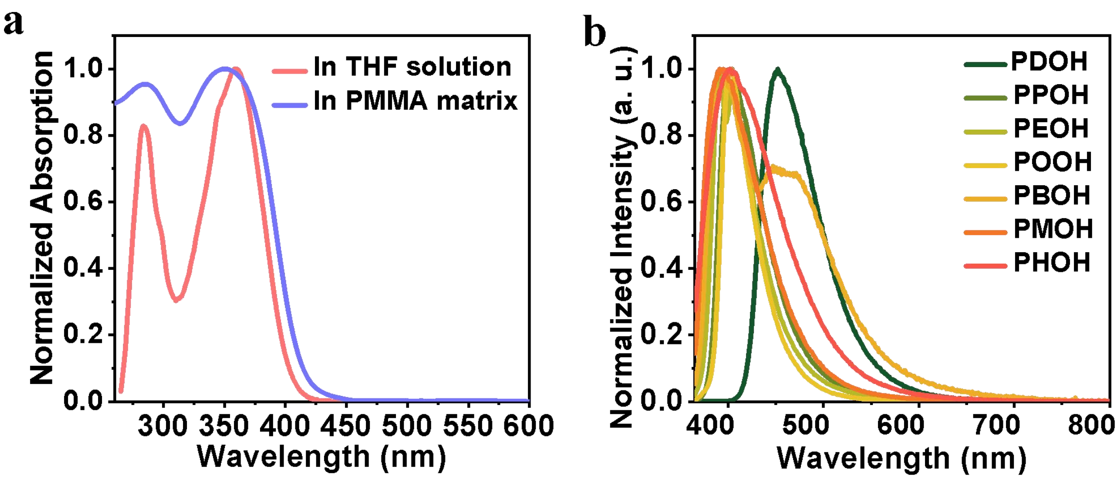

Fig. S5. a) UV-Vis absorption spectra of **NDOH** in tetrahydrofuran (THF) solution and doped in PMMA matrix. b) Normalized photoluminescence spectra of the host molecules. λ_{ex}=365 nm.

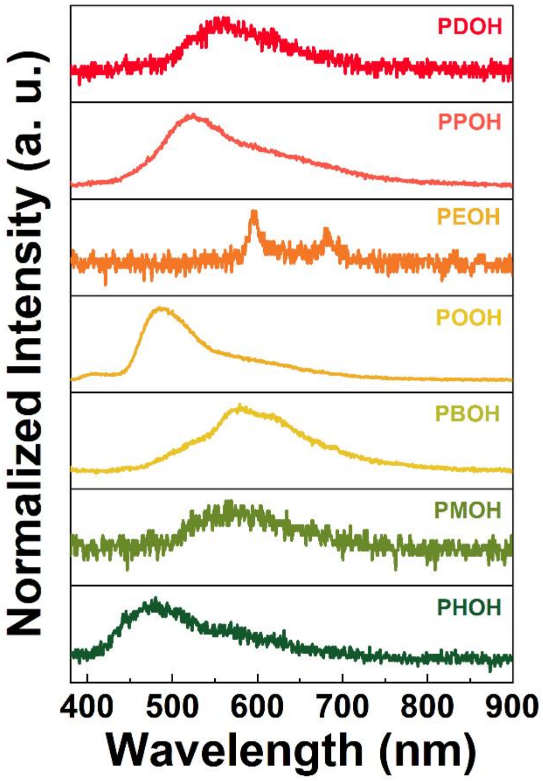

Fig. S6. Delayed spectra of **PDOH**, **PPOH**, **PEOH**, **POOH**, **PBOH**, **PMOH** and **PHOH** crystalline samples (λ_{ex}=365 nm, delayed time = 8 ms).

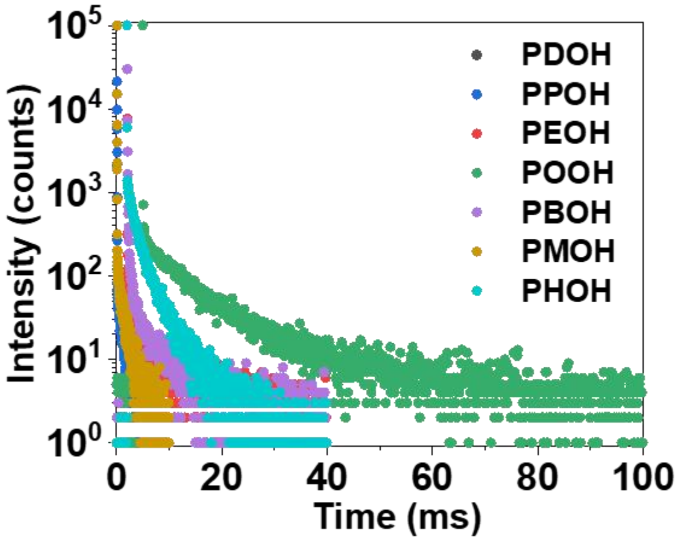

Fig. S7. Decay curves of **PDOH**, **PPOH**, **PEOH**, **POOH**, **PBOH**, **PMOH** and **PHOH** crystalline samples monitored at maximum emission wavelengths of delayed spectra.

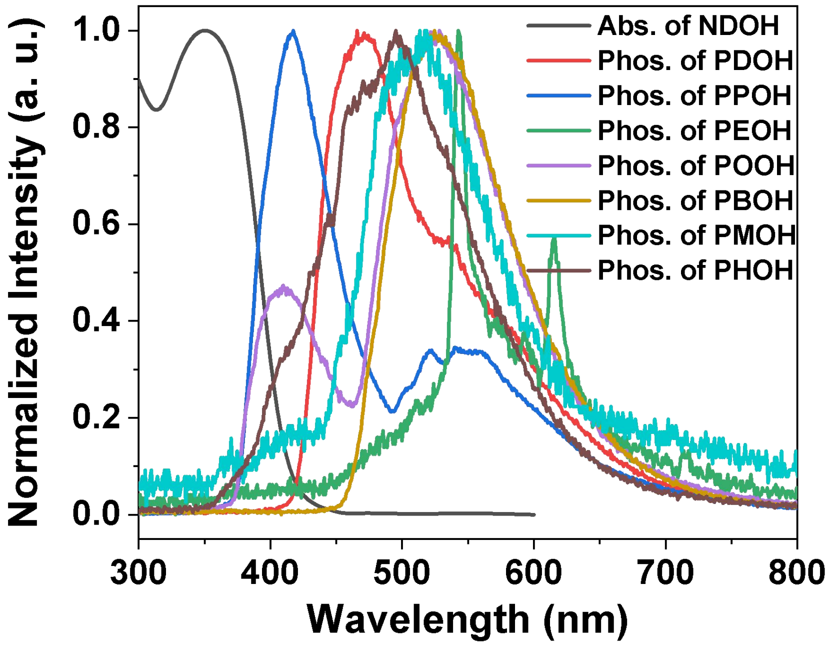

Fig. S8. The spectral overlap between the phosphorescence emission of the host materials and the absorption spectrum of **NDOH**.

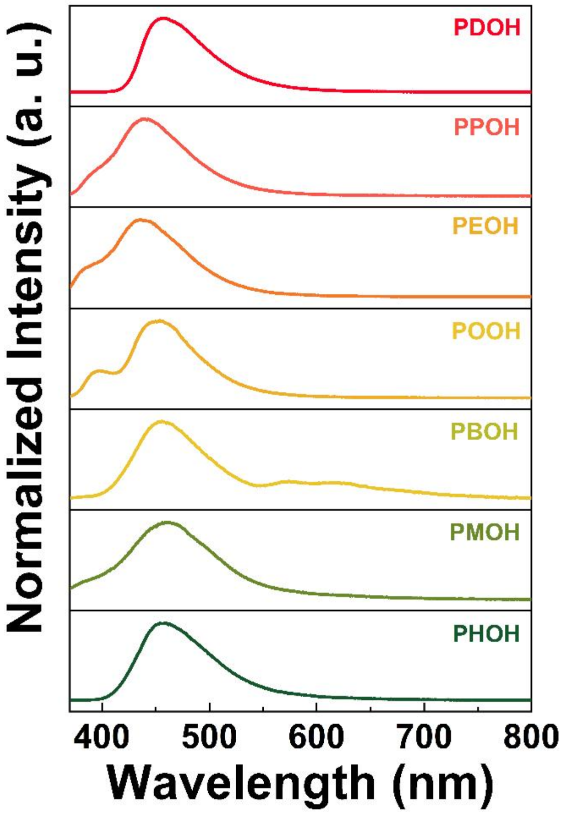

Fig. S9. Photoluminescence spectra of **NDOH** doped in different host materials (from top to bottom: **PDOH**, **PPOH**, **PEOH**, **POOH**, **PBOH**, **PMOH** and **PHOH**.

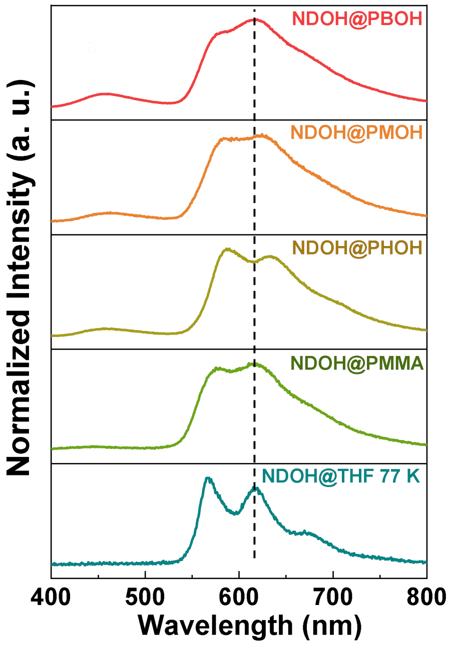

Fig. S10. The delayed emission spectra of **NDOH@PBOH**, **NDOH@PMOH**, **NDOH@PHOH** and photoactivated **NDOH**@PMMA film at room temperature, and **NDOH** solution in THF at 77 K. The delay time is 8 ms.

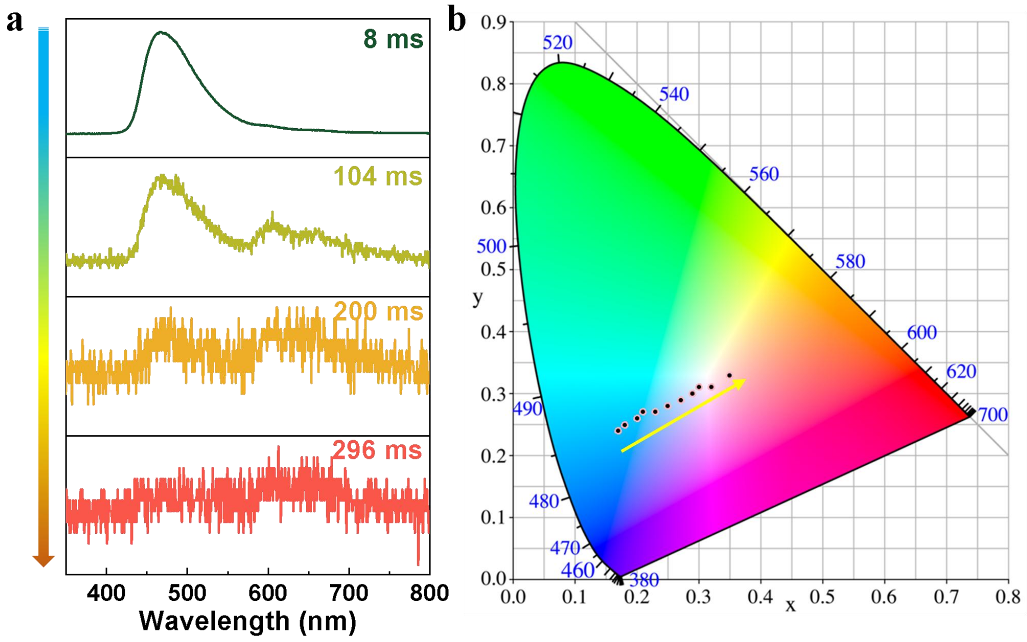

Fig. S11. a) Delayed spectra of **NDOH@PDOH** powder at different delay times. b) Chromatic CIE coordinates of **NDOH@PDOH** powder at different delay times based on the delayed spectra.

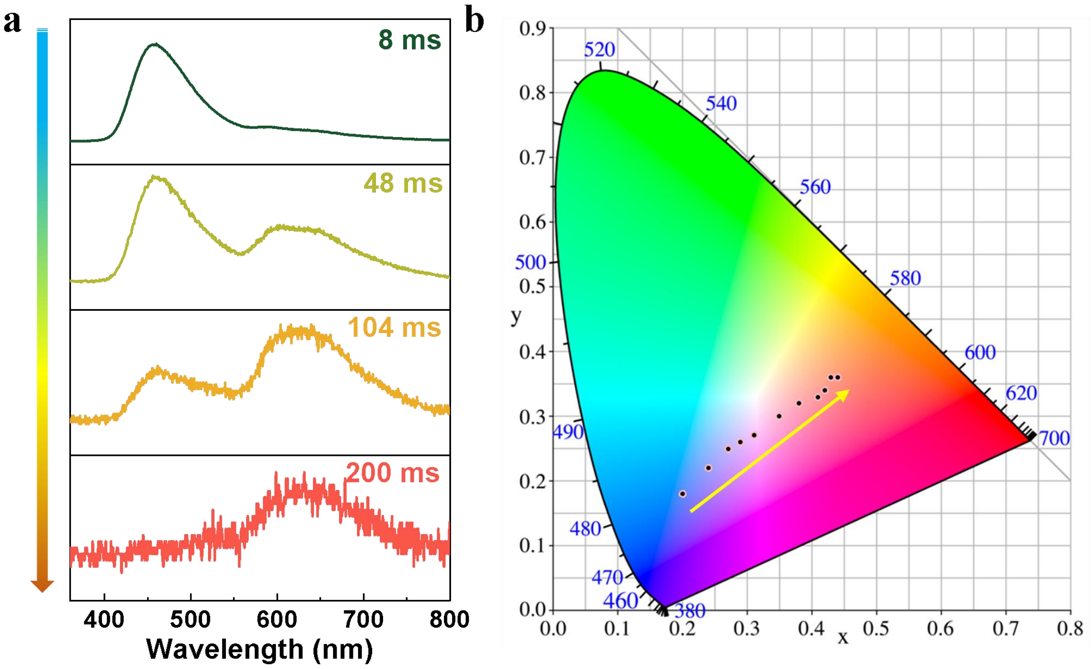

Fig. S12. a) Delayed spectra of **NDOH@PPOH** powder at different delay times. b) Chromatic CIE coordinates of **NDOH@PPOH** powder at different delay times based on the delayed spectra.

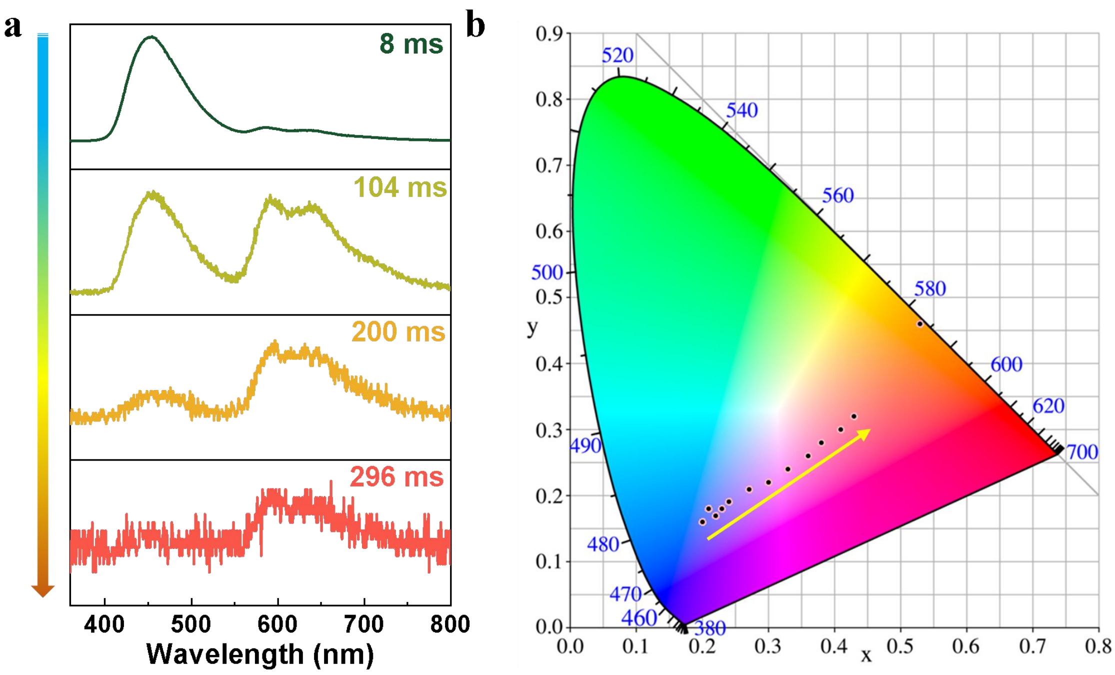

Fig. S13. a) Delayed spectra of **NDOH@PEOH** powder at different delay times. b) Chromatic CIE coordinates of **NDOH@PEOH** powder at different delay times based on the delayed spectra.

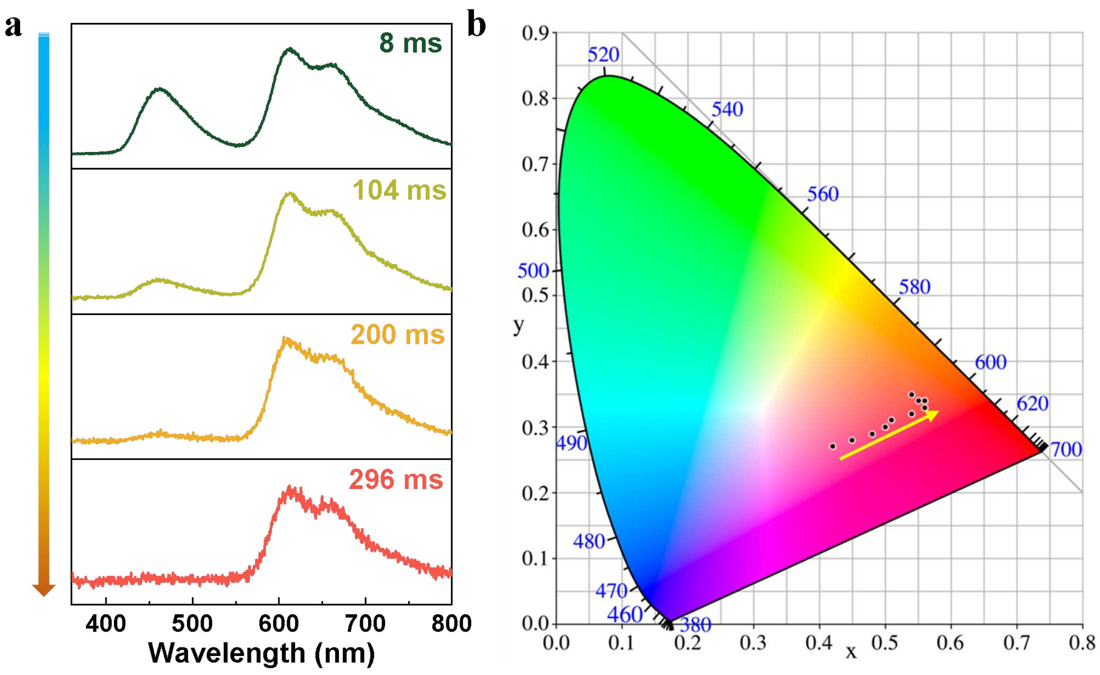

Fig. S14. a) Delayed spectra of **NDOH@POOH** powder at different delay times. b) Chromatic CIE coordinates of **NDOH@POOH** powder at different delay times based on the delayed spectra.

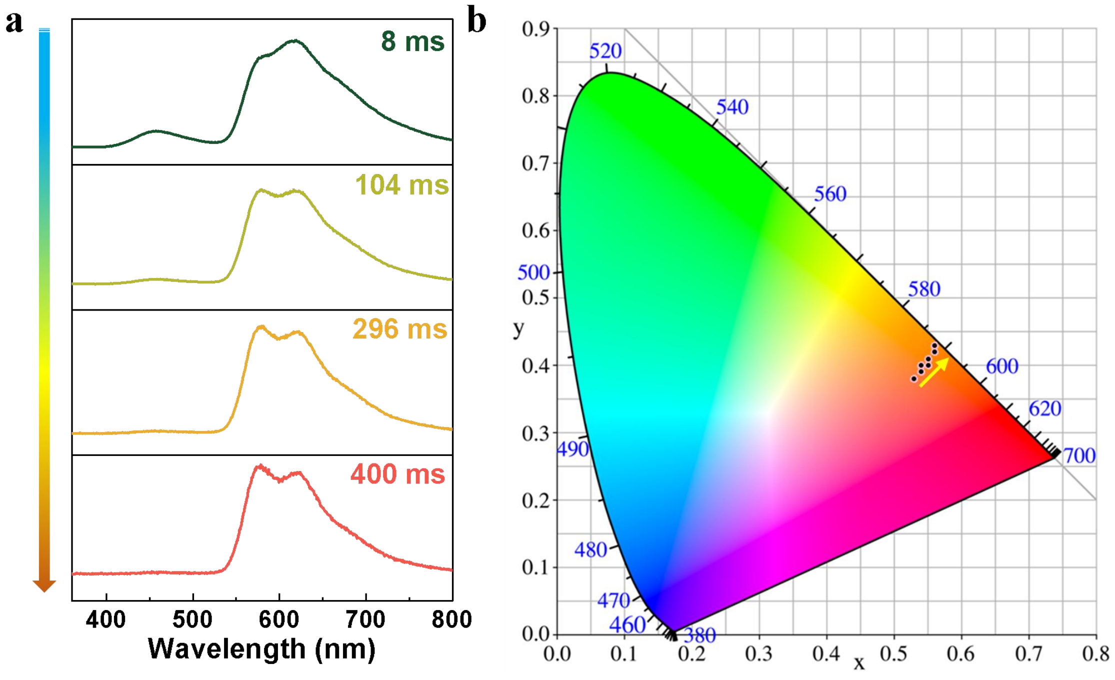

Fig. S15. a) Delayed spectra of **NDOH@PBOH** powder at different delay times. b) Chromatic CIE coordinates of **NDOH@PBOH** powder at different delay times based on the delayed spectra.

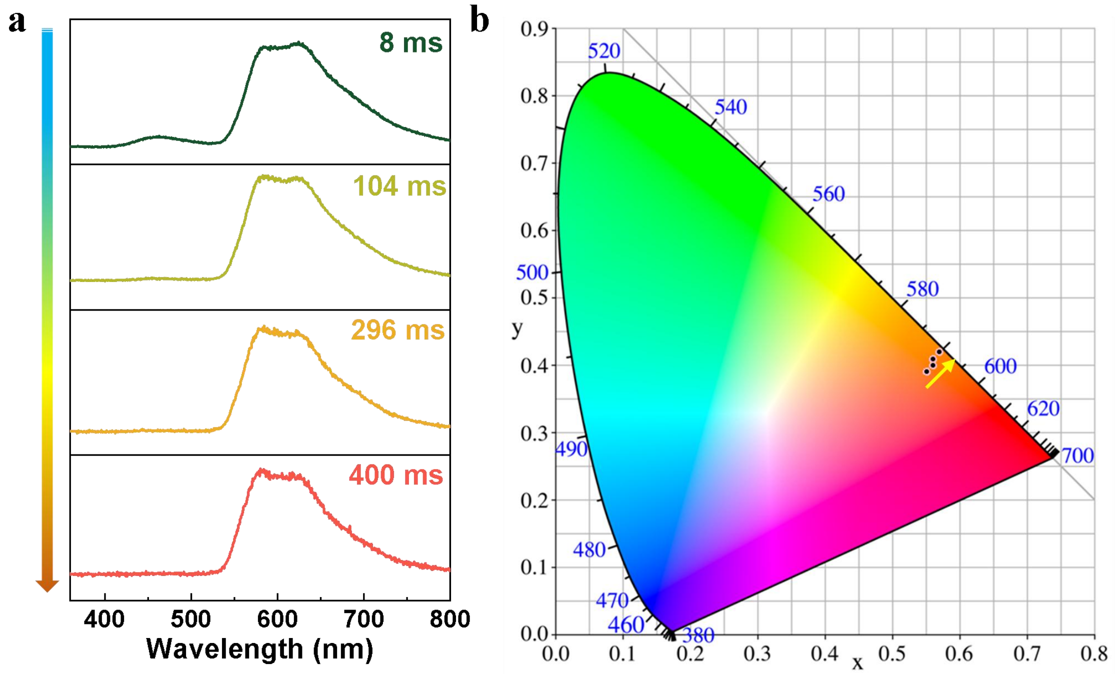

Fig. S16. a) Delayed spectra of **NDOH@PMOH** powder at different delay times. b) Chromatic CIE coordinates of **NDOH@PMOH** powder at different delay times based on the delayed spectra.

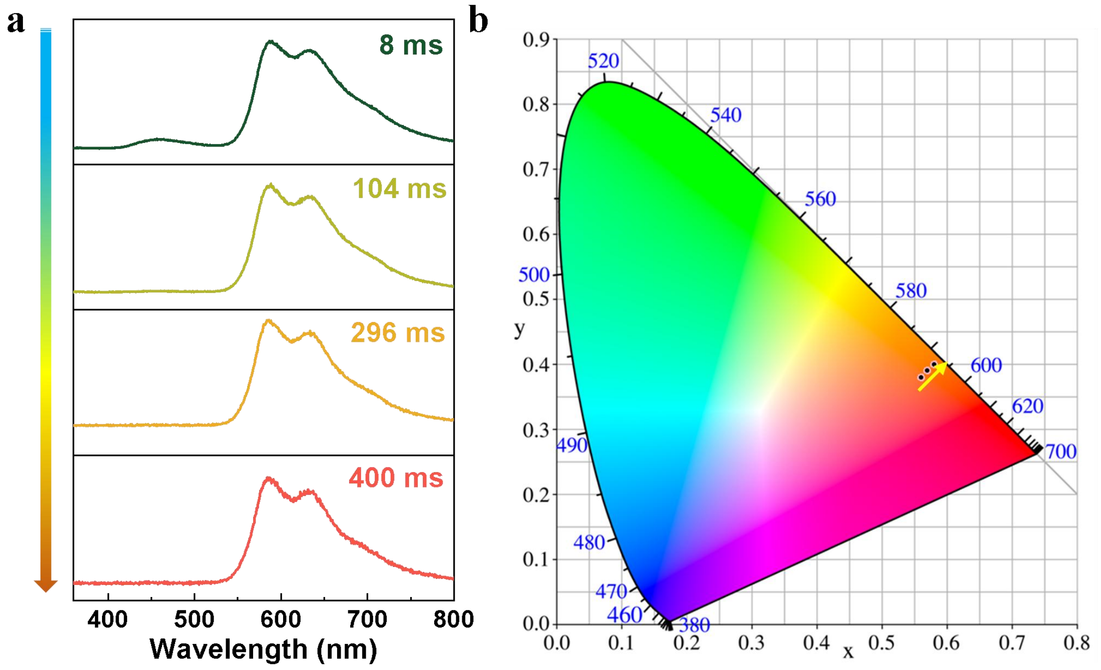

Fig. S17. a) Delayed spectra of **NDOH@PHOH** powder at different delay times. b) Chromatic CIE coordinates of **NDOH@PHOH** powder at different delay times based on the delayed spectra.

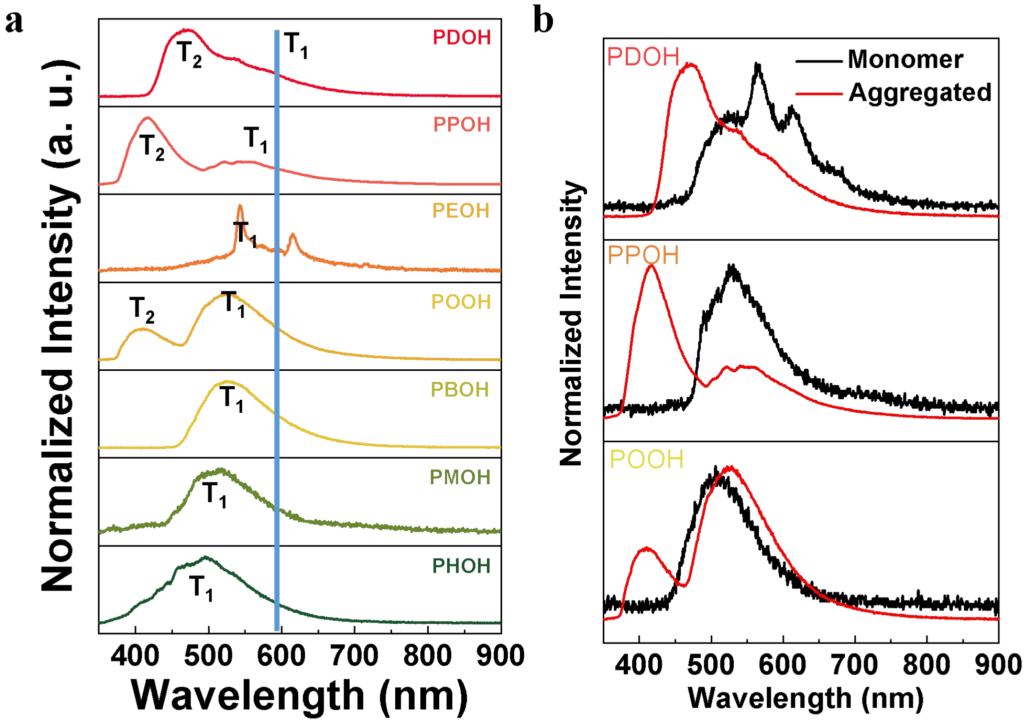

Fig. S18. a) Phosphorescence spectra of **PDOH**, **PPOH**, **PEOH**, **POOH**, **PBOH**, **PMOH** and **PHOH** crystalline samples at 77 K (λ_{ex}=365 nm, delayed time = 8 ms). b) Phosphorescence spectra of **PDOH**, **PPOH** and **POOH** in the monomer state (10^{-5} M THF solution) and aggregated state (crystalline samples) at 77 K.

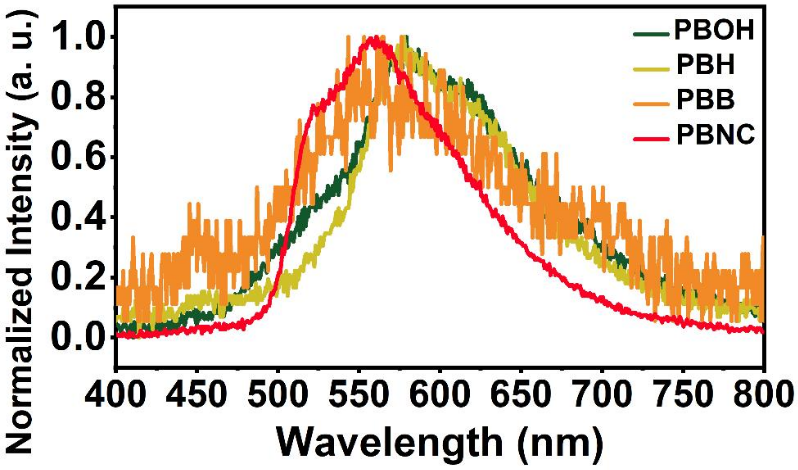

Fig. S19. Phosphorescence spectra of **PBOH**, **PBH**, **PBB** and **PBNC** crystalline samples at room temperature (λ_{ex}=365 nm, delayed time = 8 ms).

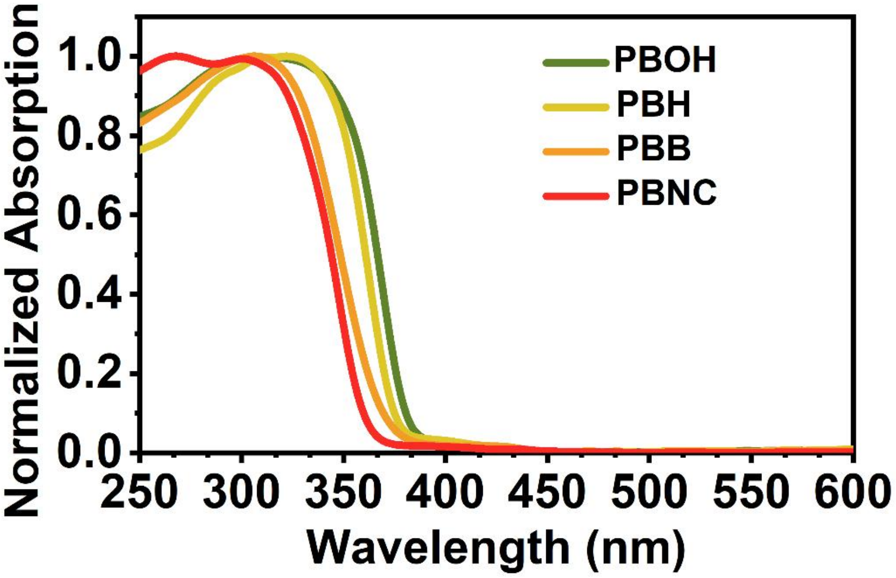

Fig. S20. Solid UV-vis diffuse reflection spectra of crystalline powder of **PBOH**, **PBH**, **PBB** and **PBNC**.

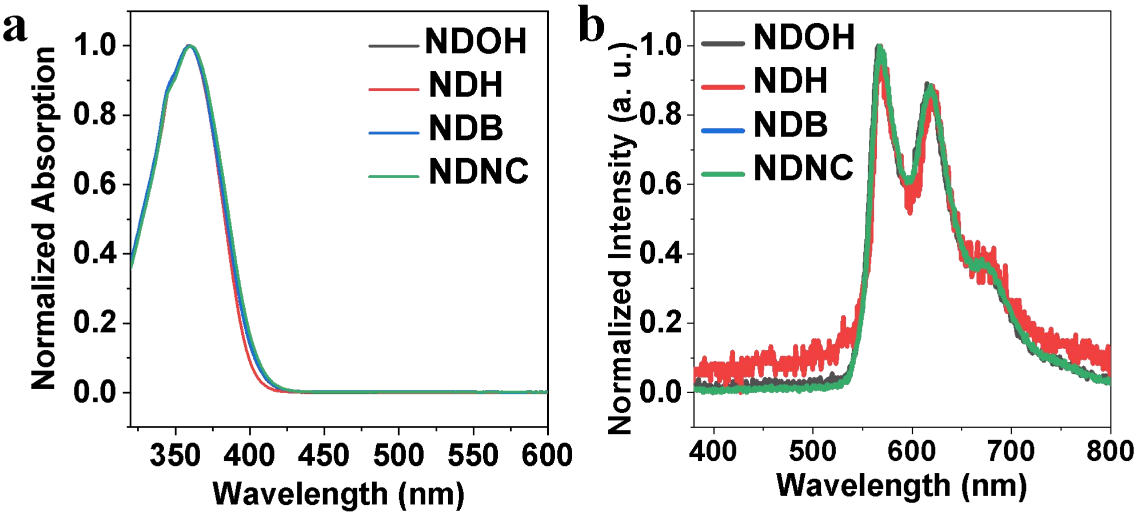

Fig. S21. a) UV-Vis absorption spectra of **NDOH**, **NDH**, **NDB** and **NDNC** in THF solution (50 μM). b) Phosphorescence spectra of **NDOH**, **NDH**, **NDB** and **NDNC** in THF solution (50 μM) at 77 K.

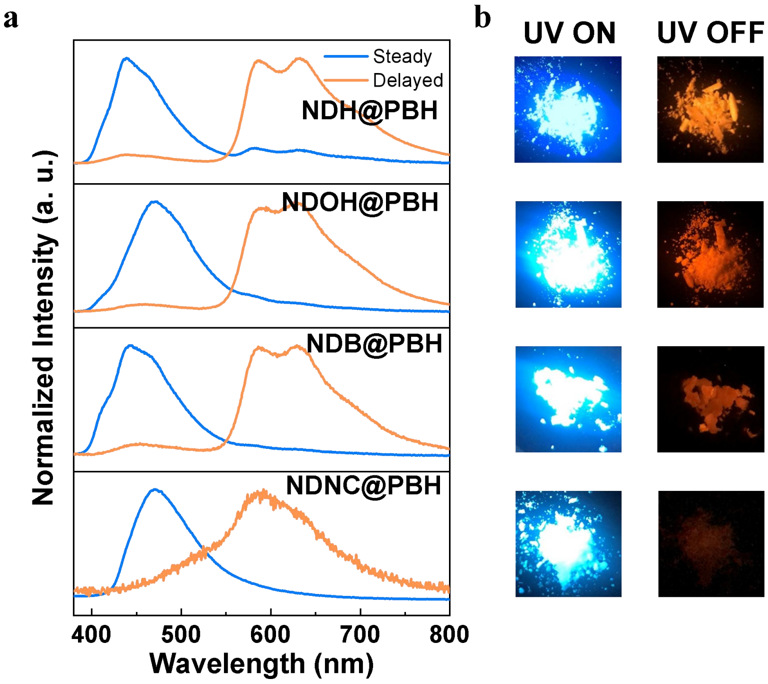

Fig. S22. a) Steady spectra and delayed spectra of **NDH@PBH**, **NDOH@PBH**, **NDB@PBH** and **NDNC@PBH** powder (λ_{ex}=365 nm, delayed time = 8 ms). b) The corresponding photographs taken under UV light and after the removal of UV light.

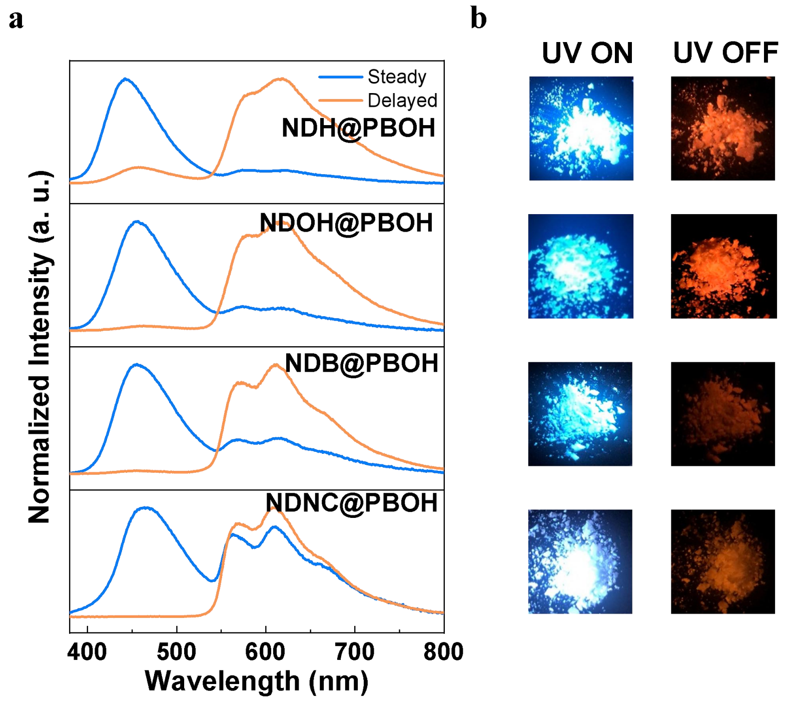

Fig. S23. a) Steady spectra and delayed spectra of **NDH@PBOH**, **NDOH@PBOH**, **NDB@PBOH** and **NDNC@PBOH** powder (λ_{ex}=365 nm, delayed time = 8 ms). b) The corresponding photographs taken under UV light and after the removal of UV light.

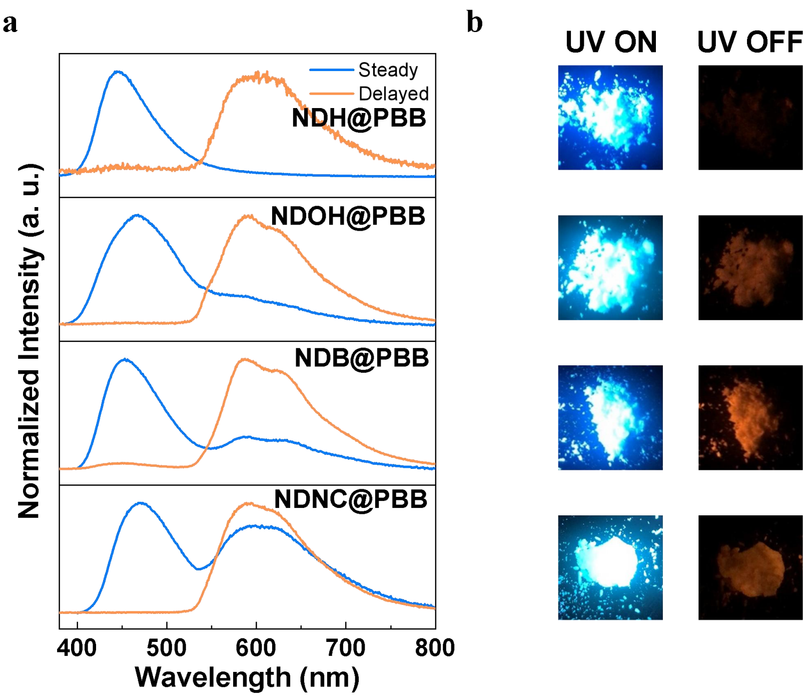

Fig. S24. a) Steady spectra and delayed spectra of **NDH@PBB**, **NDOH@PBB**, **NDB@PBB** and **NDNC@PBB** powder (λ_{ex}=365 nm, delayed time = 8 ms). b) The corresponding photographs taken under UV light and after the removal of UV light.

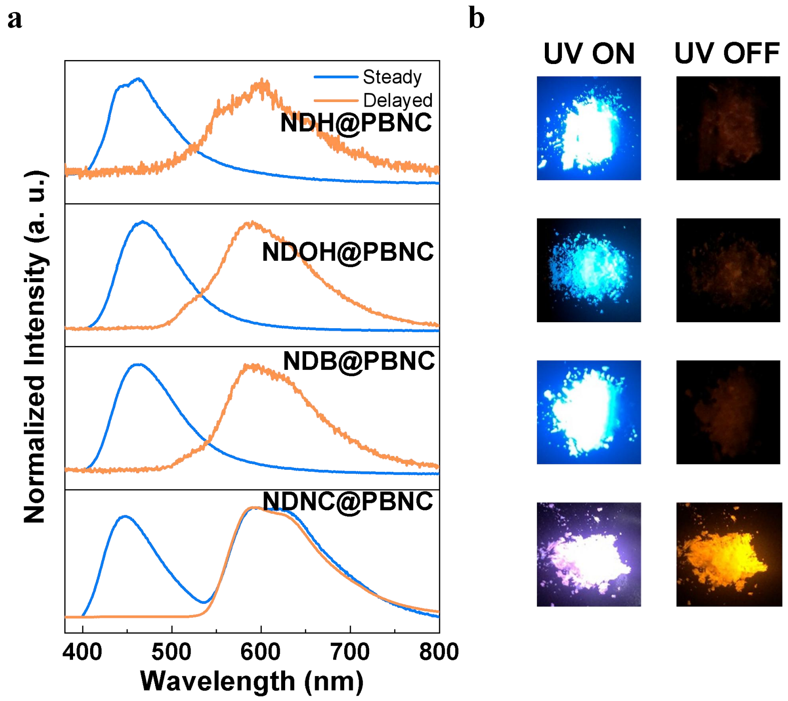

Fig. S25. a) Steady spectra and delayed spectra of **NDH@PBNC**, **NDOH@PBNC**, **NDB@PBNC** and **NDNC@PBNC** powder (λ_{ex}=365 nm, delayed time = 8 ms). b) The corresponding photographs taken under UV light and after the removal of UV light.

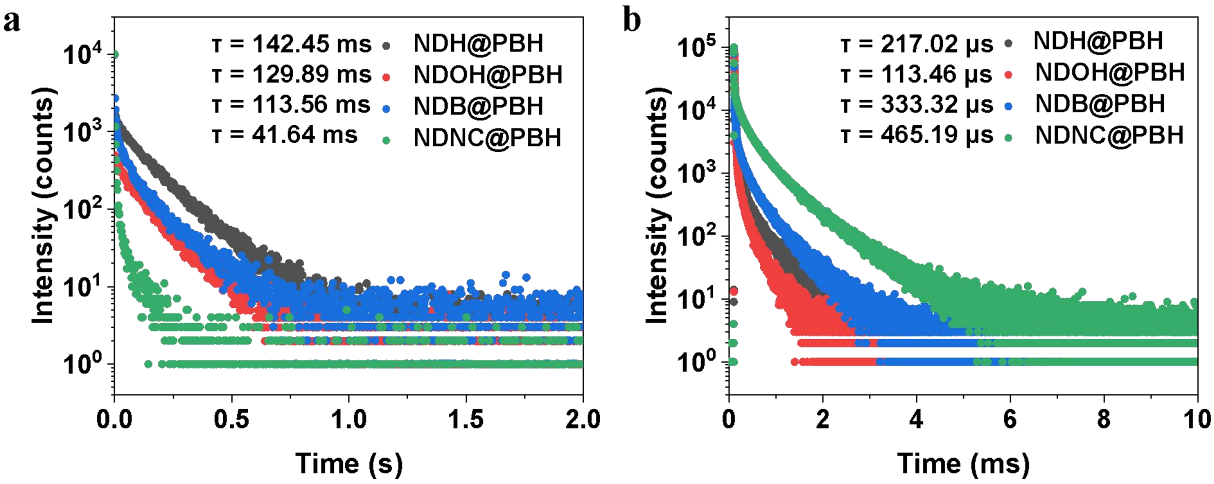

Fig. S26. Decay curves of **NDH@PBH**, **NDOH@PBH**, **NDB@PBH** and **NDNC@PBH** powder monitored at maximum emission wavelengths of afterglow spectra in long wavelength region (a) and short wavelength region (b).

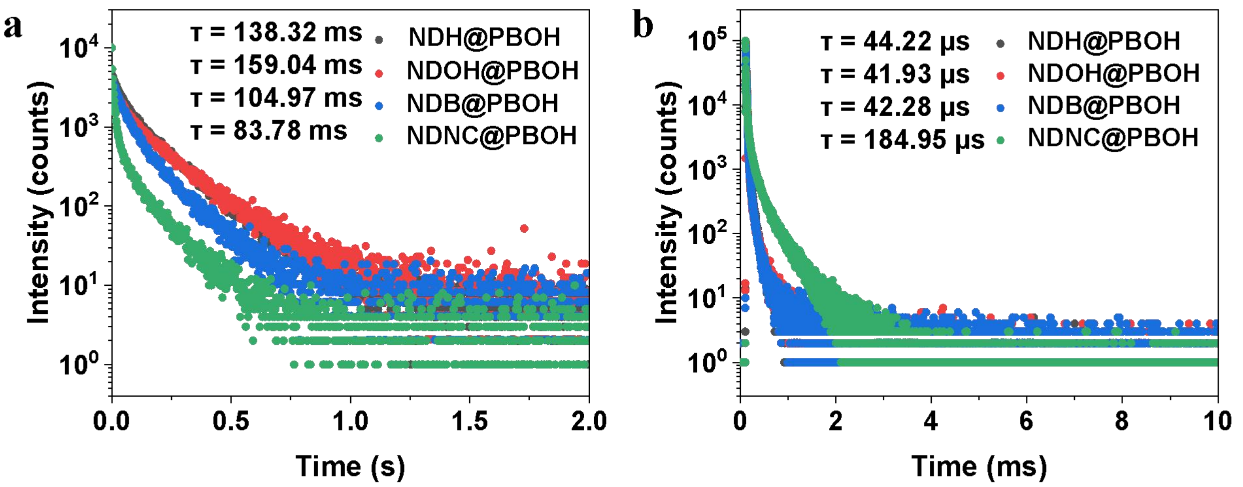

Fig. S27. Decay curves of **NDH@PBOH**, **NDOH@PBOH**, **NDB@PBOH** and **NDNC@PBOH** powder monitored at maximum emission wavelengths of afterglow spectra in long wavelength region (a) and short wavelength region (b).

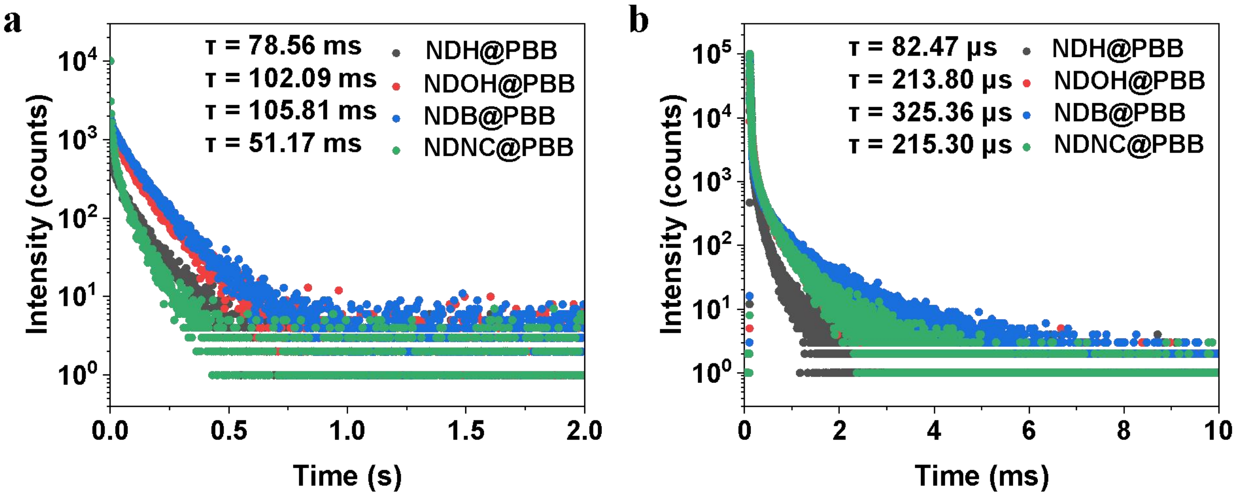

Fig. S28. Decay curves of **NDH@PBB**, **NDOH@PBB**, **NDB@PBB** and **NDNC@PBB** powder monitored at maximum emission wavelengths of afterglow spectra in long wavelength region (a) and short wavelength region (b).

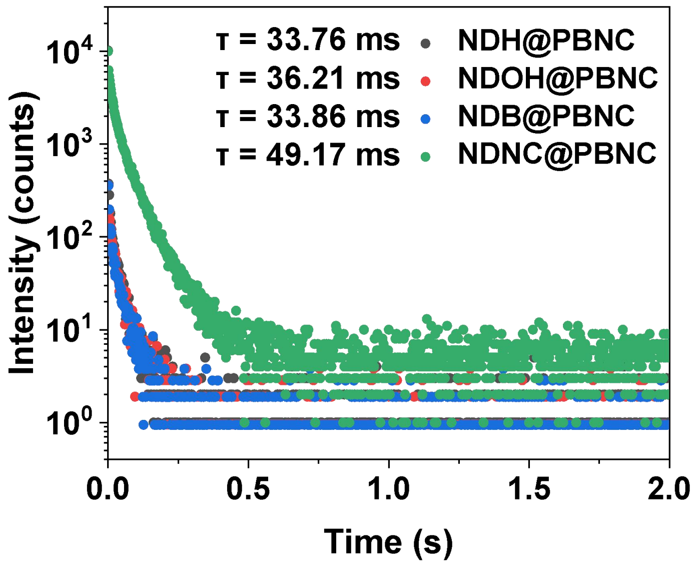

Fig. S29. Decay curves of **NDH@PBNC**, **NDOH@PBNC**, **NDB@PBNC** and **NDNC@PBNC** powder monitored at maximum emission wavelengths of afterglow spectra.

In general, nonradiative energy transfer can be classified as Förster resonance (coulombic) and Dexter exchange (collisional) energy transfer. The Coulomb Interaction Energy can be defined as:

$$V_{en}^{c} = <\psi_{D^*}(1)\psi_A(2)|V|\psi_D(1)\psi_{A^*}(2)>$$

where ψ_D and ψ_{D^*} represent the ground state and excited state wave functions of donor, respectively, and ψ_A and ψ_{A^*} represent those of acceptor. Since wave functions should contain space (ϕ) and spin (S) part, and spin functions exhibit orthogonality, only if $S_{D^*} = S_D$ and $S_A = S_{A^*}$, the Coulomb Interaction Energy will give a value different from zero. That is, spins of donor and acceptor before and after Förster energy transfer should be same. Thus, TTET can not occur after coulombic interaction, though TSET ($^3D^* + {}^1A \rightarrow {}^1D + {}^1A^*$) can be observed due to the long lifetime of $^3D^*$.

The absorption spectra and phosphorescence spectra of dilute solution (10^{-5} M) of **NDOH**, **NDH**, **NDB** and **NDNC** at 77 K were almost the same, indicating the similar electronic structures of the guest molecules (Fig. S19). Accordingly, each **NDOH**, **NDH**, **NDB** and **NDNC** was doped into **PBOH**, **PBH**,

PBB and **PBNC**. As demonstrated by the PL spectra, delayed spectra, phosphorescence decay curves and phosphorescence quantum yields (Fig. S20-S27), efficient TTET tended to occur between the host and guest materials with the same terminal groups. It should be ascribed to the theory that similarities are likely to be solvable in each other, which was most manifest when **PBNC** acted as host and the polarity of guest molecules **NDOH**, **NDH** and **NDB** was significantly different from that of **PBNC**, leading to their weak afterglow. The good compatibility helped to gain close distances between host and guest molecules, which was favorable to Dexter energy transfer.

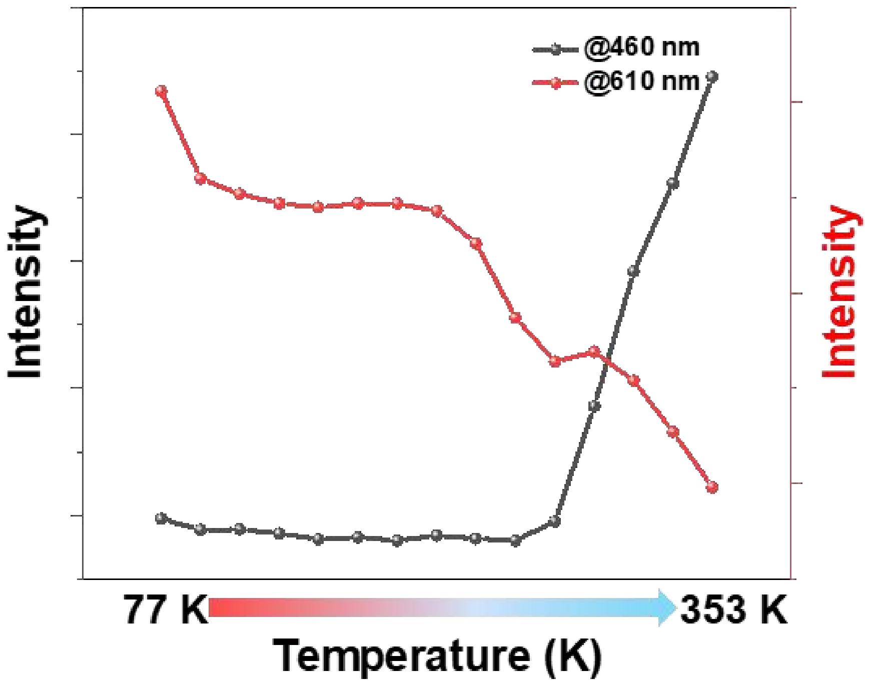

Fig. S30. The line chart displaying the afterglow intensities changes at 460 and 610 nm of **NDOH@POOH** powder with increasing temperature.

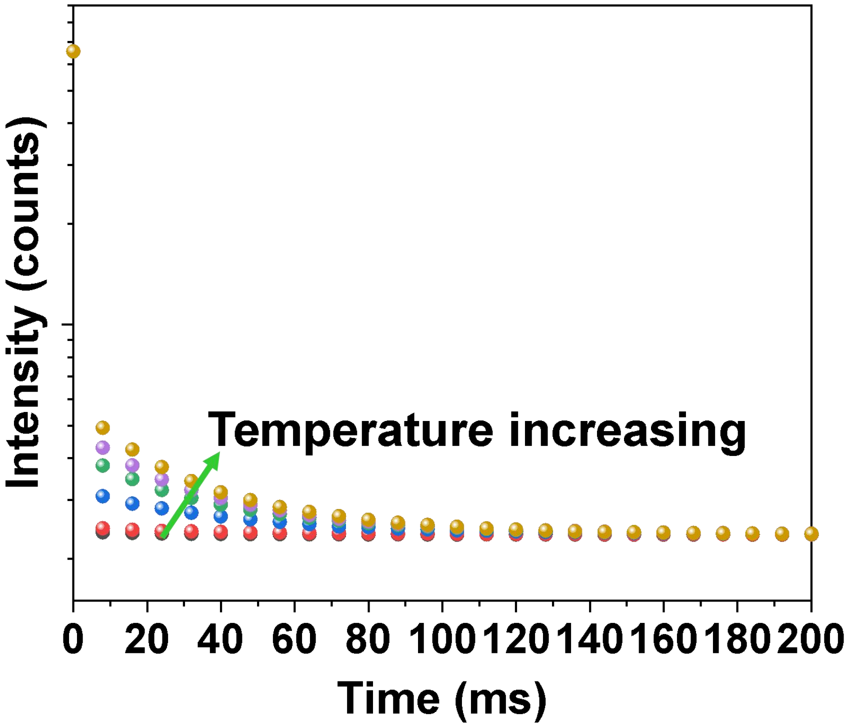

Fig. S31. Intensity decay curves at 460 nm of **NDOH@POOH** powder at different temperatures.

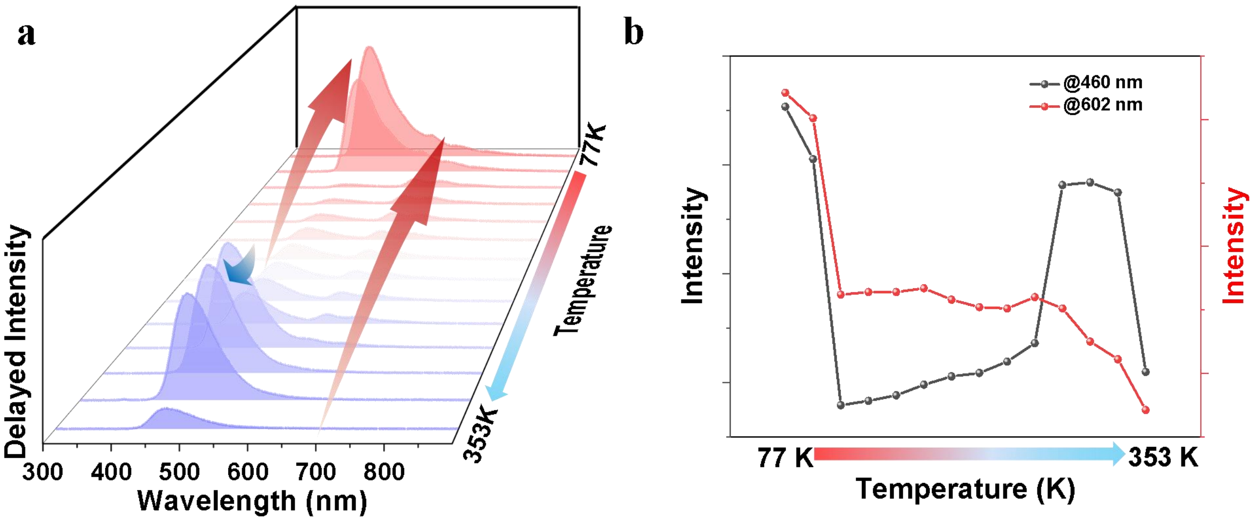

Fig. S32. a) Delayed spectra of **NDOH@PDOH** powder from 77 to 353 K. (λ_{ex}=365 nm, delayed time = 8 ms) b) The line chart displaying the afterglow intensities changes at 460 and 602 nm of **NDOH@PDOH** powder with increasing temperature.

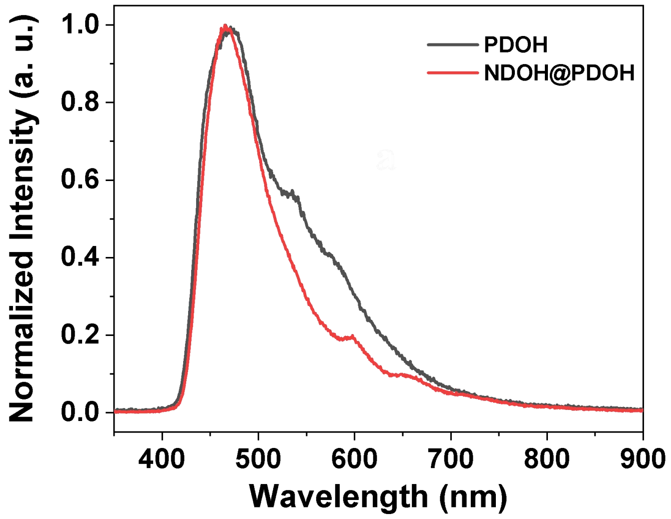

Fig. S33. The delayed emission spectra of **PDOH** and **NDOH@PDOH** powder at 77 K.

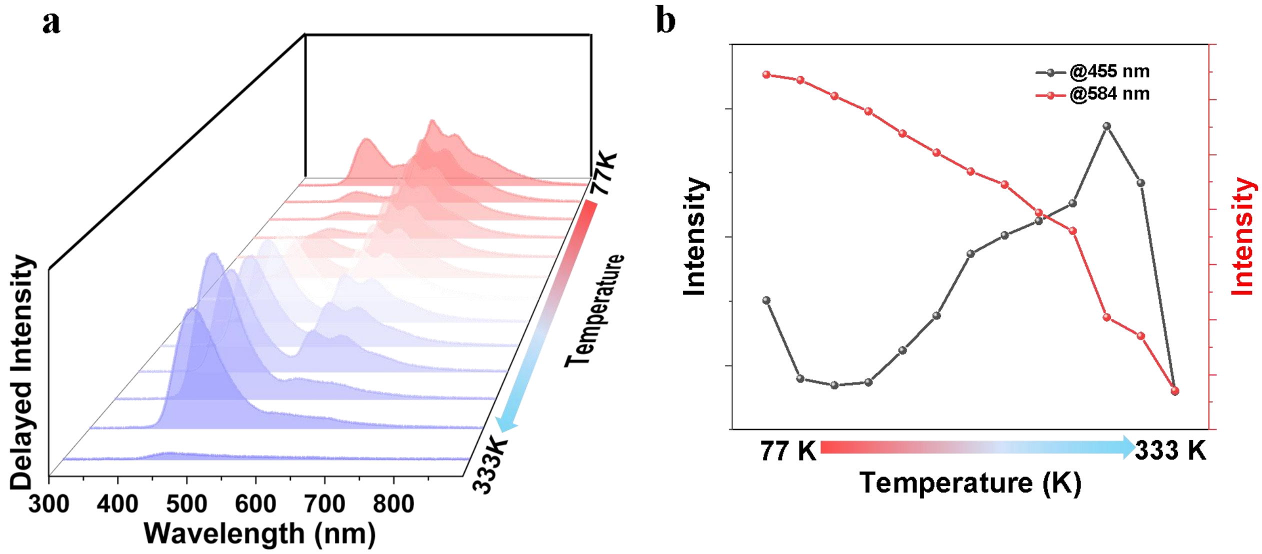

Fig. S34. a) Delayed spectra of **NDOH@PPOH** powder from 77 to 353 K. (λ_{ex}=365 nm, delayed time = 8 ms) b) The line chart displaying the afterglow intensities changes at 455 and 584 nm of **NDOH@PPOH** powder with increasing temperature.

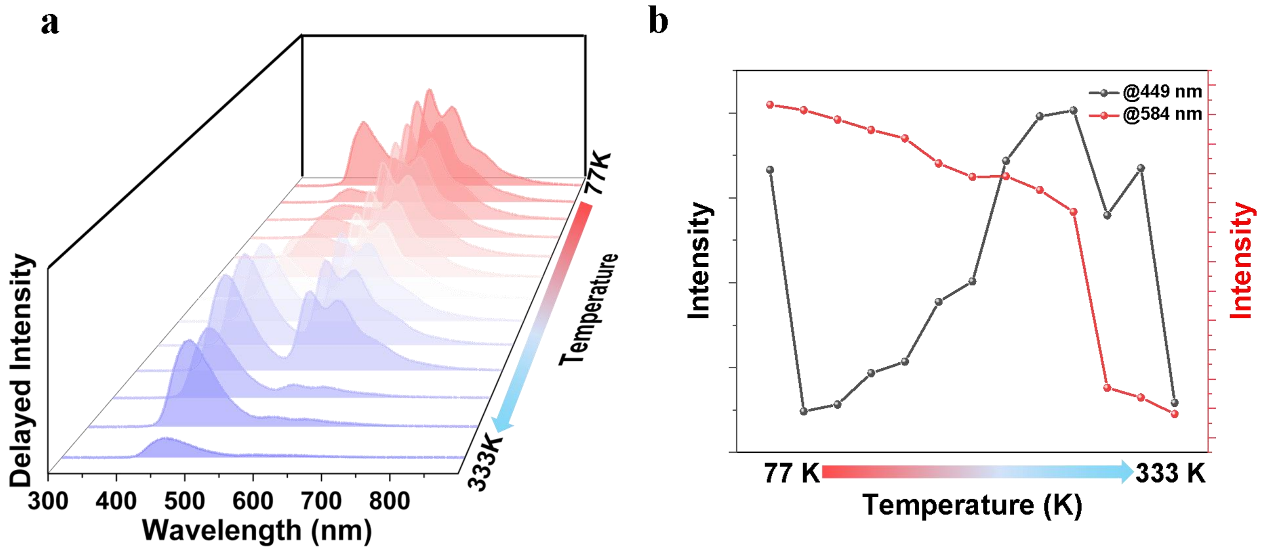

Fig. S35. a) Delayed spectra of **NDOH@PEOH** powder from 77 to 353 K. (λ_{ex}=365 nm, delayed time = 8 ms) b) The line chart displaying the afterglow intensities changes at 449 and 584 nm of **NDOH@PEOH** powder with increasing temperature.

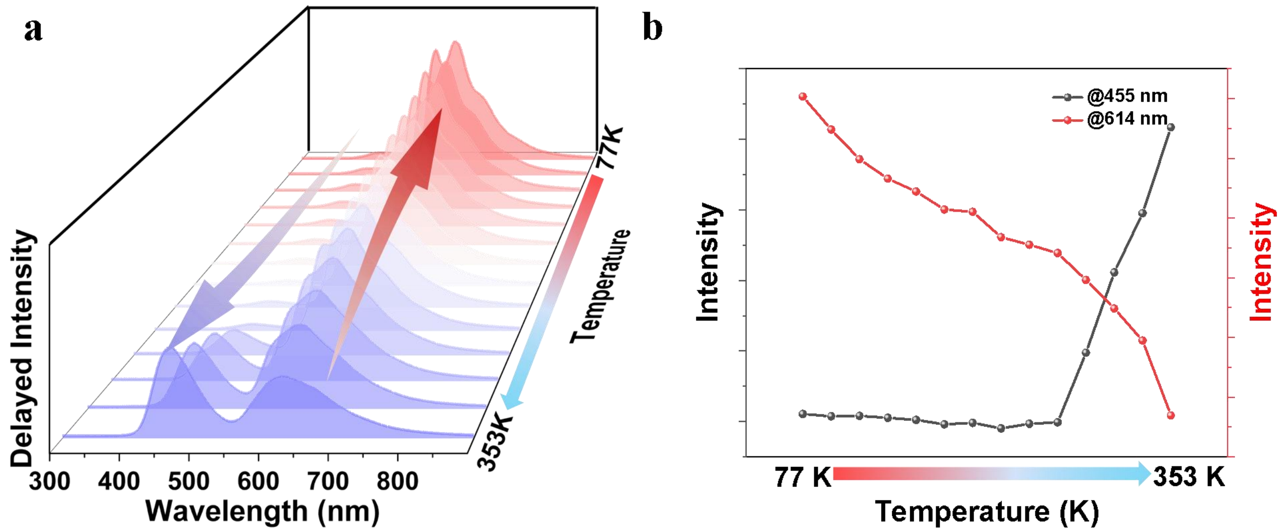

Fig. S36. a) Delayed spectra of **NDOH@PBOH** powder from 77 to 353 K. (λ_{ex}=365 nm, delayed time = 8 ms) b) The line chart displaying the afterglow intensities changes at 455 and 614 nm of **NDOH@PBOH** powder with increasing temperature.

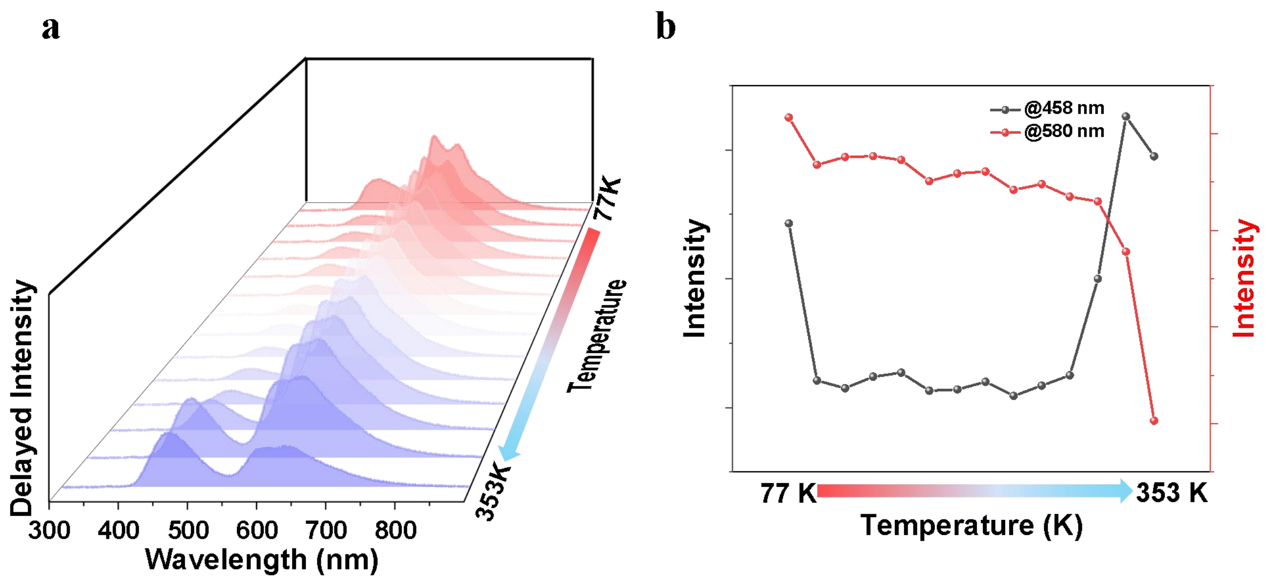

Fig. S37. a) Delayed spectra of **NDOH@PMOH** powder from 77 to 353 K. (λ_{ex}=365 nm, delayed time = 8 ms) b) The line chart displaying the afterglow intensities changes at 458 and 580 nm of **NDOH@PMOH** powder with increasing temperature.

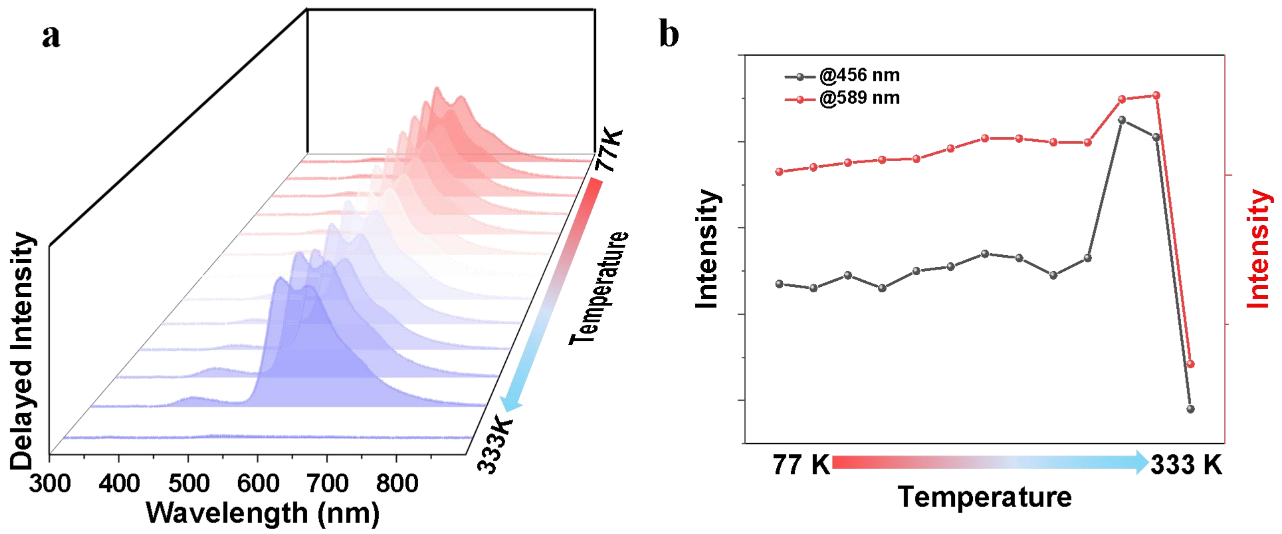

Fig. S38. a) Delayed spectra of **NDOH@PHOH** powder from 77 to 353 K. (λ_{ex}=365 nm, delayed time = 8 ms) b) The line chart displaying the afterglow intensities changes at 456 and 589 nm of **NDOH@PHOH** powder with increasing temperature.

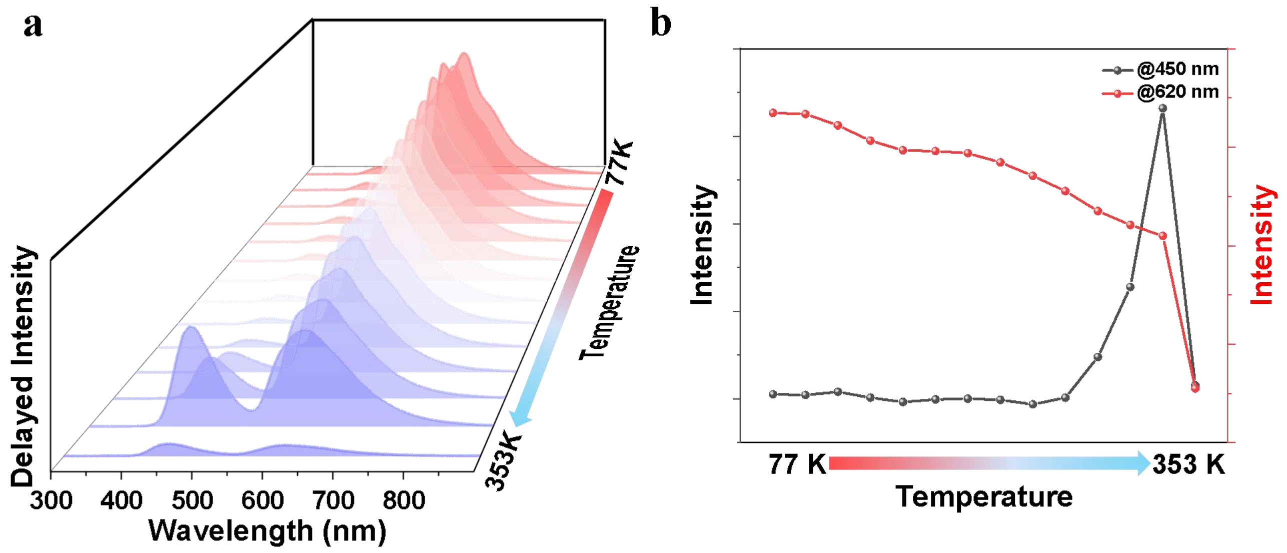

Fig. S39. A) Delayed spectra of **NDH@PBOH** powder from 77 to 353 K. (λ_{ex}=365 nm, delayed time = 8 ms) b) The line chart displaying the afterglow intensities changes at 450 and 620 nm of **NDH@PBOH** powder with increasing temperature.

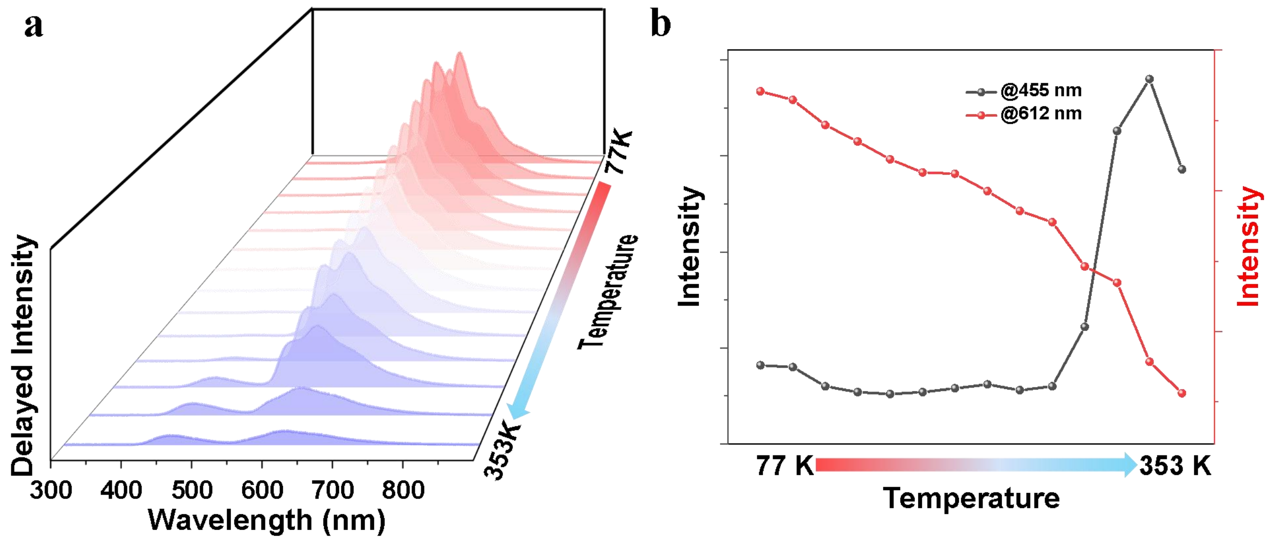

Fig. S40. A) Delayed spectra of **NDB@PBOH** powder from 77 to 353 K. (λ_{ex}=365 nm, delayed time = 8 ms) b) The line chart displaying the afterglow intensities changes at 455 and 612 nm of **NDB@PBOH** powder with increasing temperature.

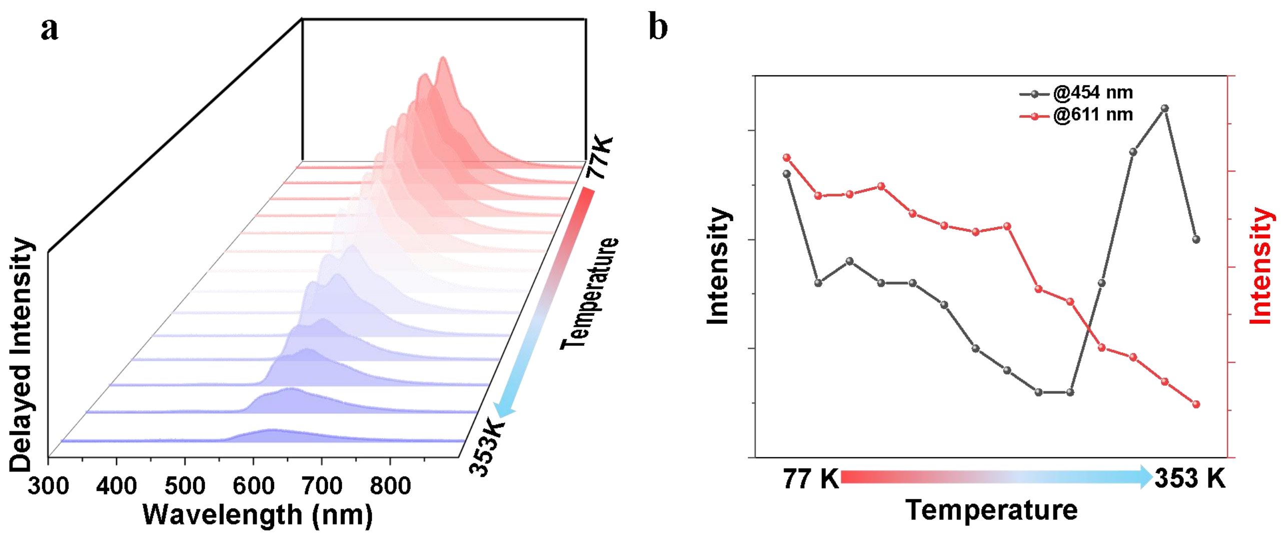

Fig. S41. a) Delayed spectra of **NDNC@PBOH** powder from 77 to 353 K. (λ_{ex}=365 nm, delayed time = 8 ms) b) The line chart displaying the afterglow intensities changes at 454 and 611 nm of **NDNC@PBOH** powder with increasing temperature.

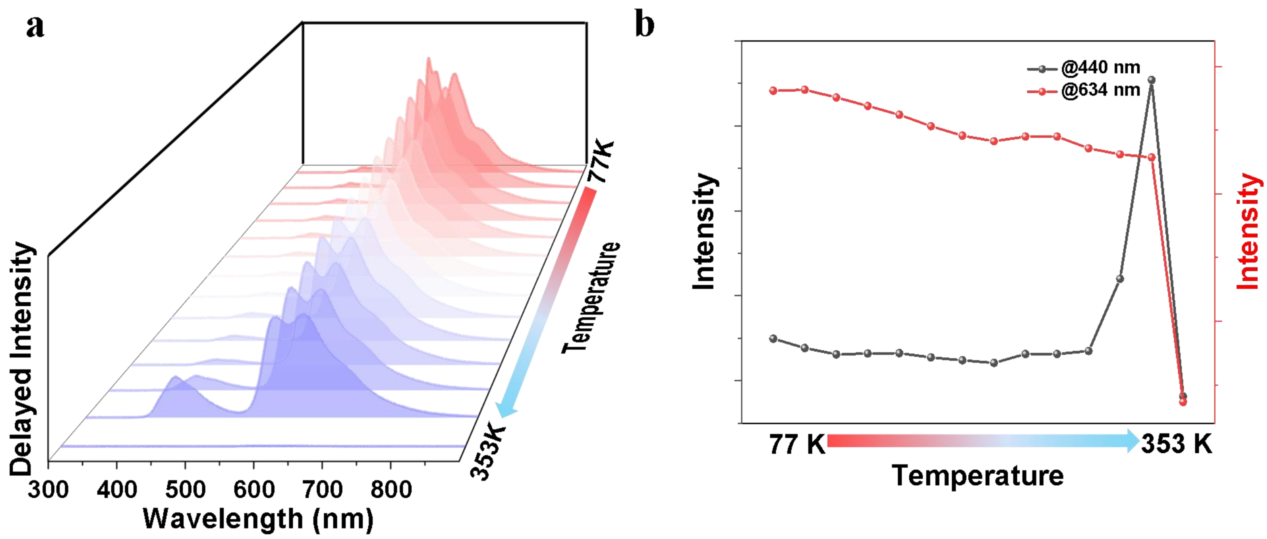

Fig. S42. a) Delayed spectra of **NDH@PBH** powder from 77 to 353 K. (λ_{ex}=365 nm, delayed time = 8 ms) b) The line chart displaying the afterglow intensities changes at 440 and 634 nm of **NDH@PBH** powder with increasing temperature.

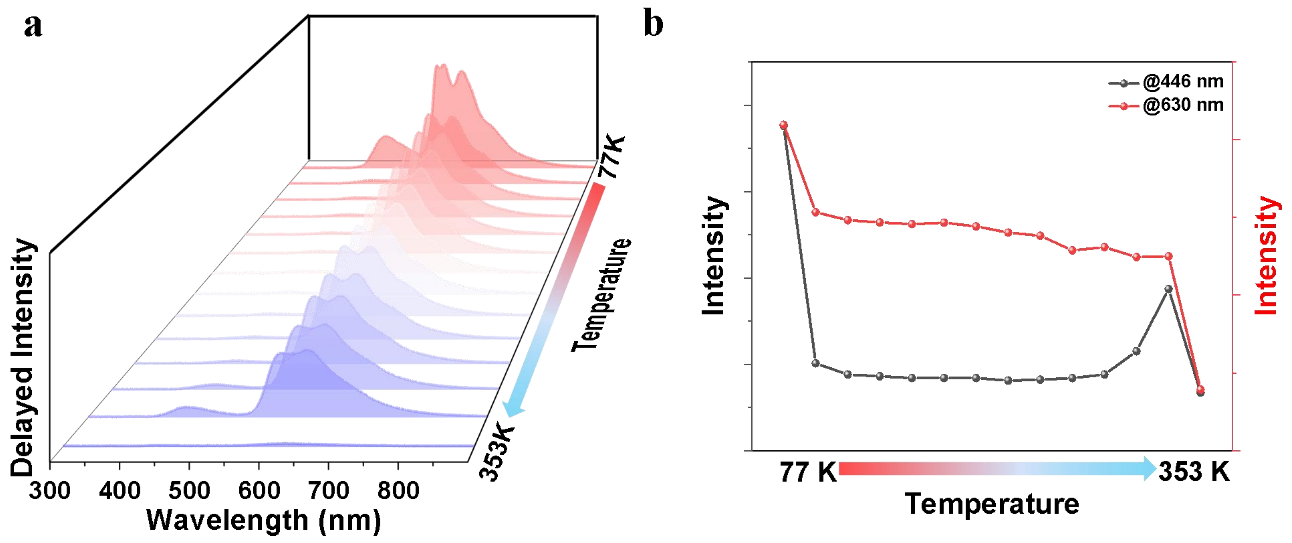

Fig. S43. A) Delayed spectra of **NDOH@PBH** powder from 77 to 353 K. (λ_{ex}=365 nm, delayed time = 8 ms) b) The line chart displaying the afterglow intensities changes at 446 and 630 nm of **NDOH@PBH** powder with increasing temperature.

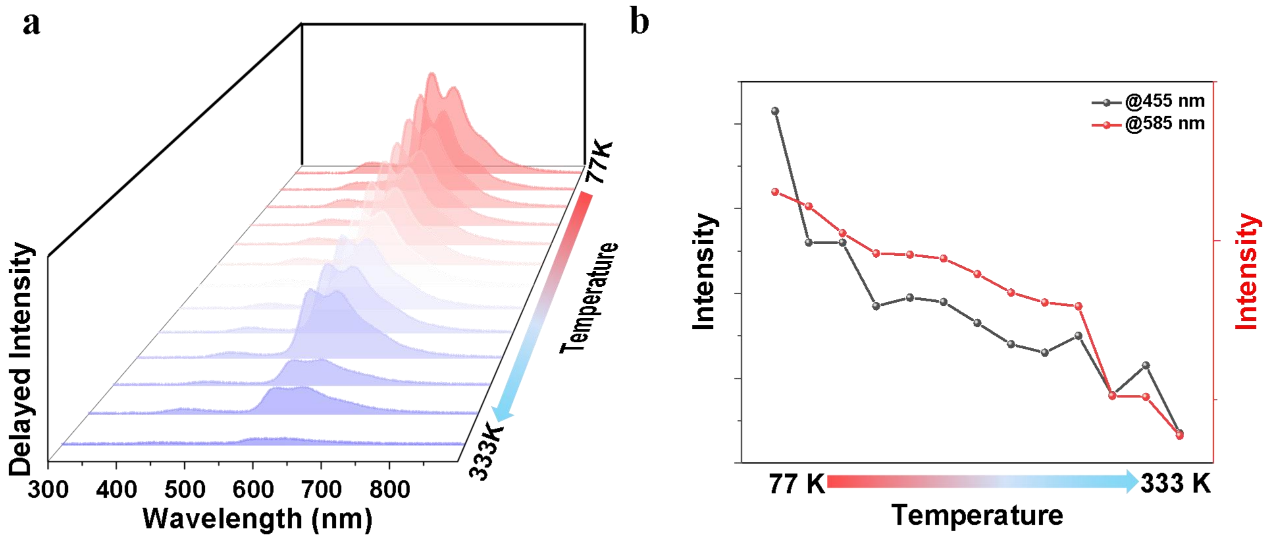

Fig. S44. A) Delayed spectra of **NDB@PBH** powder from 77 to 353 K. (λ_{ex}=365 nm, delayed time = 8 ms) b) The line chart displaying the afterglow intensities changes at 455 and 585 nm of **NDB@PBH** powder with increasing temperature.

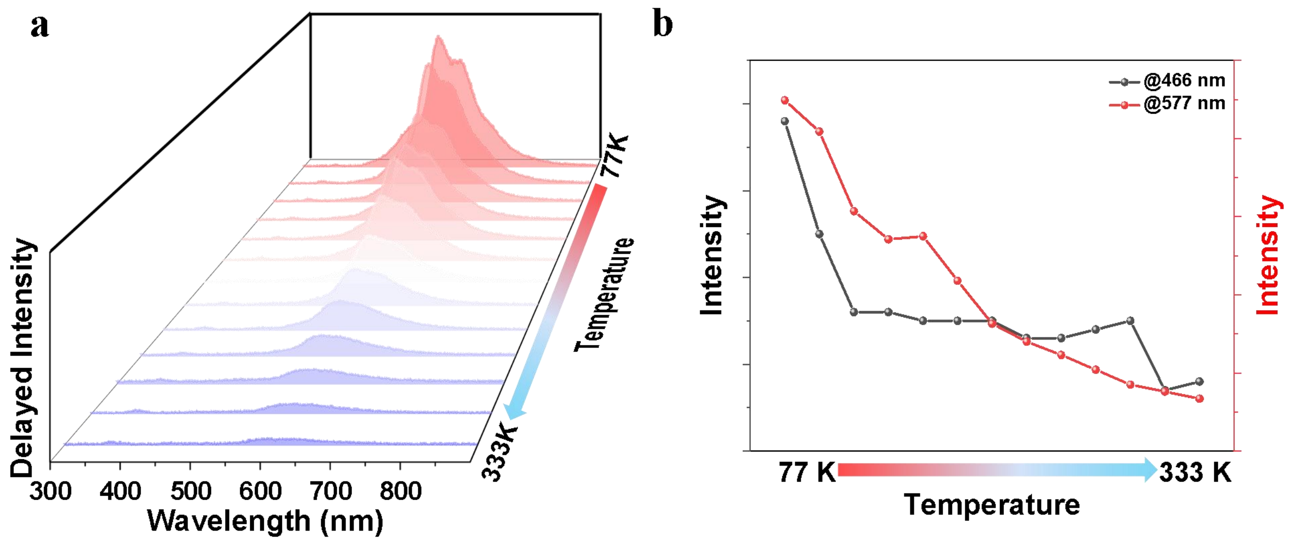

Fig. S45. A) Delayed spectra of **NDNC@PBH** powder from 77 to 353 K. (λ_{ex}=365 nm, delayed time = 8 ms) b) The line chart displaying the afterglow intensities changes at 466 and 577 nm of **NDNC@PBH** powder with increasing temperature.

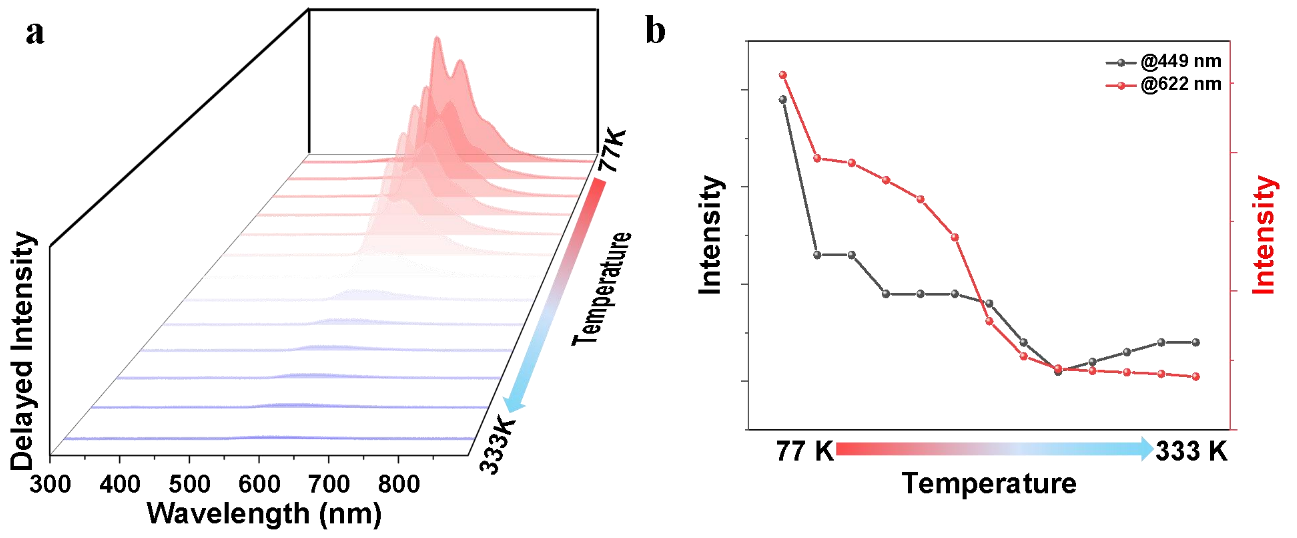

Fig. S46. A) Delayed spectra of **NDH@PBB** powder from 77 to 353 K. (λ_{ex}=365 nm, delayed time = 8 ms) b) The line chart displaying the afterglow intensities changes at 449 and 622 nm of **NDH@PBB** powder with increasing temperature.

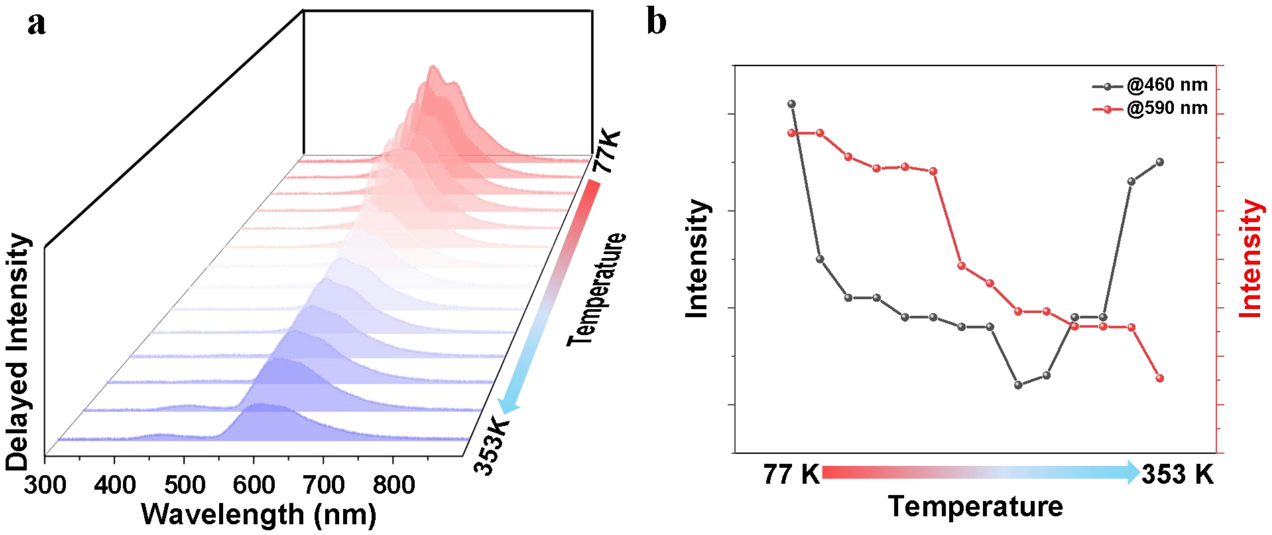

Fig. S47. A) Delayed spectra of **NDOH@PBB** powder from 77 to 353 K. (λ_{ex}=365 nm, delayed time = 8 ms) b) The line chart displaying the afterglow intensities changes at 460 and 590 nm of **NDOH@PBB** powder with increasing temperature.

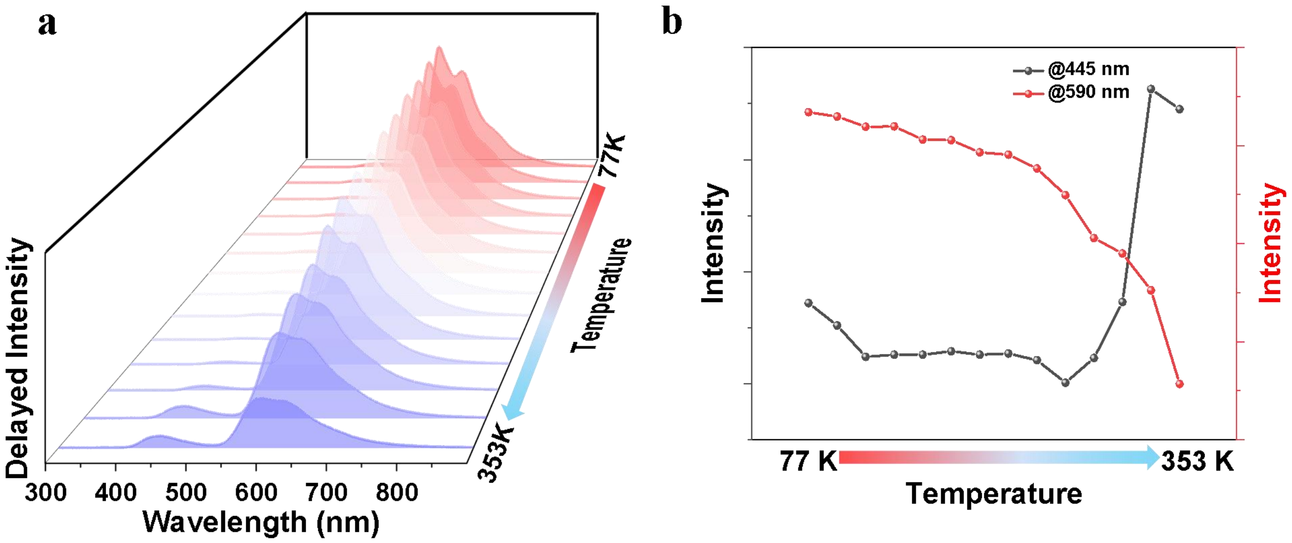

Fig. S48. A) Delayed spectra of **NDB@PBB** powder from 77 to 353 K. (λ_{ex}=365 nm, delayed time = 8 ms) b) The line chart displaying the afterglow intensities changes at 445 and 590 nm of **NDB@PBB** powder with increasing temperature.

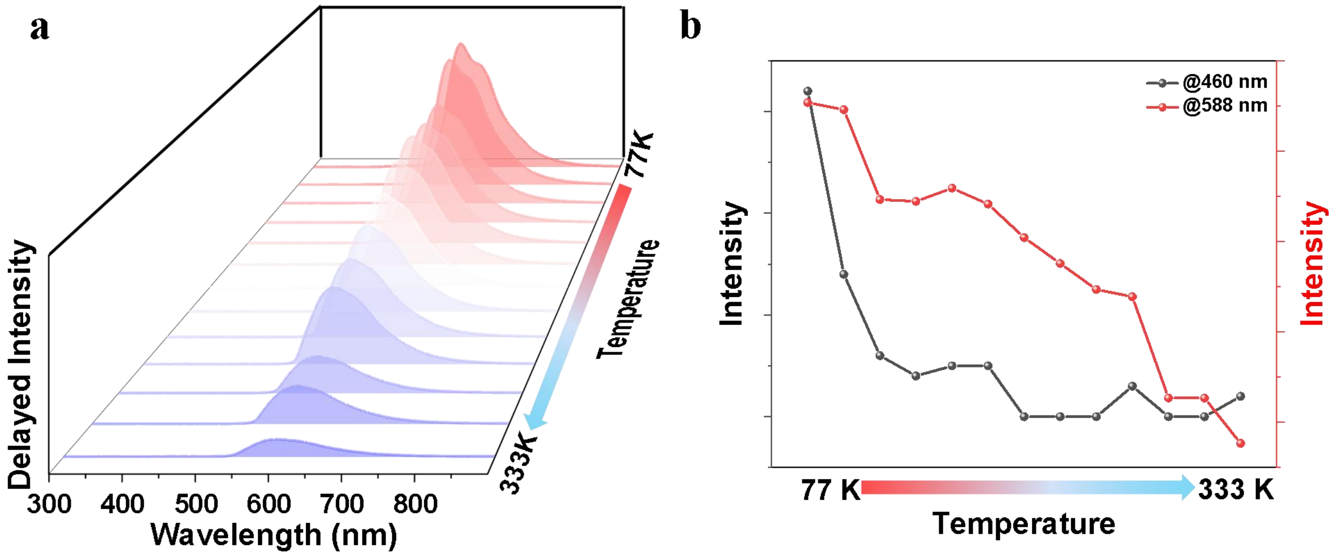

Fig. S49. A) Delayed spectra of **NDNC@PBB** powder from 77 to 353 K. (λ_{ex}=365 nm, delayed time = 8 ms) b) The line chart displaying the afterglow intensities changes at 460 and 588 nm of **NDNC@PBB** powder with increasing temperature.

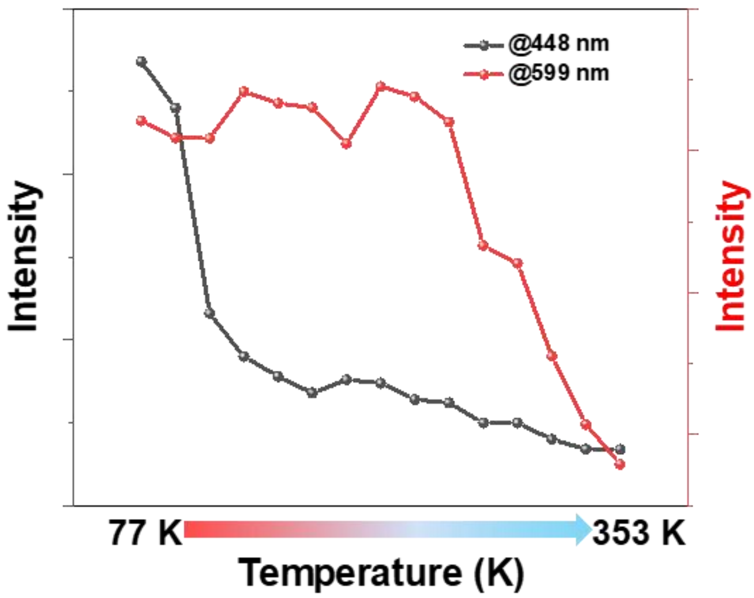

Fig. S50. The line chart displaying the afterglow intensities changes at 448 and 599 nm of **NDNC@PBNC** powder with increasing temperature.

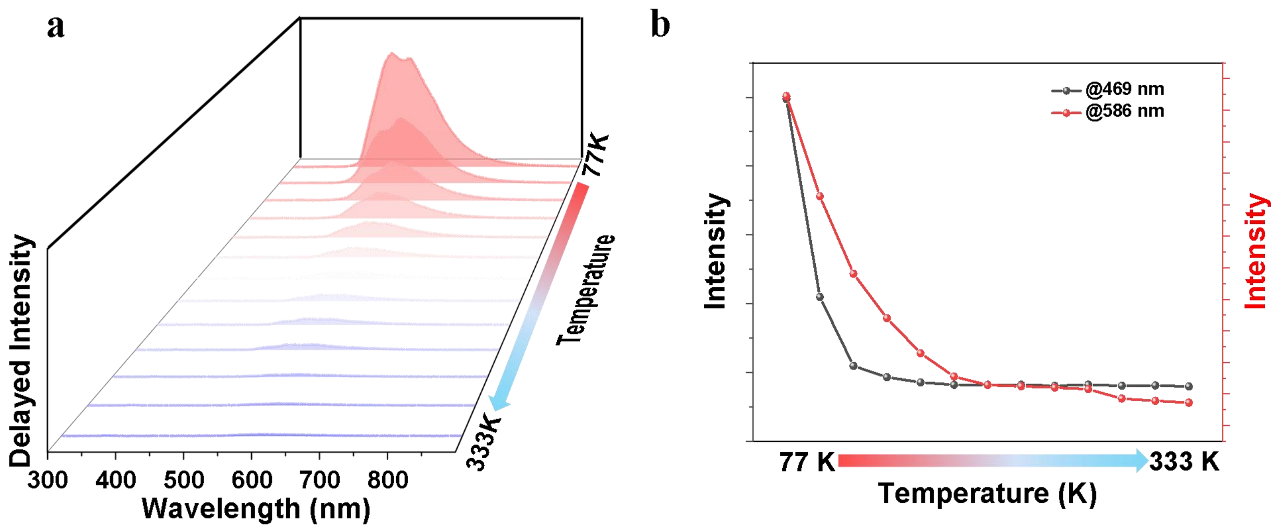

Fig. S51. a) Delayed spectra of **NDH@PBNC** powder from 77 to 353 K. (λ_{ex}=365 nm, delayed time = 8 ms) b) The line chart displaying the afterglow intensities changes at 469 and 586 nm of **NDH@PBNC** powder with increasing temperature.

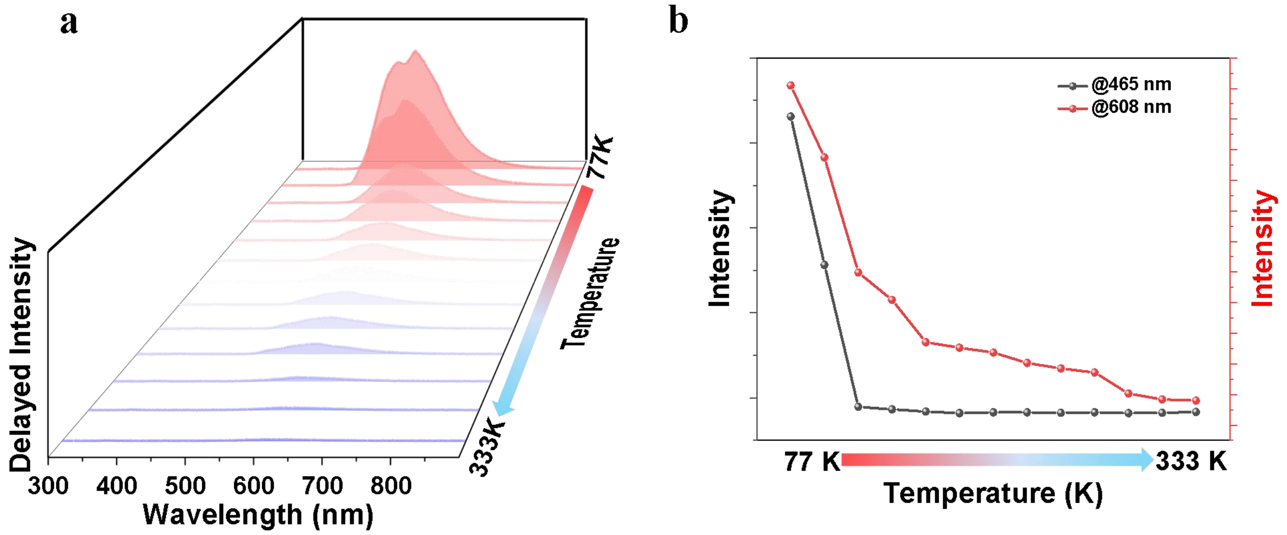

Fig. S52. a) Delayed spectra of **NDOH@PBNC** powder from 77 to 353 K. (λ_{ex}=365 nm, delayed time = 8 ms) b) The line chart displaying the afterglow intensities changes at 465 and 608 nm of **NDOH@PBNC** powder with increasing temperature.

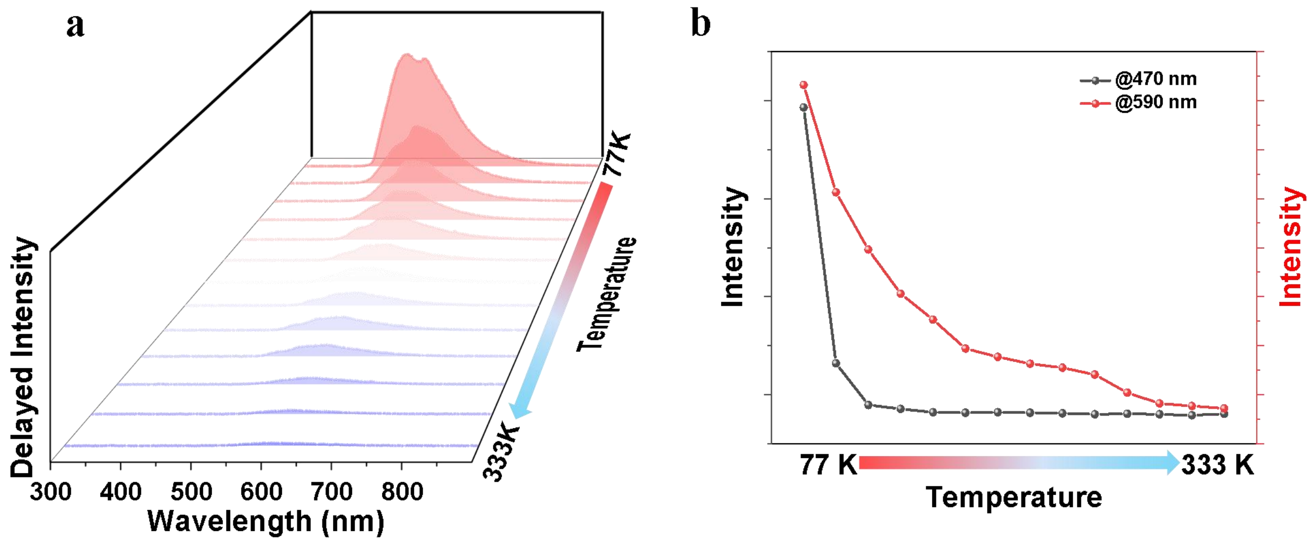

Fig. S53. a) Delayed spectra of **NDB@PBNC** powder from 77 to 353 K. (λ_{ex}=365 nm, delayed time = 8 ms) b) The line chart displaying the afterglow intensities changes at 470 and 590 nm of **NDB@PBNC** powder with increasing temperature.

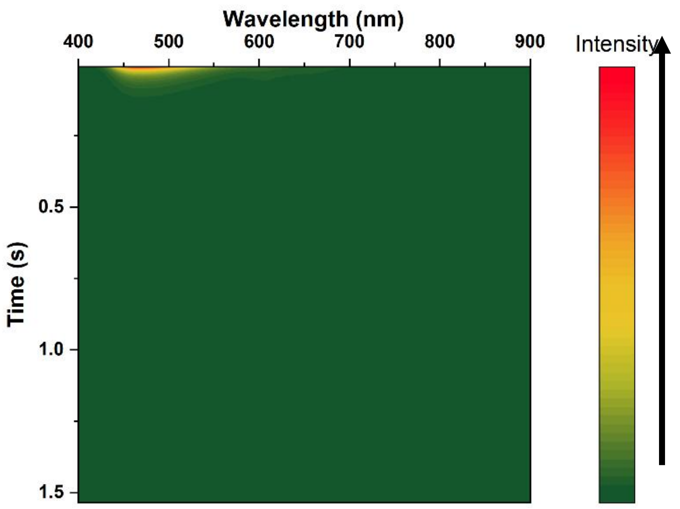

Fig. S54. The time-resolved delayed spectra of **NDOH@PDOH** powder (λ_{ex}=365 nm, at 298 K).

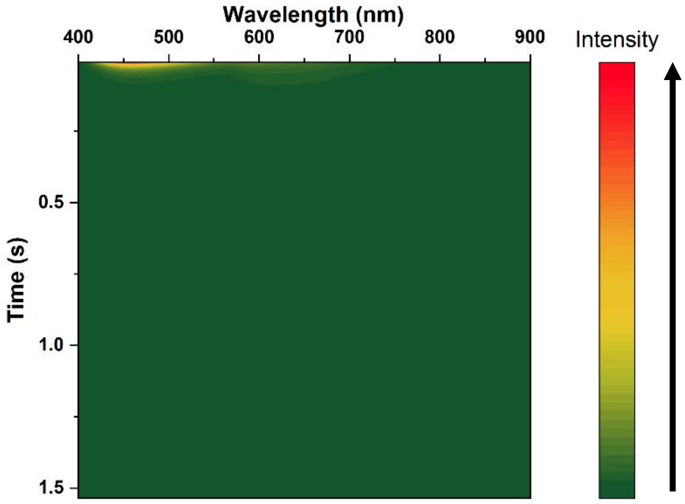

Fig. S55. The time-resolved delayed spectra of **NDOH@PPOH** powder (λ_{ex}=365 nm, at 298 K).

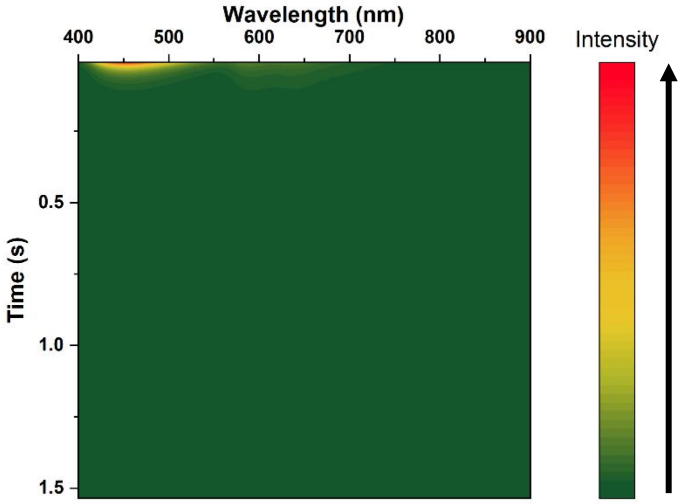

Fig. S56. The time-resolved delayed spectra of **NDOH@PEOH** powder (λ_{ex}=365 nm, at 298 K).

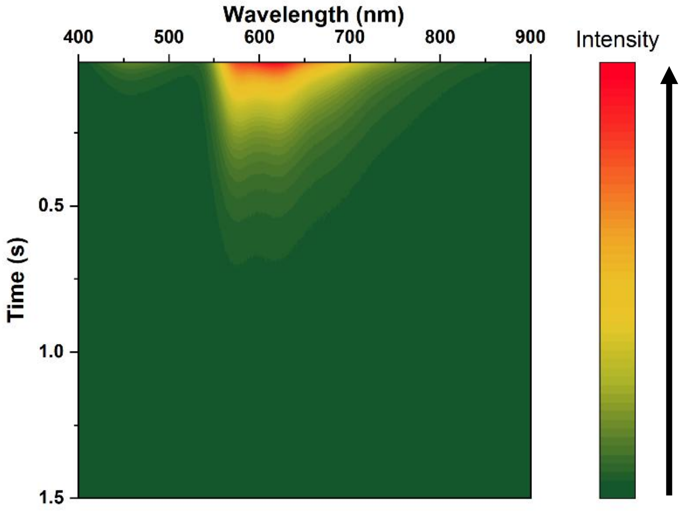

Fig. S57. The time-resolved delayed spectra of **NDOH@PBOH** powder (λ_{ex}=365 nm, at 298 K).

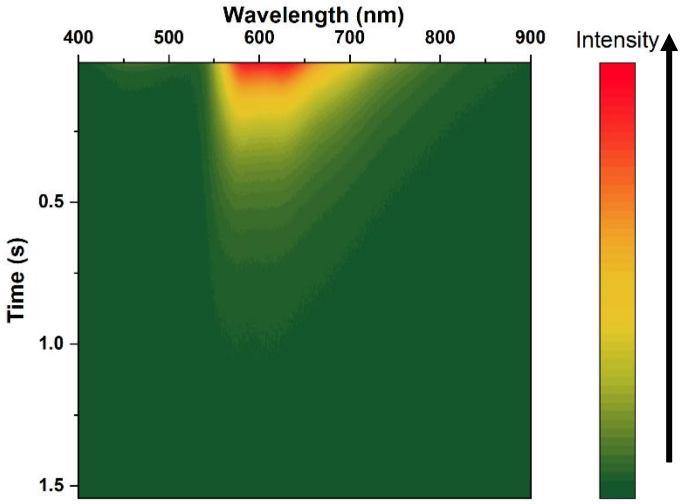

Fig. S58. The time-resolved delayed spectra of **NDOH@PMOH** powder (λ_{ex}=365 nm, at 298 K).

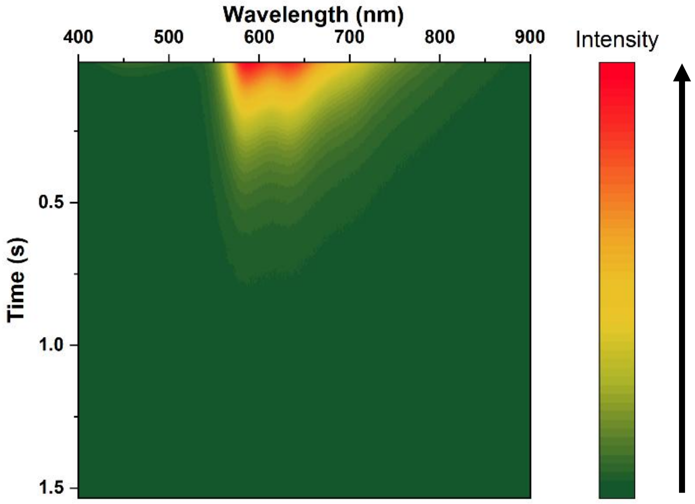

Fig. S59. The time-resolved delayed spectra of **NDOH@PHOH** powder (λ_{ex}=365 nm, at 298 K).

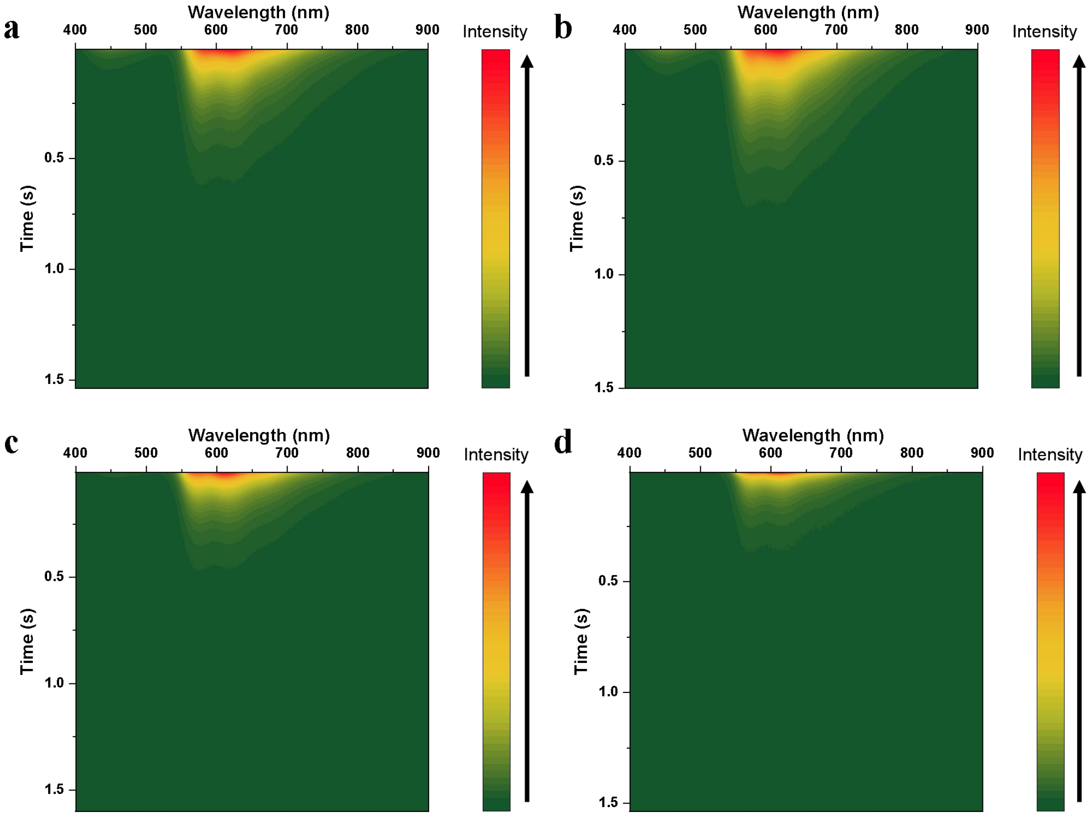

Fig. S60. The time-resolved delayed spectra of **NDH@PBOH**, **NDOH@PBOH**, **NDB@PBOH** and **NDNC@PBOH** powder (λ_{ex}=365 nm, at 298 K).

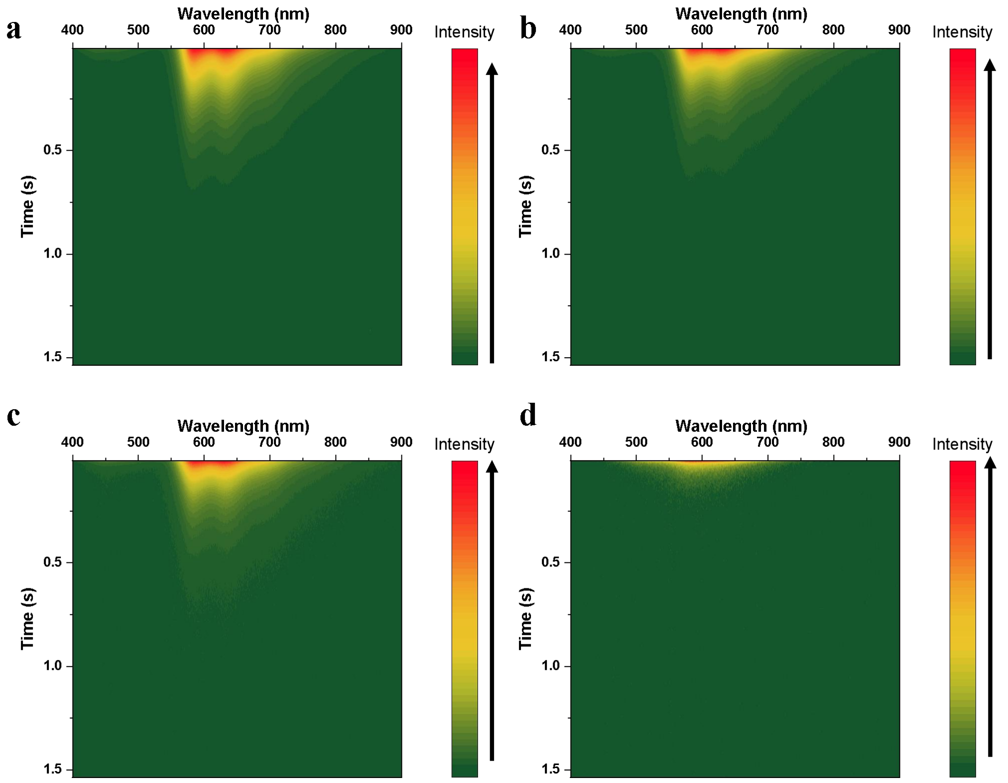

Fig. S61. The time-resolved delayed spectra of **NDH@PBH**, **NDOH@PBH**, **NDB@PBH** and **NDNC@PBH** powder (λ_{ex}=365 nm, at 298 K).

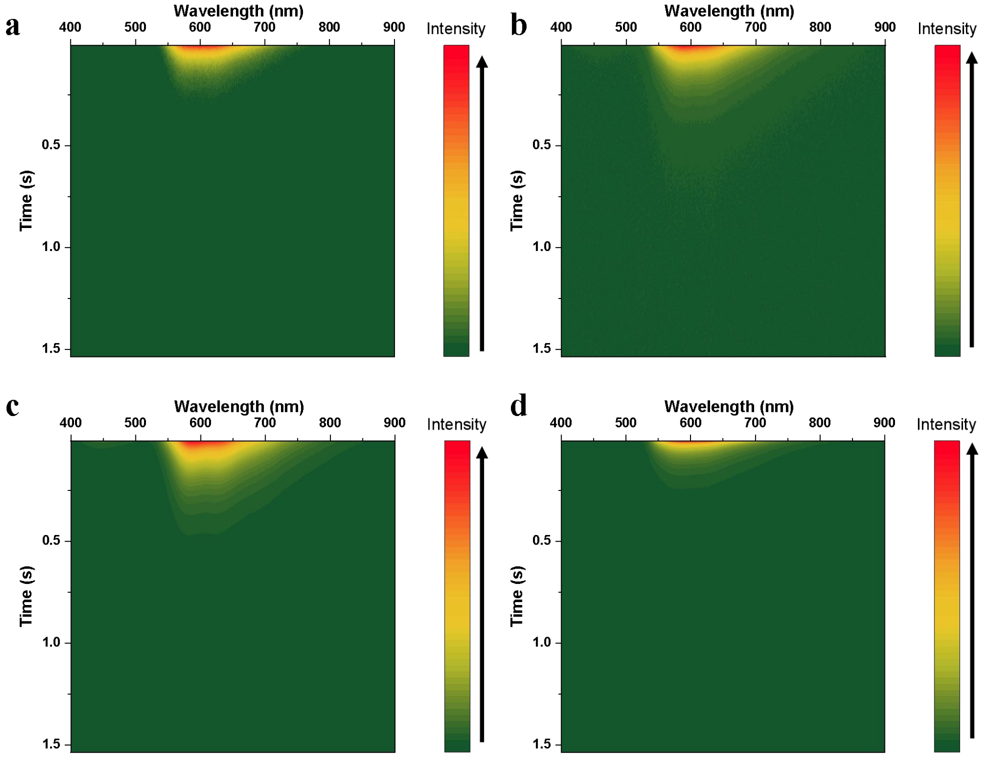

Fig. S62. The time-resolved delayed spectra of **NDH@PBB**, **NDOH@PBB**, **NDB@PBB** and **NDNC@PBB** powder (λ_{ex}=365 nm, at 298 K).

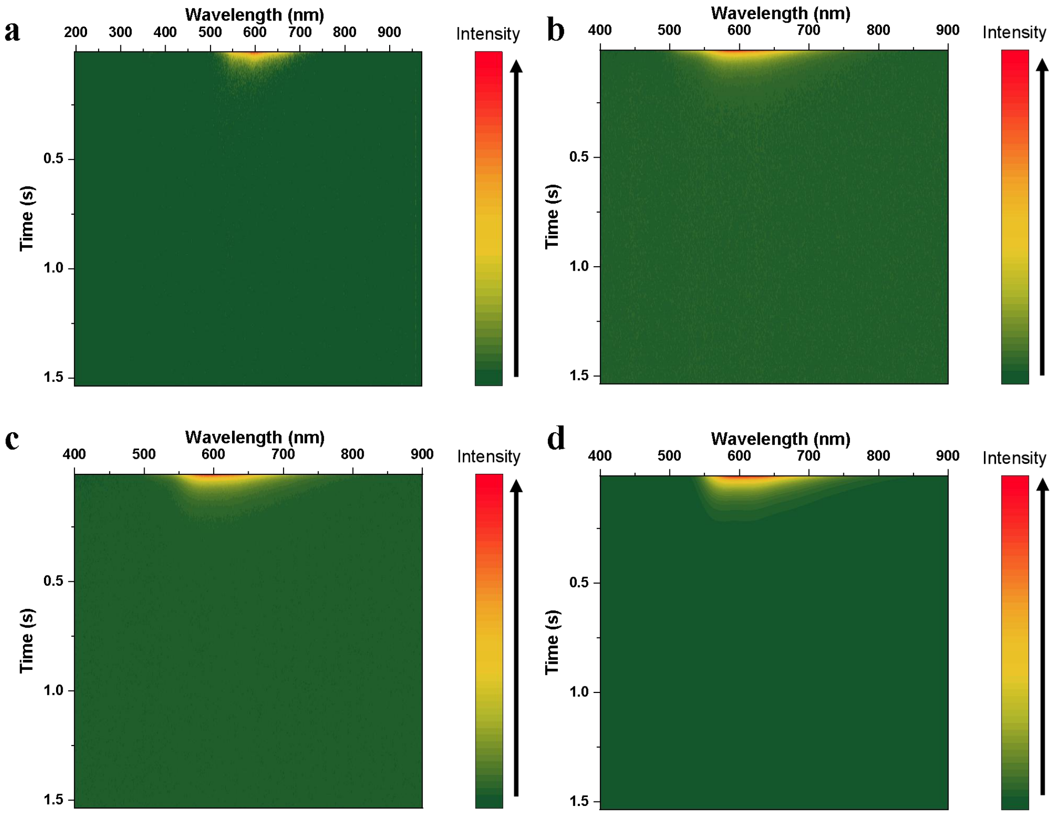

Fig. S63. The time-resolved delayed spectra of **NDH@PBNC**, **NDOH@PBNC**, **NDB@PBNC** and **NDNC@PBNC** powder (λ_{ex}=365 nm, at 298 K).

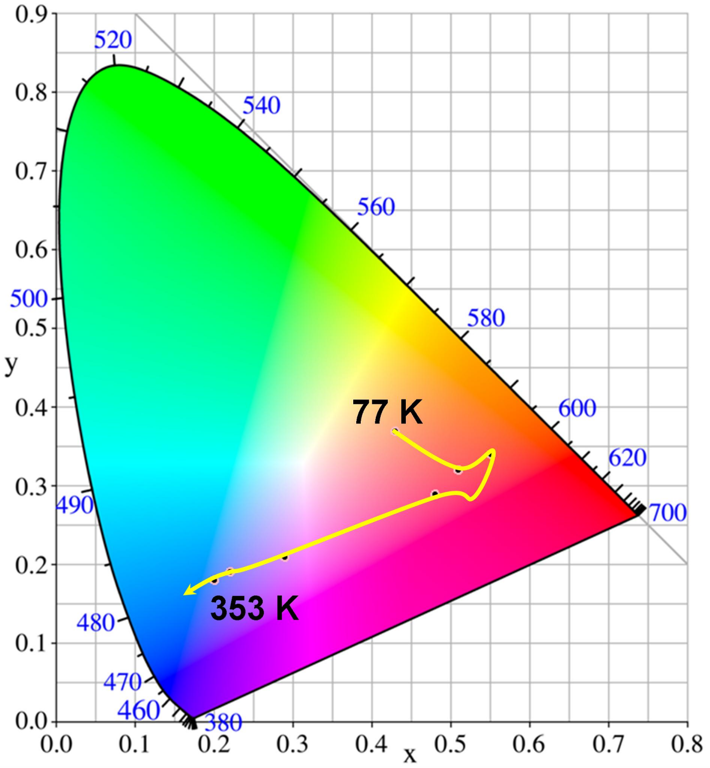

Fig. S64. Chromatic CIE chromaticity coordinates based on the delayed spectra of **NDOH@POOH** powder at different temperatures.

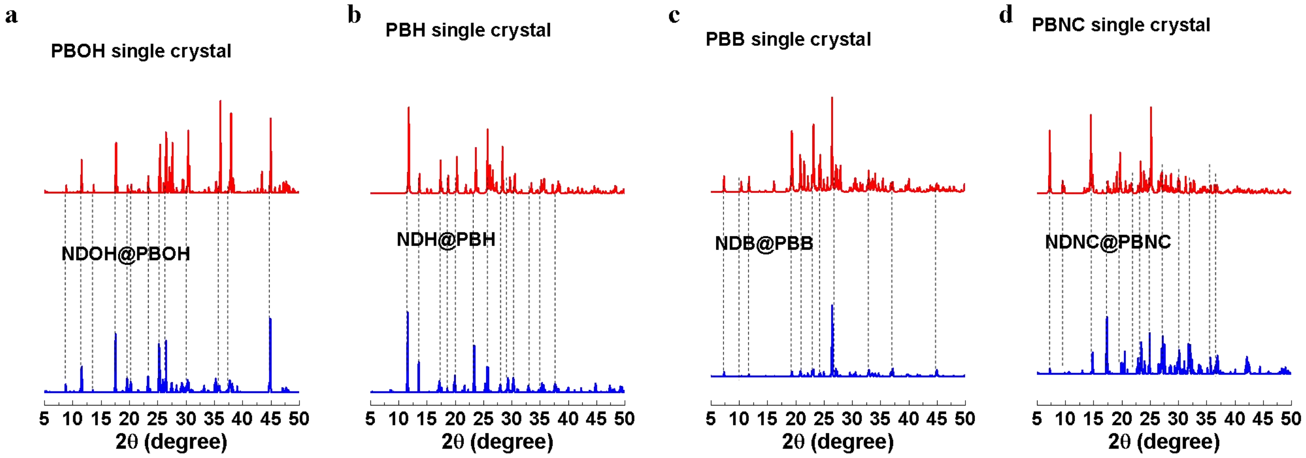

Fig. S65. The powder X-ray diffraction (PXRD) patterns of **PBOH** single crystal and **NDOH@PBOH** powder (a), **PBH** single crystal and **NDH@PBH** powder (b), **PBB** single crystal and **NDB@PBB** powder (c) and **PBNC** single crystal and **NDNC@PBNC** powder.

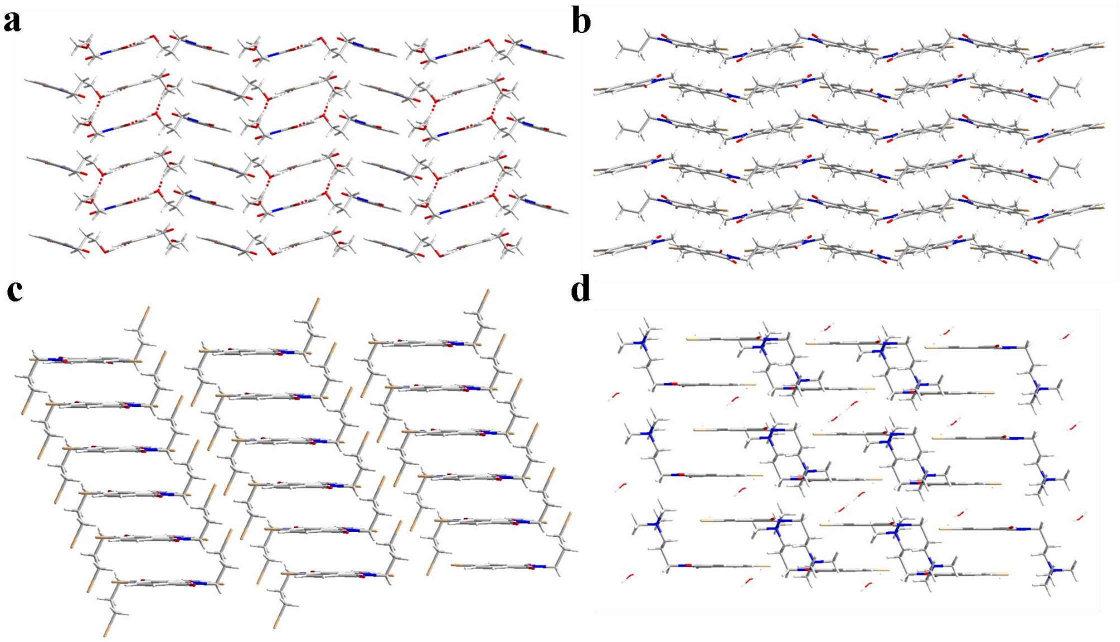

Fig. S66. The crystal stacking modes of **PBOH**, **PBH**, **PBB** and **PBNC** single crystals.

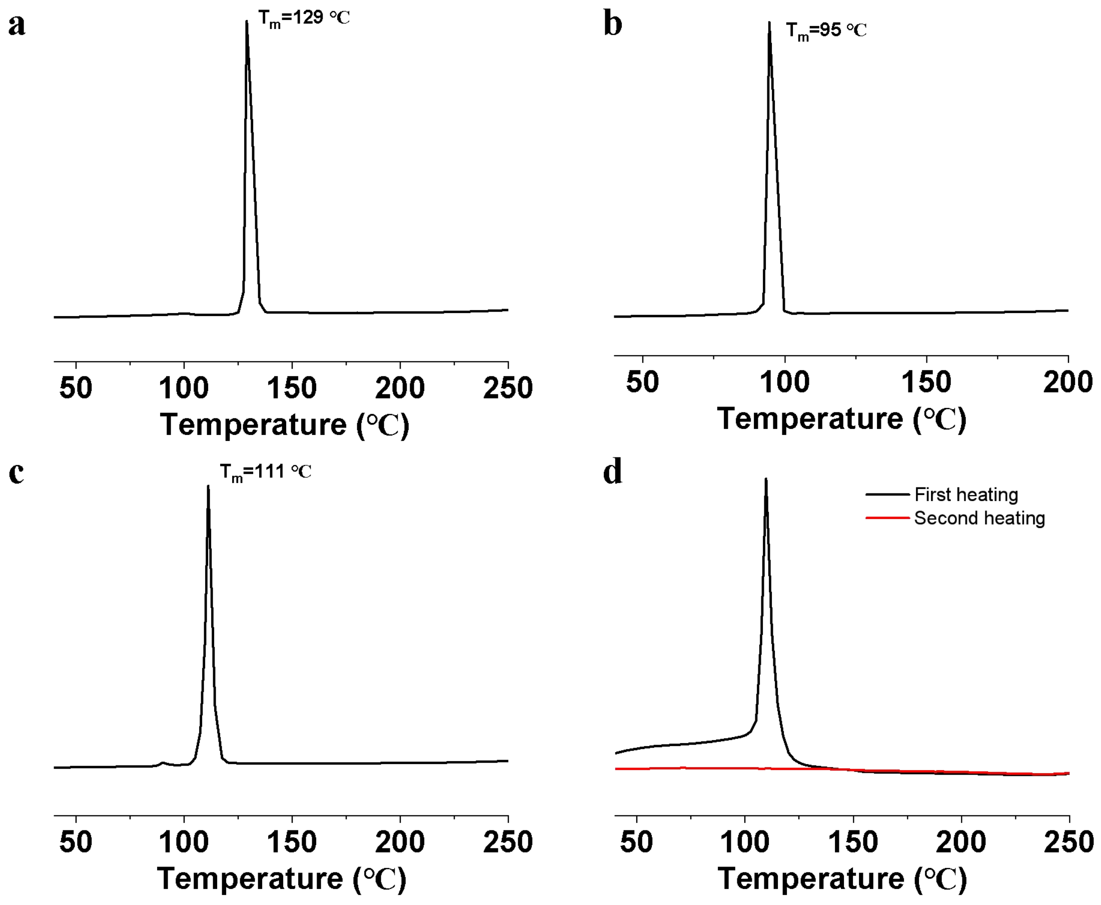

Fig. S67. The DSC curve and melting point of **PBOH**, **PBH**, **PBB** and **PBNC** crystals.

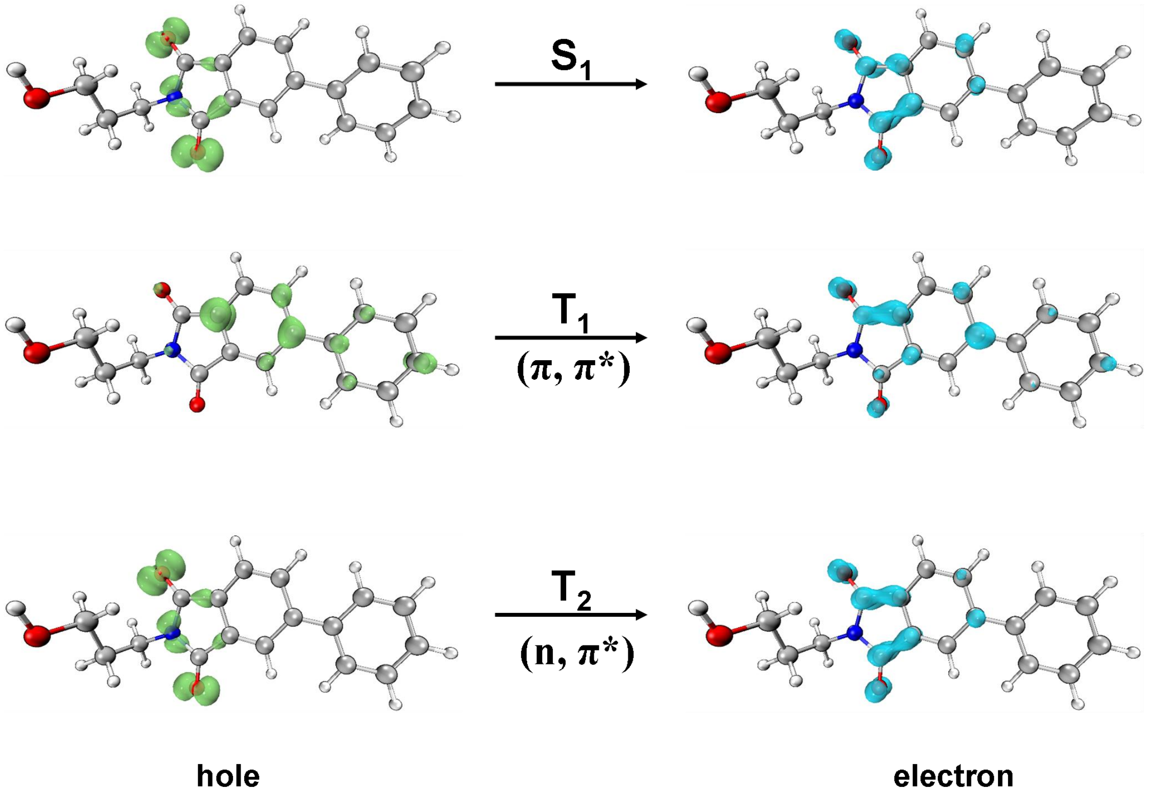

Fig. S68. Hole and electron analyses of $S_0 \rightarrow S_1$, $T_1 \rightarrow S_0$ and $T_2 \rightarrow S_0$ transitions of **PPOH**.

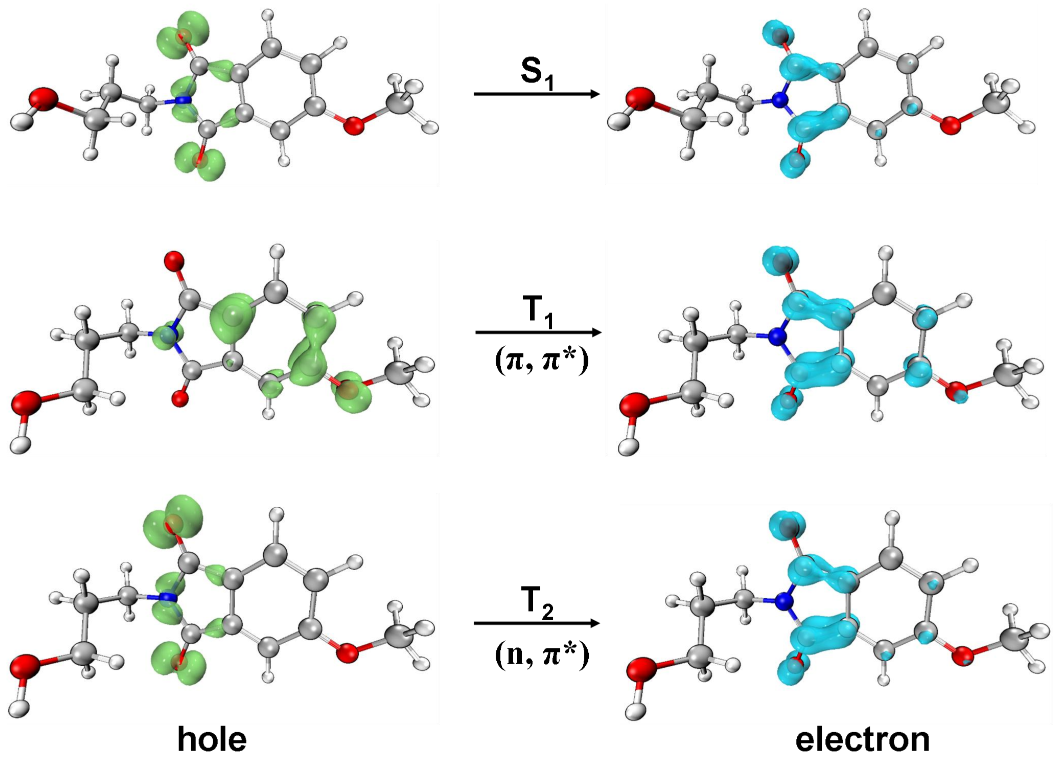

Fig. S69. Hole and electron analyses of $S_0 \rightarrow S_1$, $T_1 \rightarrow S_0$ and $T_2 \rightarrow S_0$ transitions of **POOH**.

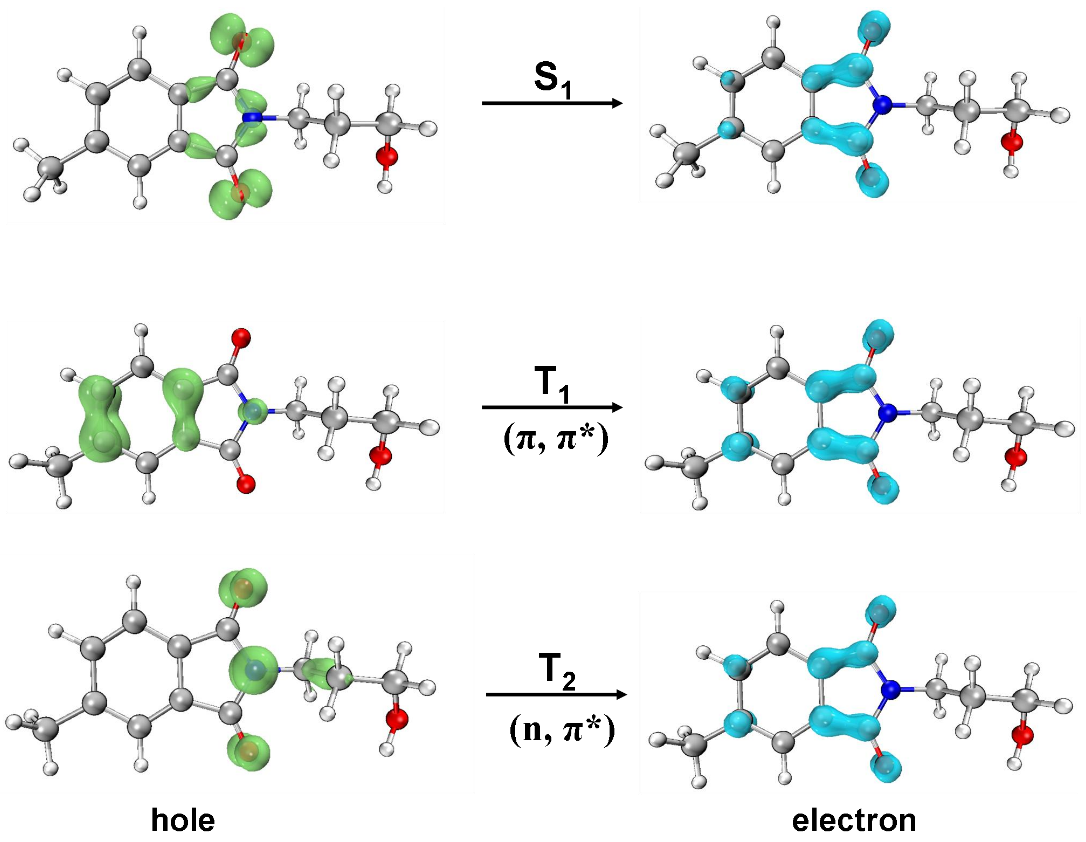

Fig. S70. Hole and electron analyses of $S_0 \rightarrow S_1$, $T_1 \rightarrow S_0$ and $T_2 \rightarrow S_0$ transitions of **PMOH**.

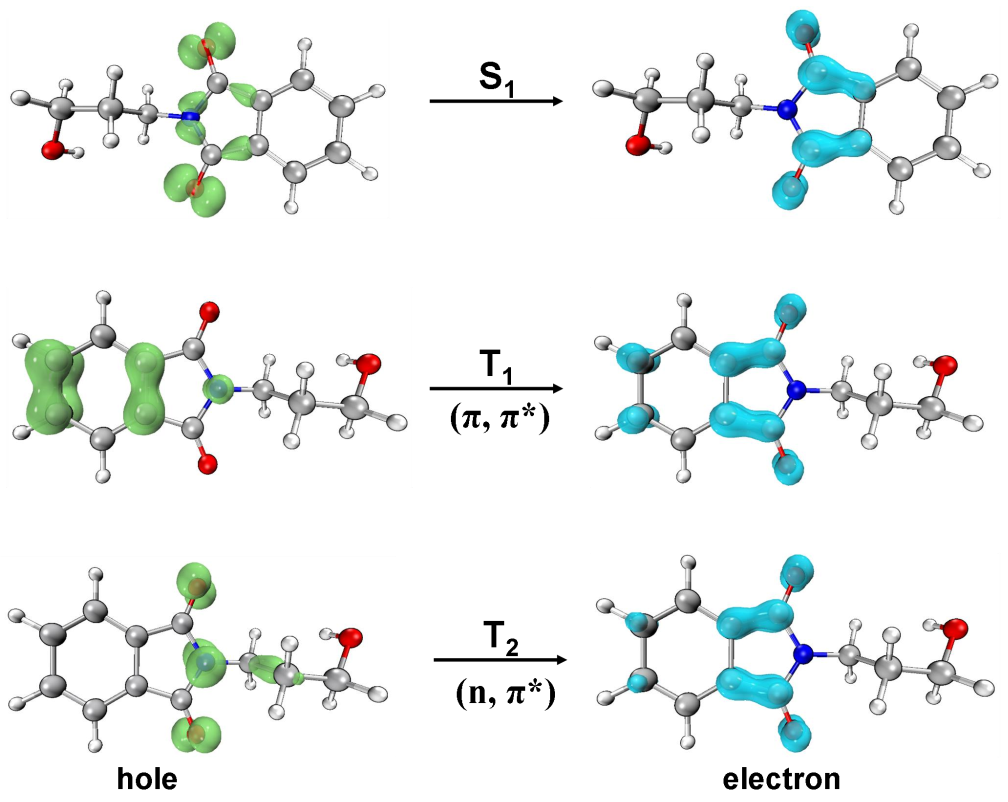

Fig. S71. Hole and electron analyses of $S_0 \rightarrow S_1$, $T_1 \rightarrow S_0$ and $T_2 \rightarrow S_0$ transitions of **PHOH**.

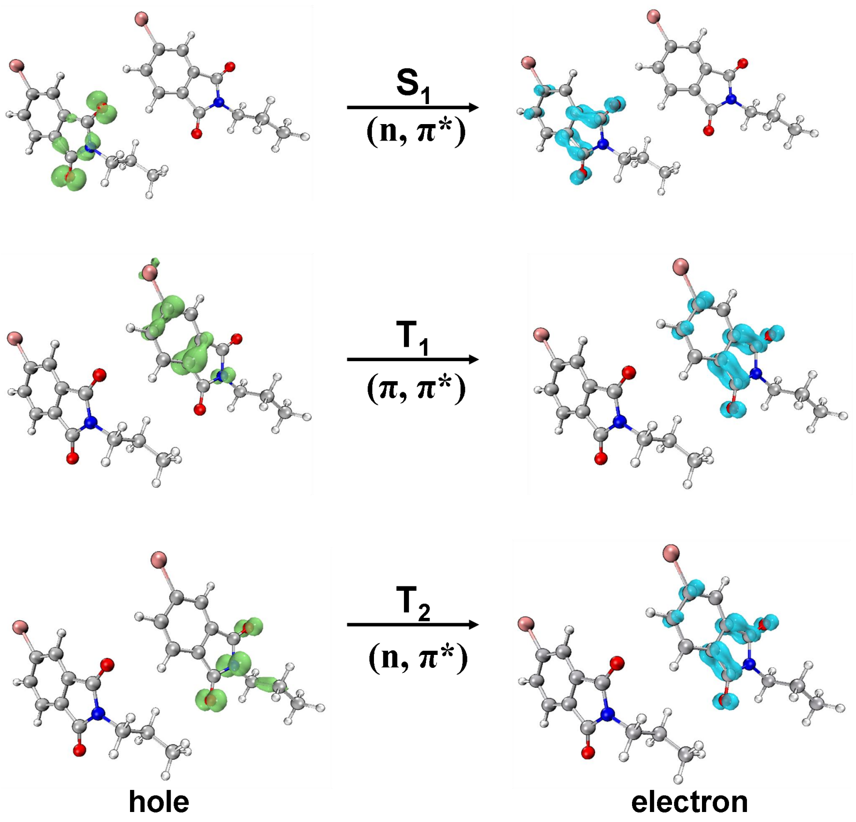

Fig. S72. Hole and electron analyses of $S_0 \rightarrow S_1$, $T_1 \rightarrow S_0$ and $T_2 \rightarrow S_0$ transitions of **PBH** dimer.

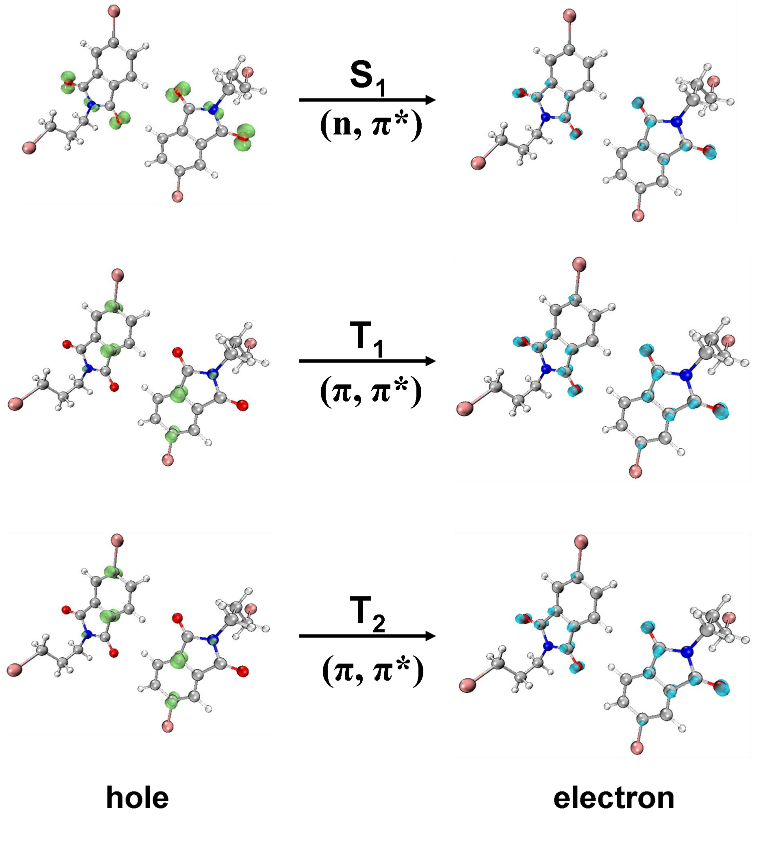

Fig. S73. Hole and electron analyses of $S_0 \rightarrow S_1$, $T_1 \rightarrow S_0$ and $T_2 \rightarrow S_0$ transitions of **PBB** dimer.

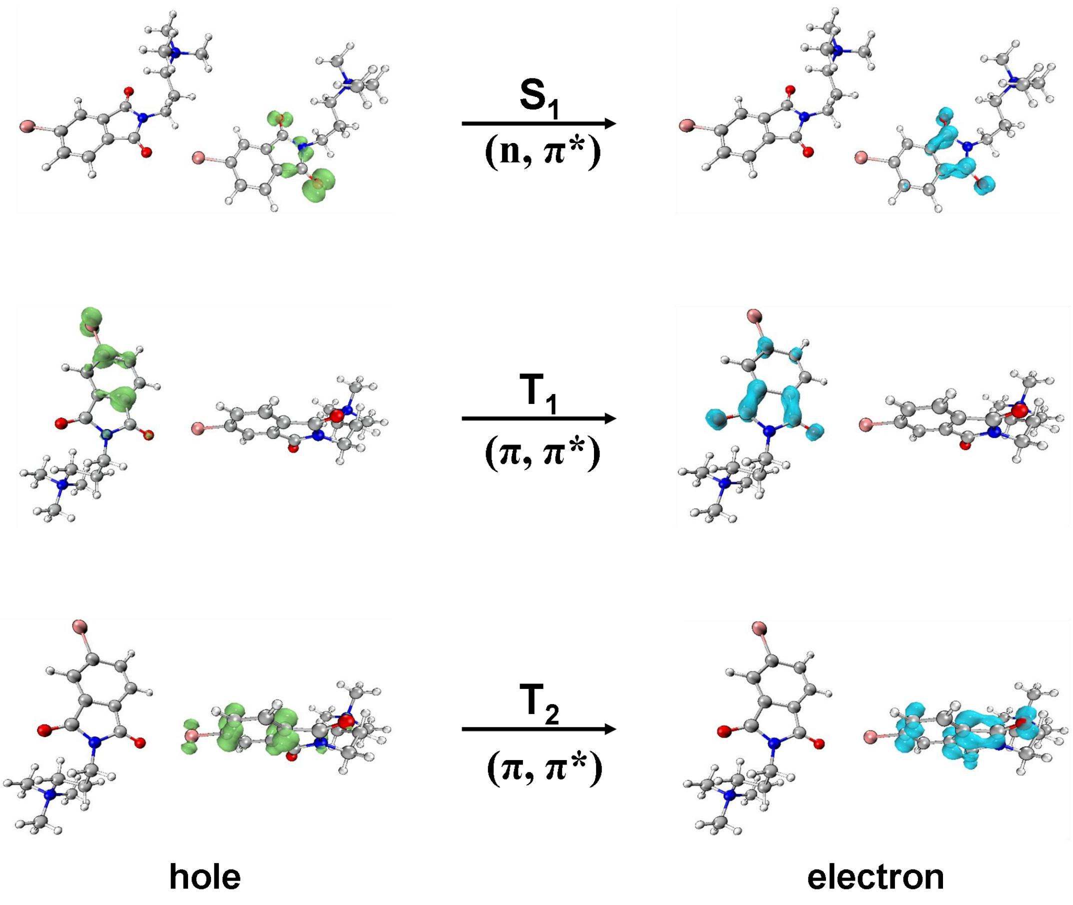

Fig. S74. Hole and electron analyses of $S_0 \rightarrow S_1$, $T_1 \rightarrow S_0$ and $T_2 \rightarrow S_0$ transitions of **PBNC** dimer.

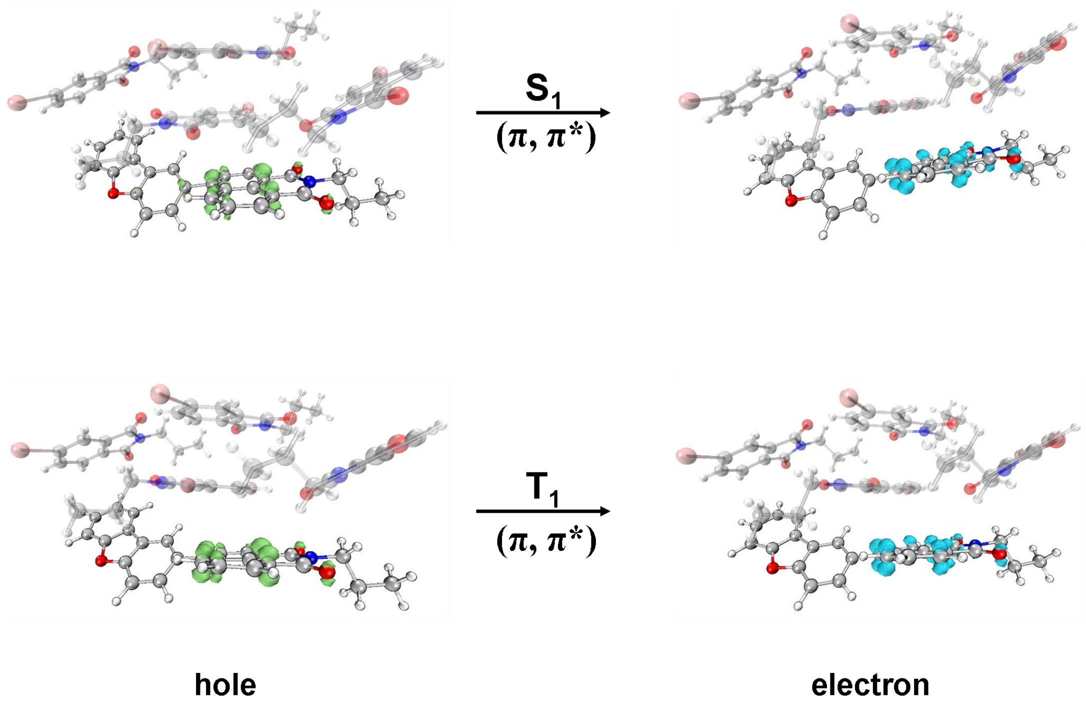

Fig. S75. Hole and electron analyses of $S_0 \rightarrow S_1$ and $S_0 \rightarrow T_1$ transitions of **NDH** coupled with **PBH** crystal cell.

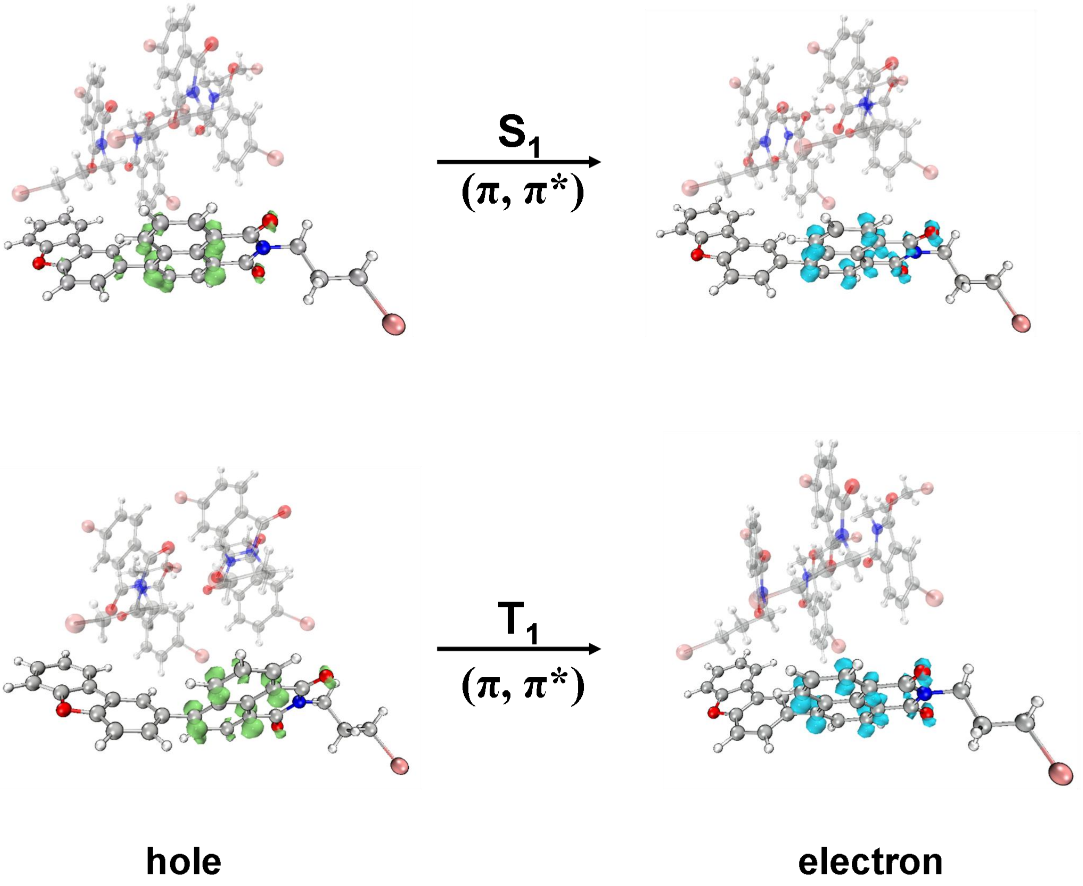

Fig. S76. Hole and electron analyses of $S_0 \rightarrow S_1$ and $S_0 \rightarrow T_1$ transitions of **NDB** coupled with **PBB** crystal cell.

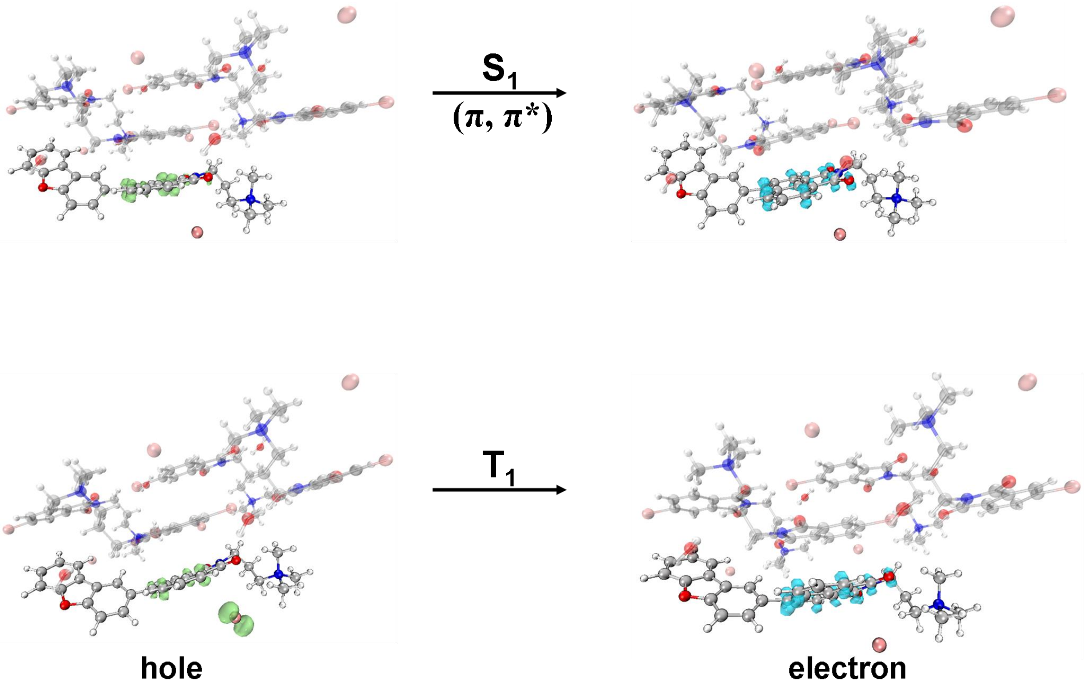

Fig. S77. Hole and electron analyses of $S_0 \rightarrow S_1$ and $S_0 \rightarrow T_1$ transitions of **NDNC** coupled with **PBNC** crystal cell.

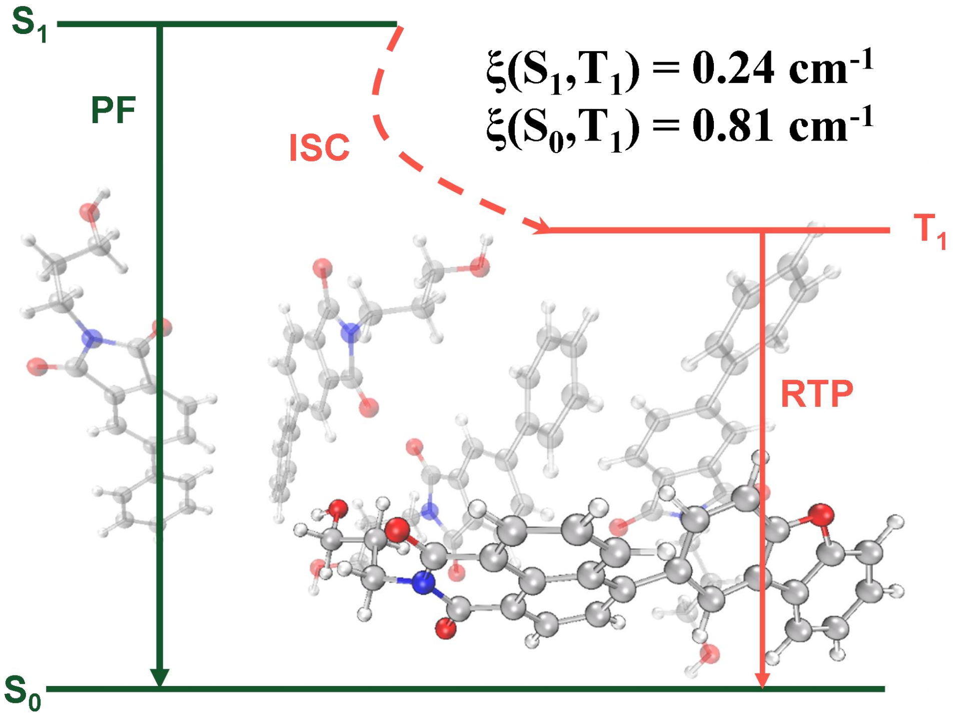

Fig. S78. Diagrams of the calculated energy levels and the SOC constants between singlet and triplet excited states of **NDOH** coupled with **PPOH** crystal cell.

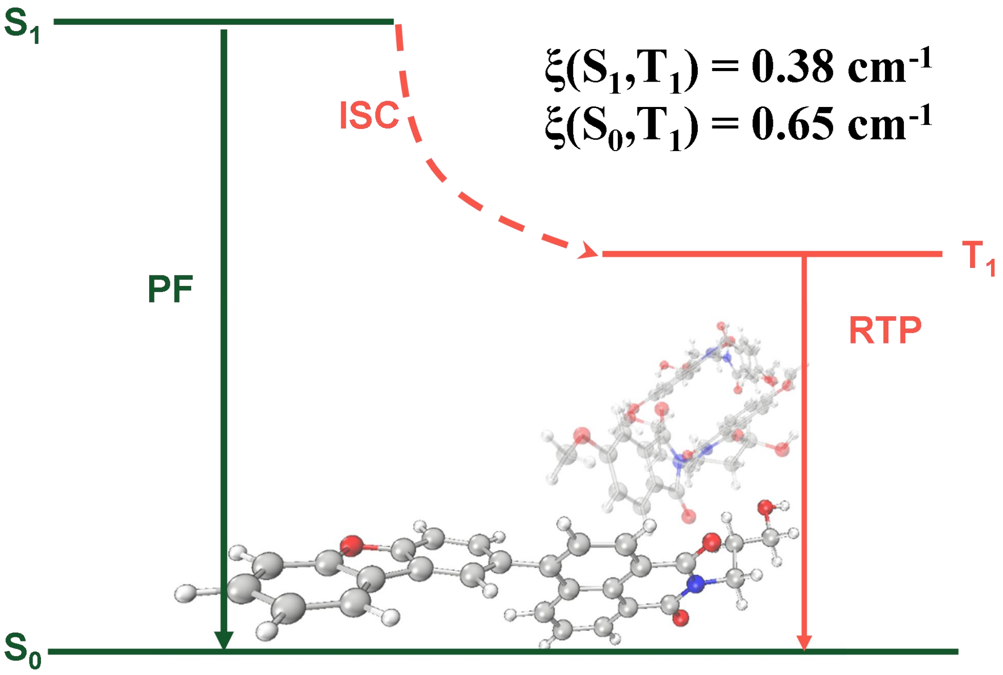

Fig. S79. Diagrams of the calculated energy levels and the SOC constants between singlet and triplet excited states of **NDOH** coupled with **POOH** crystal cell.

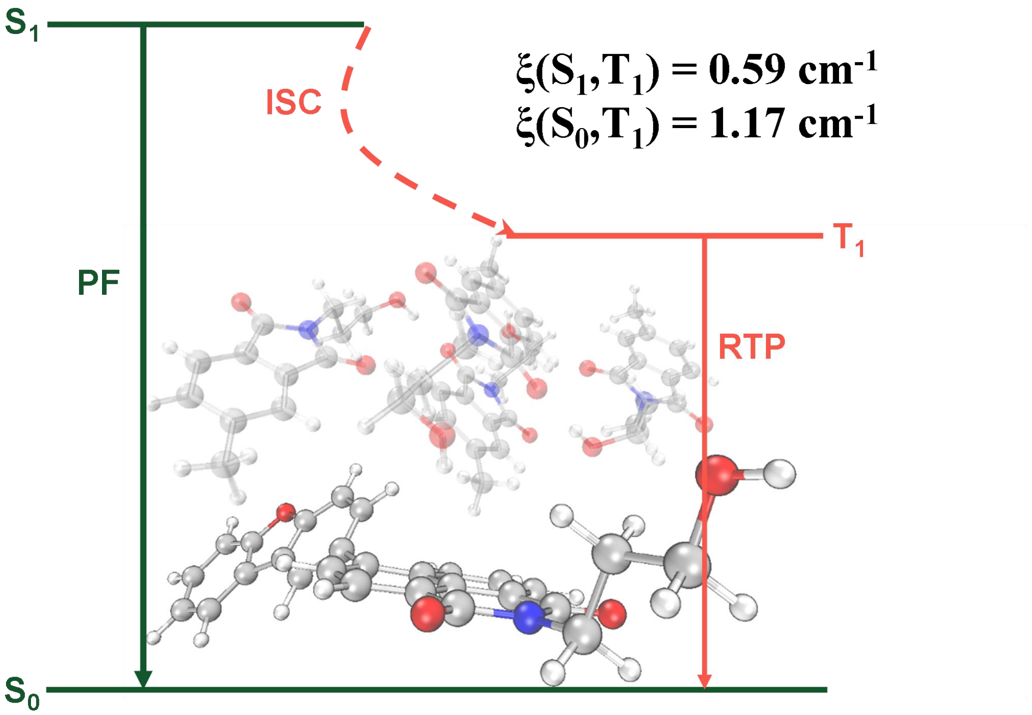

Fig. S80. Diagrams of the calculated energy levels and the SOC constants between singlet and triplet excited states of **NDOH** coupled with **PMOH** crystal cell.

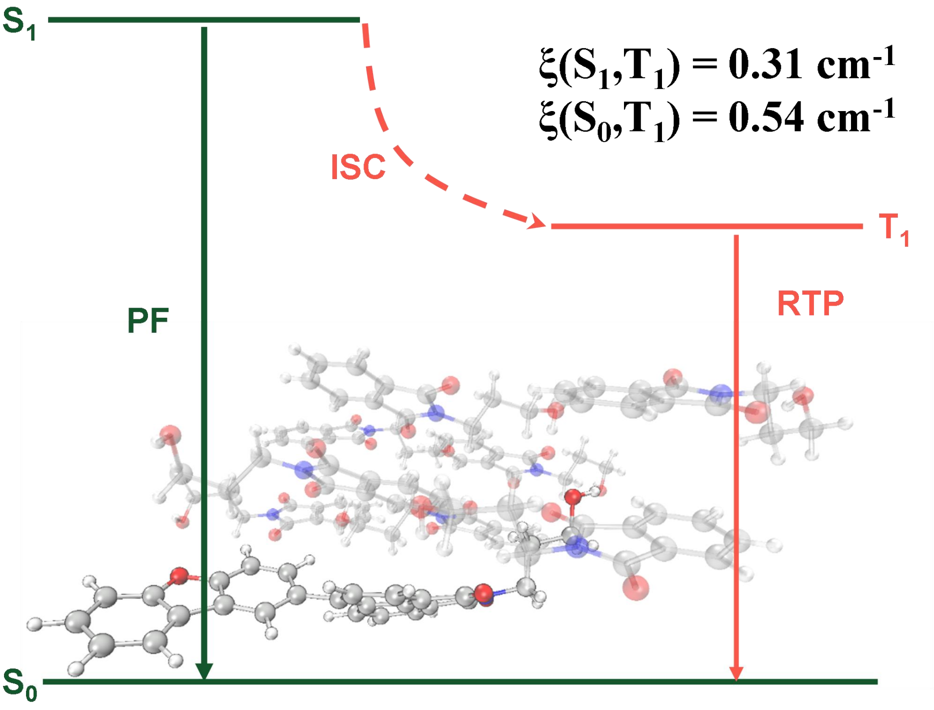

Fig. S81. Diagrams of the calculated energy levels and the SOC constants between singlet and triplet excited states of **NDOH** coupled with **PHOH** crystal cell.

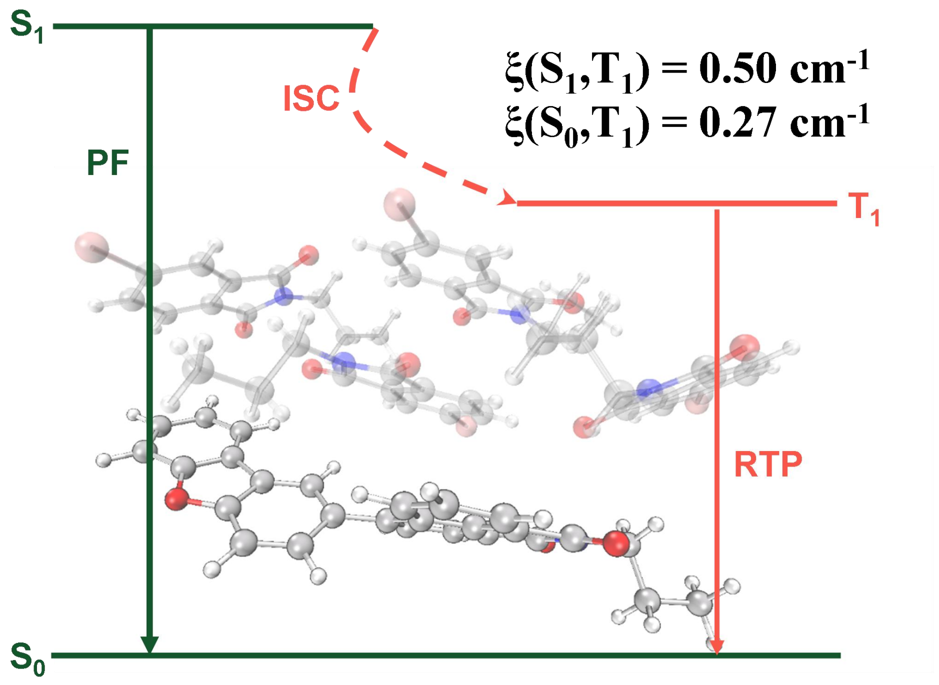

Fig. S82. Diagrams of the calculated energy levels and the SOC constants between singlet and triplet excited states of **NDH** coupled with **PBH** crystal cell.

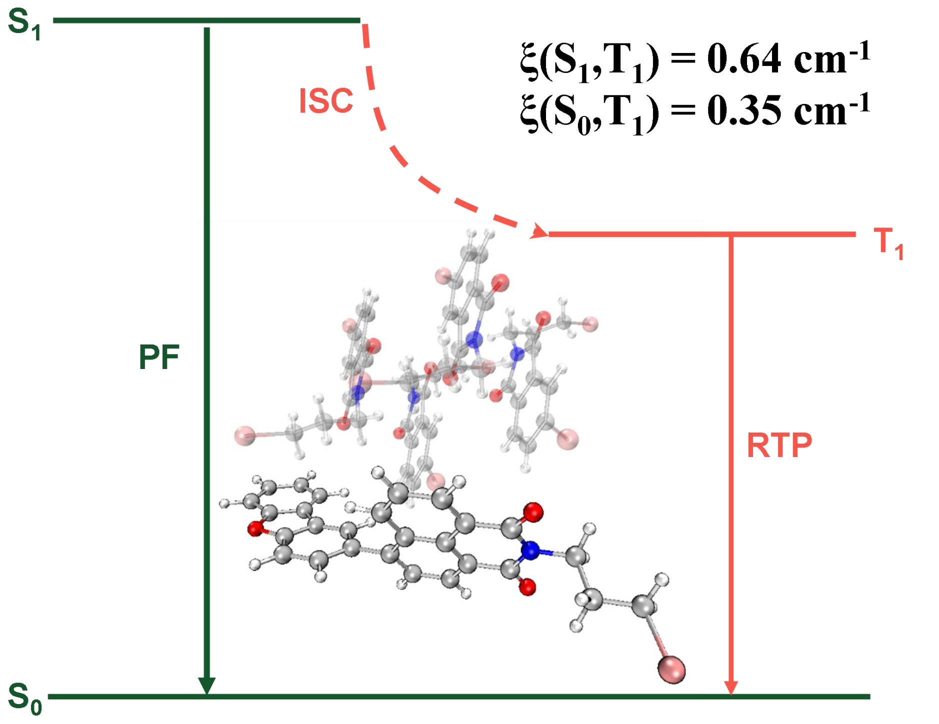

Fig. S83. Diagrams of the calculated energy levels and the SOC constants between singlet and triplet excited states of **NDB** coupled with **PBB** crystal cell.

Photophysical properties of the twenty-two host-guest systems were summarized in Table S2. Lifetimes of host fluorescence and prompt fluorescence of host-guest systems were also measured (Fig. S84-S90). PLQY measurement of host materials demonstrated that smallest PLQY could be observed on **PBNC** crystal (Table S1). However, highest phosphorescence quantum yield could be observed on **NDNC@PBNC** powder, suggesting the main energy transfer process should be Dexter energy transfer since it is independent of the quantum yield of donor, while Förster energy transfer rate depends on the quantum yield of donor. Influences of doping ratios on photophysical properties of **NDOH@PBOH** powder were also investigated. As presented in Fig. S91, with molar ratios between **PBOH** and **NDOH** decreasing from 10000:1 to 1:1, decrease of phosphorescence lifetime was found, which should be ascribed to the quenching of triplet excitons induced by strong intermolecular π–π interactions. Although longer RTP lifetime was observed at the molar ratio of 10000:1 between **PBOH** and **NDOH**, smaller relative intensity of RTP was also found due to the intrinsic distance dependence of Dexter energy transfer (Fig. S92). When **NDOH** was at lower concentration, less guest molecules would get close to host molecules, leading to less efficient TTET.

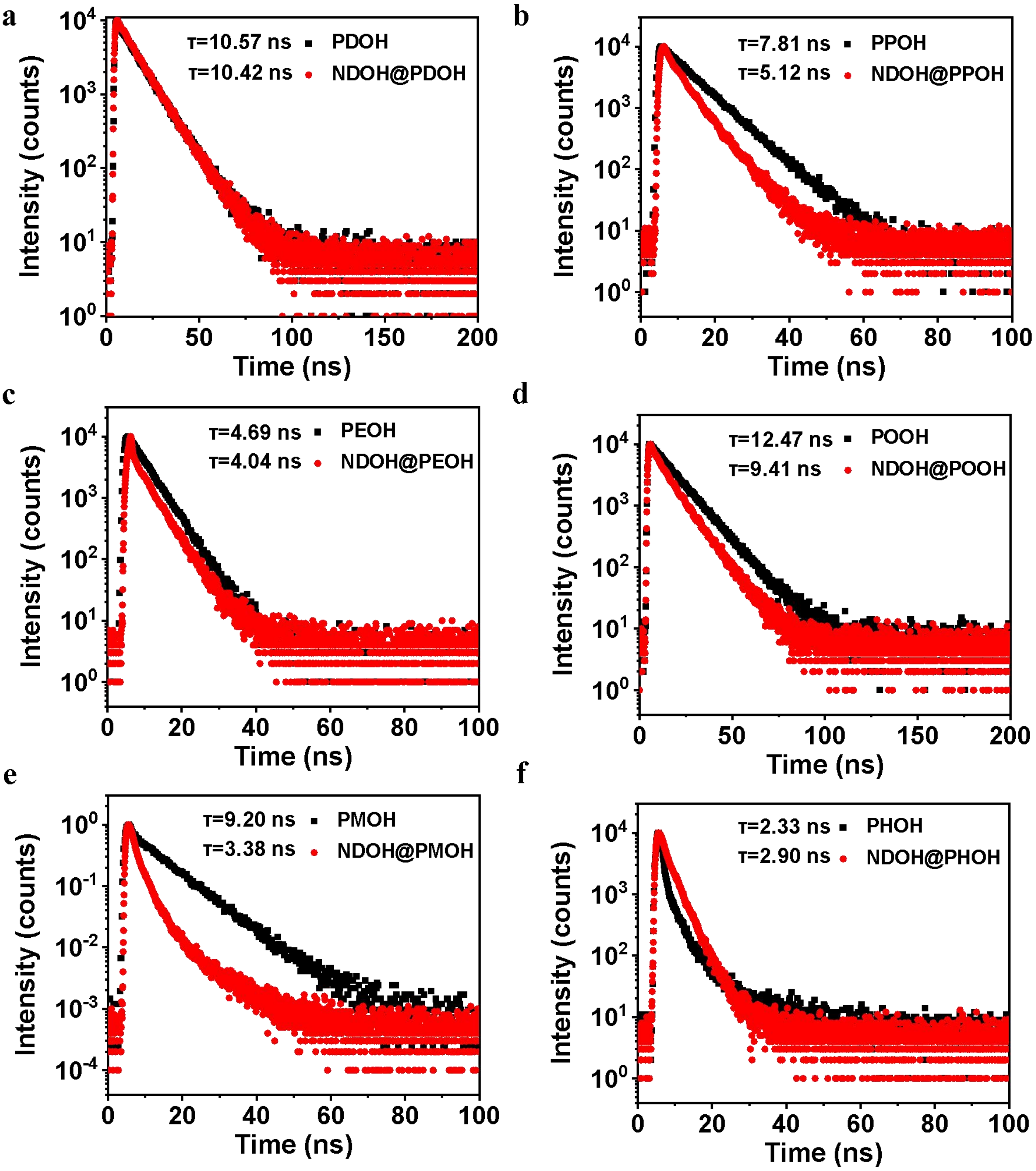

Fig. S84. Decay curves of **PDOH**, **PPOH**, **PEOH**, **POOH**, **PMOH** and **PHOH** monitored at the maximum fluorescence emission wavelengths in the absence and presence of guest molecule **NDOH**.

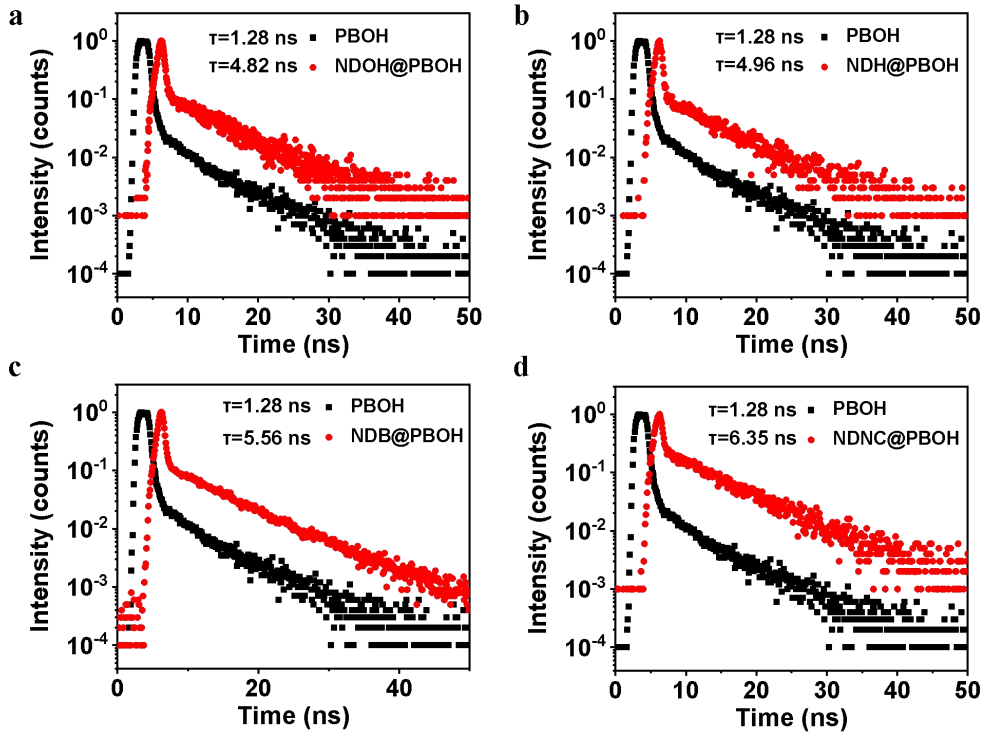

Fig. S85. Decay curves of **PBOH** monitored at the maximum fluorescence emission wavelength in the absence and presence of guest molecule **NDOH**, **NDH**, **NDB** and **NDNC**.

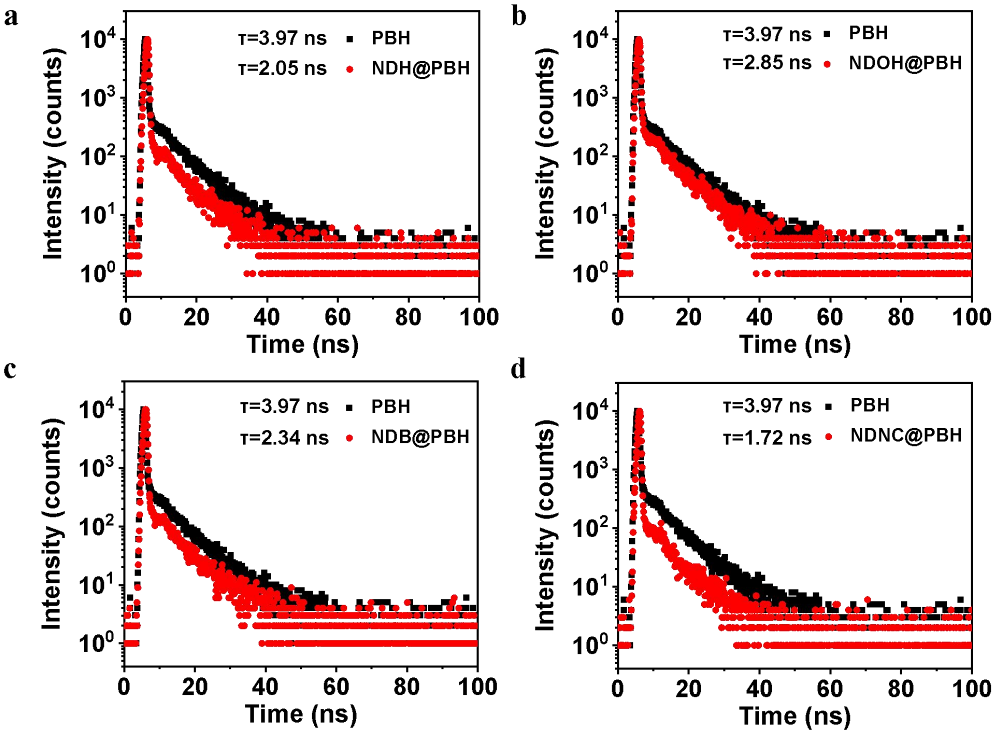

Fig. S86. Decay curves of PBH monitored at the maximum fluorescence emission wavelength in the absence and presence of guest molecule **NDOH**, **NDH**, **NDB** and **NDNC**.

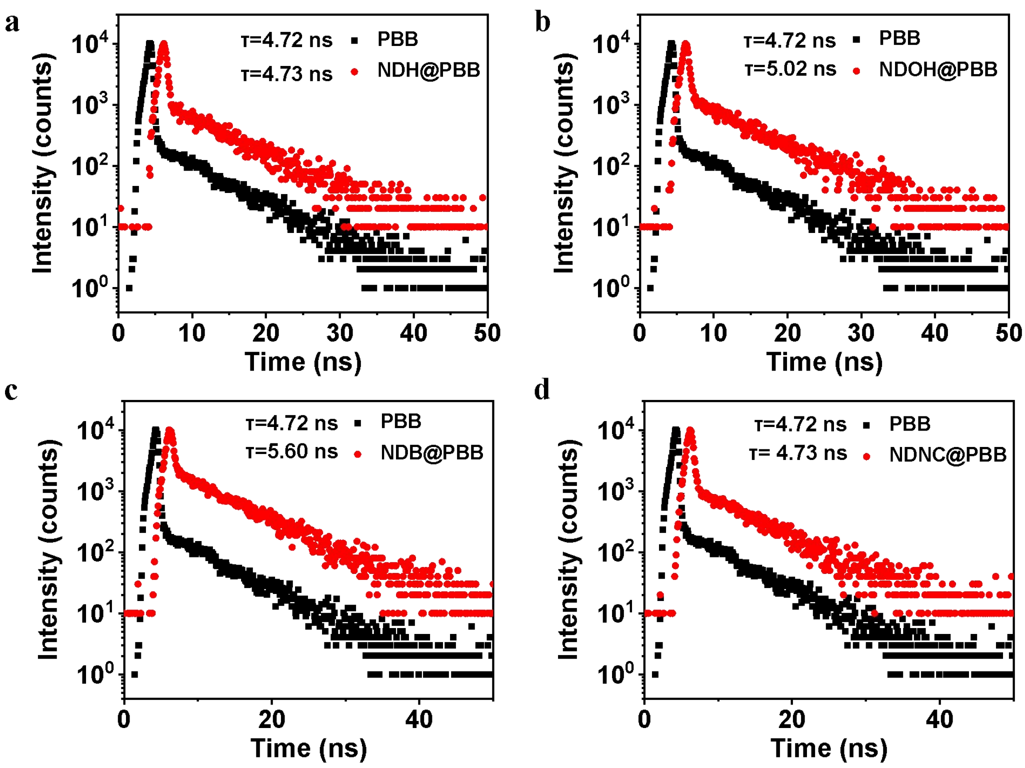

Fig. S87. Decay curves of **PBB** monitored at the maximum fluorescence emission wavelength in the absence and presence of guest molecule **NDOH**, **NDH**, **NDB** and **NDNC**.

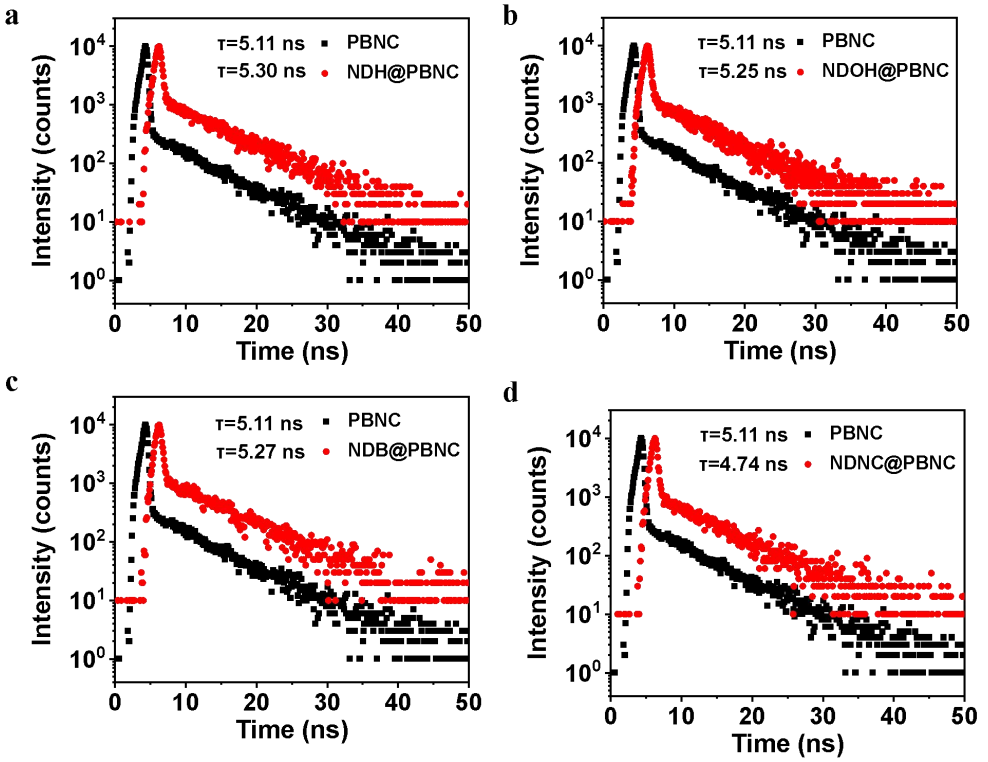

Fig. S88. Decay curves of **PBNC** monitored at the maximum fluorescence emission wavelength in the absence and presence of guest molecule **NDOH**, **NDH**, **NDB** and **NDNC**.

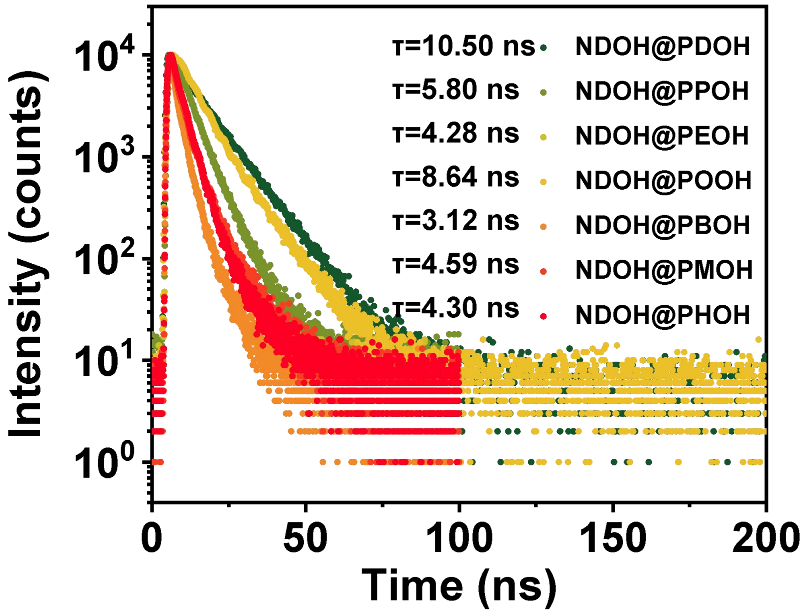

Fig. S89. Decay curves of **NDOH** doped in **PDOH**, **PPOH**, **PEOH**, **POOH**, **PBOH**, **PMOH** and **PHOH** monitored at maximum fluorescence emission wavelengths.

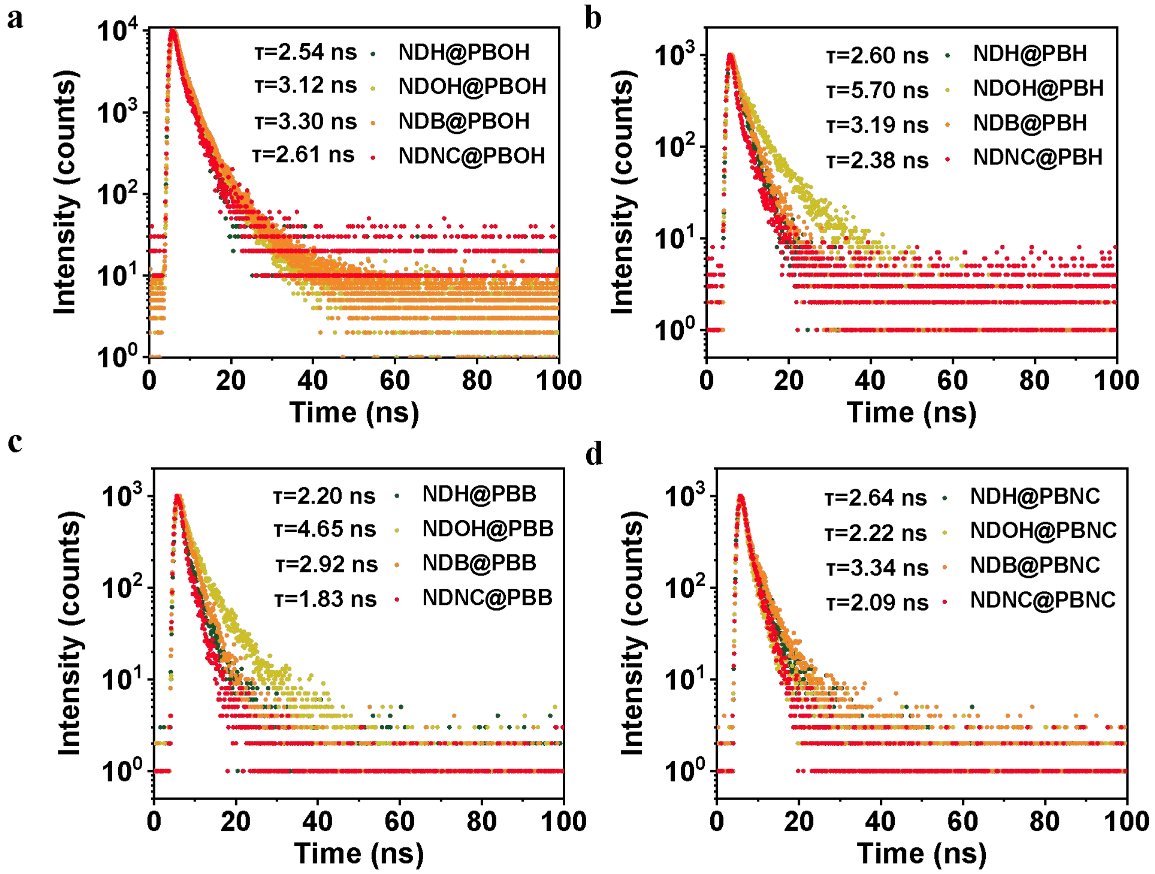

Fig. S90. Decay curves of **NDOH**, **NDH**, **NDB** and **NDNC** doped in **PBOH**, **PBH**, **PBB** and **PBNC** monitored at maximum fluorescence emission wavelengths.

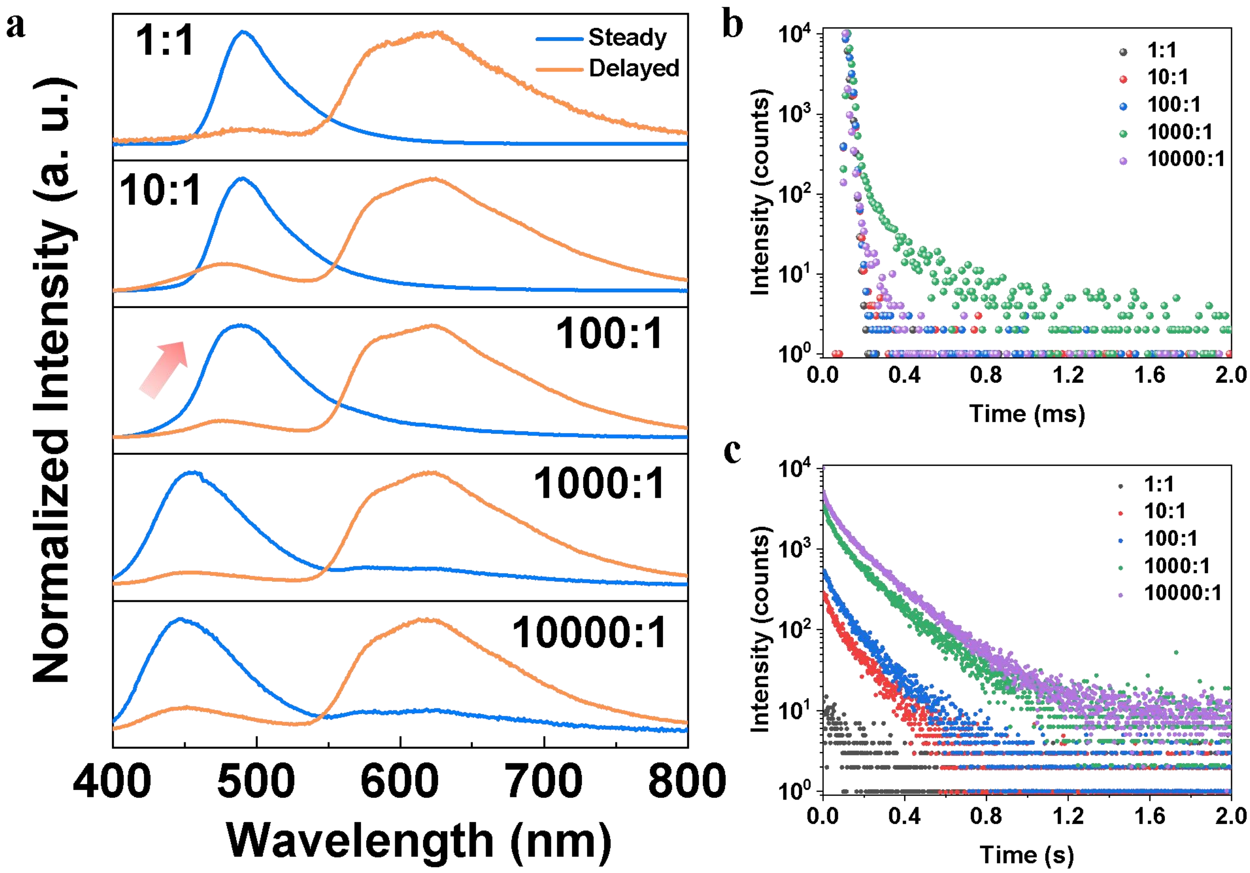

Fig. S91. a) Steady spectra and delayed spectra of **NDOH@PBOH** powder with different molar ratios (λ_{ex}=365 nm, delayed time = 8 ms). b) Decay curves of **NDOH@PBOH** powder with different molar ratios monitored at maximum TADF emission wavelengths. c) Decay curves of **NDOH@PBOH** powder with different molar ratios monitored at maximum RTP emission wavelengths.

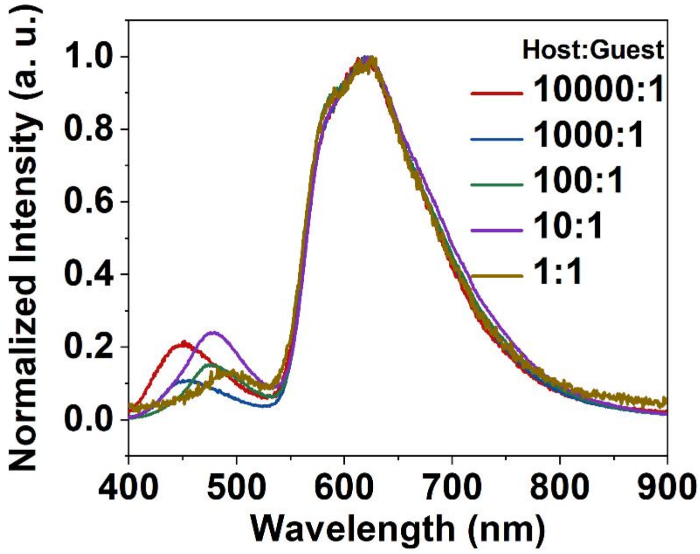

Fig. S92. Delayed spectra of **NDOH@PBOH** powder with different molar ratios (λ_{ex}=365 nm, delayed time = 8 ms).

Table S1. The photophysical data of the host materials at room temperature.

Sample	λ_F^a (nm)	λ_P^b (nm)	PLQY (%)	τ_F^c (ns)	τ_P^d (ms)
**PDOH**	450	615	41.2	10.57	0.07
**PPOH**	405	605	9.2	7.81	3.92
**PEOH**	405	596	40.9	4.69	0.42
**POOH**	403	587	30.2	12.47	10.69
**PBOH**	404	580	0.9	1.28	1.23
**PMOH**	395	571	4.8	9.20	10.40
**PHOH**	405	565	2.9	2.33	2.40
**PBH**	386	568	4.0	3.97	5.38
**PBB**	385	563	1.0	4.72	0.99
**PBNC**	385	561	<0.1	5.11	33.39

a Maximum fluorescence emission wavelength. ***b*** Maximum phosphorescence emission wavelength from T_1. ***c*** Fluorescence lifetime monitored at λ_F. ***d*** Phosphorescence lifetime monitored at λ_P.

Table S2. The photophysical data of the host-guest systems at room temperature.

Sample	λ_{TADF}[a] (nm)	λ_P (nm)	I_{TADF}/I_P	$PLQY_P$[b] (%)	τ_F (ns)	τ_{TADF}[c] (μs)	τ_P (ms)	CIE coordinate[d]
**NDOH@PDOH**	460	602	20.0	1.7	10.50	12.25	55.79	(0.17,0.24)
**NDOH@PPOH**	455	584	7.7	1.4	5.80	11.86	59.40	(0.20,0.18)
**NDOH@PEOH**	449	584	6.2	0.4	4.28	13.53	75.39	(0.21,0.18)
**NDOH@POOH**	460	610	0.6	1.6	8.64	11.61	127.10	(0.42,0.27)
**NDOH@PBOH**	455	614	0.2	3.9	3.12	41.93	159.04	(0.53,0.38)
**NDOH@PMOH**	458	580	0.1	3.8	4.59	104.30	167.58	(0.55,0.39)
**NDOH@PHOH**	456	589	0.1	2.1	4.30	15.51	165.02	(0.56,0.38)
**NDH@PBOH**	450	620	0.2	2.1	2.54	44.22	138.32	(0.53,0.38)
**NDB@PBOH**	455	612	0.05	4.9	3.30	42.28	104.97	(0.55,0.43)
**NDNC@PBOH**	463[e]	611	0	0.7	2.61	184.95	83.78	(0.56,0.43)
**NDH@PBH**	440	634	0.09	2.6	2.60	217.02	142.45	(0.56,0.38)
**NDOH@PBH**	446	630	0.09	1.9	5.70	113.46	129.89	(0.57,0.39)
**NDB@PBH**	455	585	0.1	1.1	3.19	333.32	113.56	(0.55,0.58)
**NDNC@PBH**	466	577	0.1	1.3	2.38	465.19	41.64	(0.46,0.44)
**NDH@PBB**	449	622	0.1	1.5	2.20	82.47	78.56	(0.53,0.41)
**NDOH@PBB**	460	590	0.04	2.0	4.65	213.80	102.09	(0.55,0.43)
**NDB@PBB**	445	590	0.06	5.1	2.92	325.36	105.81	(0.58,0.42)
**NDNC@PBB**	469[e]	588	0	1.0	1.83	215.30	51.17	(0.56,0.43)
**NDH@PBNC**	462[e]	586	0	0.6	2.64	-	33.76	(0.47,0.44)
**NDOH@PBNC**	467[e]	608	0	3.9	2.22	-	36.21	(0.51,0.46)
**NDB@PBNC**	463[e]	590	0	5.0	3.34	-	33.86	(0.53,0.44)
**NDNC@PBNC**	449[e]	599	0	13.9	2.09	-	49.17	(0.55,0.41)

a Maximum TADF emission wavelength. ***b*** Phosphorescence PLQY. ***c*** TADF lifetime monitored at λ_{TADF}. ***d*** Afterglow CIE coordinates calculated based on the delayed spectra at 8 ms. ***e*** Emission wavelength determined from the steady spectra.

Table S3. Structural data of single crystals of **PPOH**, **POOH**, **PMOH** and **PHOH**.

**Name**	**PPOH**	**POOH**	**PMOH**	**PHOH**
Formula	$C_{17}H_{15}NO_3$	$C_{12}H_{13}NO_4$	$C_{12}H_{13}NO_3$	$C_{11}H_{11}NO_3$
Wavelength (Å)	0.71073	0.71073	0.71073	0.71073
Space Group	$P2_1/c$	$P2_1/n$	P-1	$P2_1/c$
Cell Lengths (Å)	a = 7.6498(5)<br>b = 23.7865(11)<br>c = 7.9577(5)	a = 9.0089(7)<br>b = 5.0146(4)<br>c = 24.1445(17)	a = 6.863(1)<br>b = 10.0372(7)<br>c = 15.4632(12)	a = 12.1872(13)<br>b = 7.3591(9)<br>c = 21.825(2)
Cell Angles (°)	alpha = 90<br>beta = 110.975(7)<br>gamma = 90	alpha = 90<br>beta = 94.336(7)<br>gamma = 90	alpha=97.142(6)<br>beta = 91.741(9)<br>gamma = 90.403(9)	alpha = 90<br>beta = 94.137(10)<br>gamma = 90
Cell Volume ($Å^3$)	1352.05(15)	1087.63(14)	1056.37(19)	1952.3(4)
Z	4	4	8	8
Density (g cm^{-3})	1.382	1.437	1.378	1.396
F (000)	592.0	496.0	464.0	864.0
CCDC Number	2214258	2214259	2214261	2214262

Table S4. Structural data of single crystals of **PBOH**, **PBH, PBB** and **PBNC**.

**Name**	**PBOH**	**PBH**	**PBB**	**PBNC**
Formula	$C_{11}H_{10}BrNO_3$	$C_{11}H_{10}BrNO_2$	$C_{11}H_9Br_2NO_2$	$C_{14}H_{19}Br_2N_2O_{2.5}$
Wavelength (Å)	0.71073	0.71073	1.54184	1.54184
Space Group	P-1	$P2_1/n$	P-1	P-1
Cell Lengths (Å)	a = 7.0443(6)<br>b = 10.1741(8)<br>c = 15.3812(13)	a = 11.7530(7)<br>b = 6.9229(4)<br>c = 13.4976(7)	a = 5.6358(2)<br>b = 8.6630(3)<br>c = 12.5502(5)	a = 7.3001(10)<br>b = 10.0479(12)<br>c = 13.2986(16)
Cell Angles (°)	alpha=97.434(7)<br>beta=92.256(7)<br>gamma=90.119(7)	alpha=90<br>beta=106.332(6)<br>gamma=90	alpha=97.843(3)<br>beta=102.853(3)<br>gamma=93.616(3)	alpha=68.483(11)<br>beta=77.213(11)<br>gamma=79.429(11)
Cell Volume ($Å^3$)	1092.22(16)	1053.91(11)	589.02(4)	879.3(2)
Z	4	4	2	2
Density (g cm^{-3})	1.728	1.690	1.957	1.568
F (000)	568.0	536.0	336.0	414.0
CCDC Number	2214260	2214263	2214264	2214265

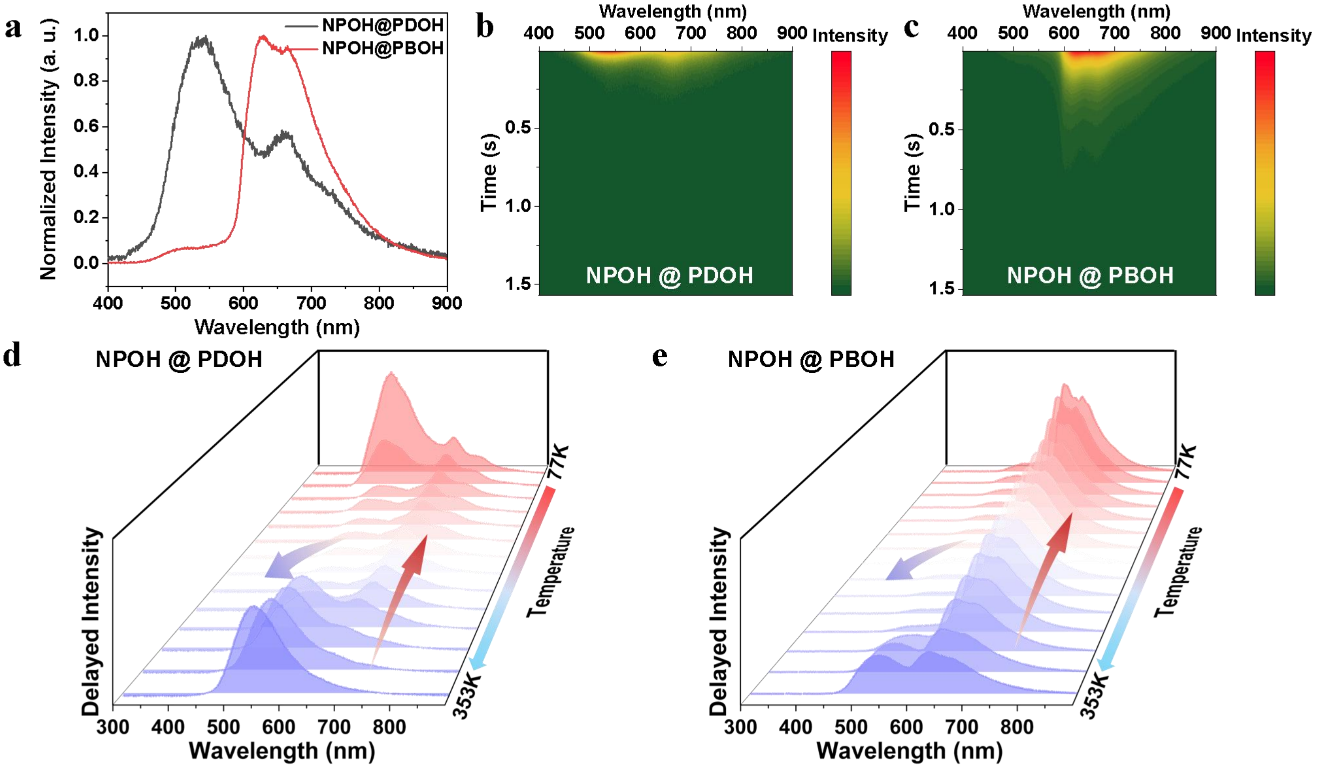

Fig. S93. a) The delayed emission spectra of **NPOH@PDOH** and **NPOH@PBOH** powder. (λ_{ex}=365 nm, delayed time = 8 ms). b-c) The time-resolved delayed spectra of **NPOH@PDOH** (b) and **NPOH@PBOH** (c) powder. (λ_{ex}=365 nm, at 298 K). d-e) The delayed emission spectra of **NPOH@PDOH** (d) and **NPOH@PBOH** (e) powder from 77 to 353 K. (λ_{ex}=365 nm, delayed time = 8 ms).

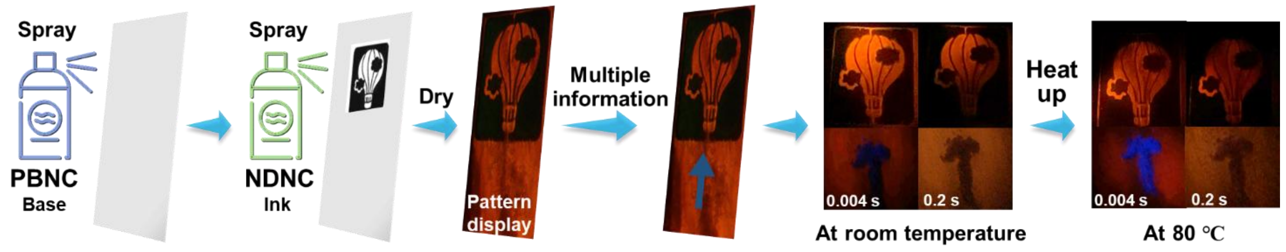

Fig. S94. The schematic diagram of information encryption by spraying. First, ethanol solution of **PBNC** was sprayed on the paper to be the base. Then, ethanol solution of **NDNC** was sprayed to interact with **PBNC**. Lastly, **NDOH@POOH** was placed below the pattern.

The schematic diagram of information encryption was presented in Fig. S94. First, ethanol solution of **PBNC** was sprayed on the paper to act as the base. Then, ethanol solution of **NDNC** was sprayed to interact with **PBNC**. After dried, the painted pattern exhibited bright orange afterglow. Lastly, **NDOH@POOH** was placed below the pattern. At room temperature, due to the weak TADF and orange RTP of **NDOH@POOH**, the arrow could not be observed clearly. At 80 °C, strong TADF of **NDOH@POOH** made the arrow observed clearly.

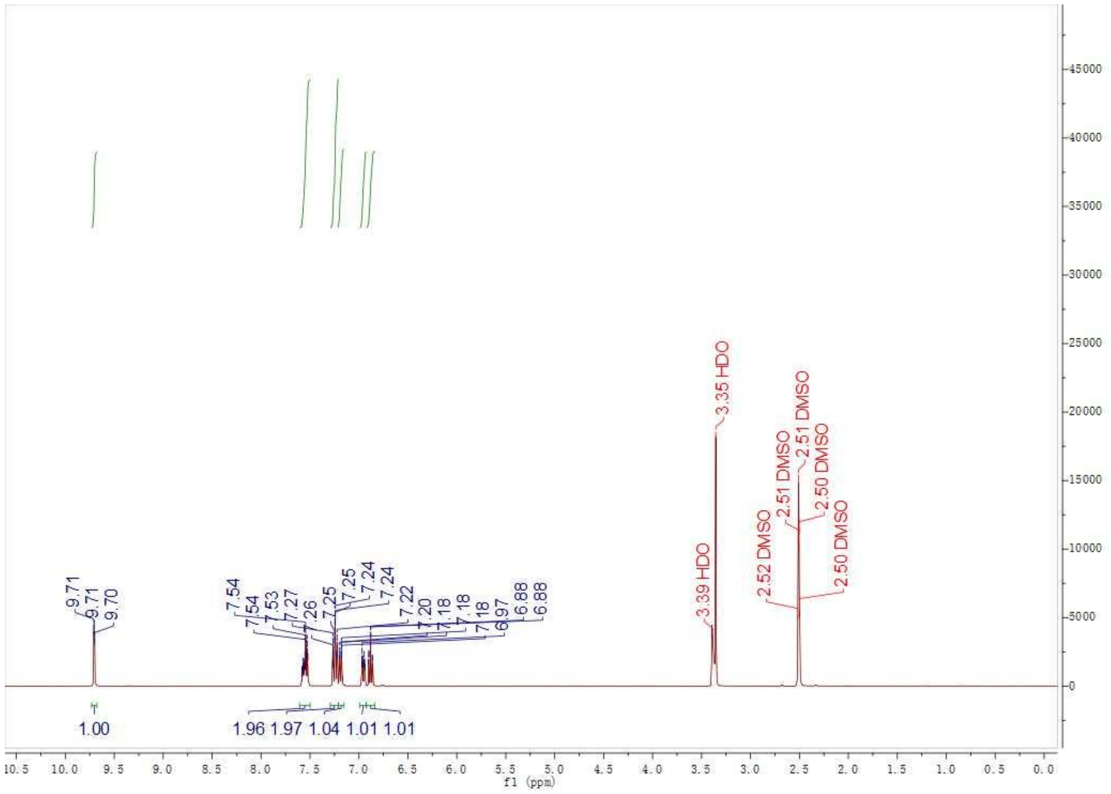

Fig. S95. ^{1}H NMR spectrum of **BP** (in DMSO-d_6).

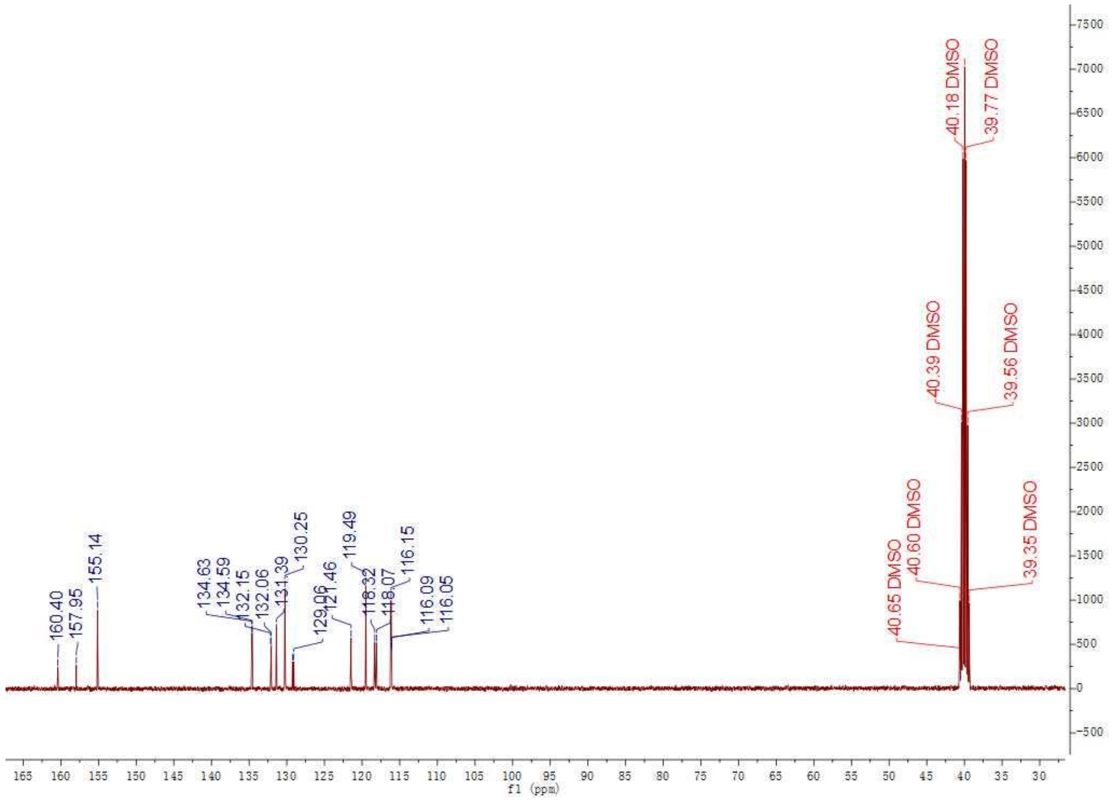

Fig. S96. ^{13}C NMR spectrum of **BP** (in DMSO-d_6).

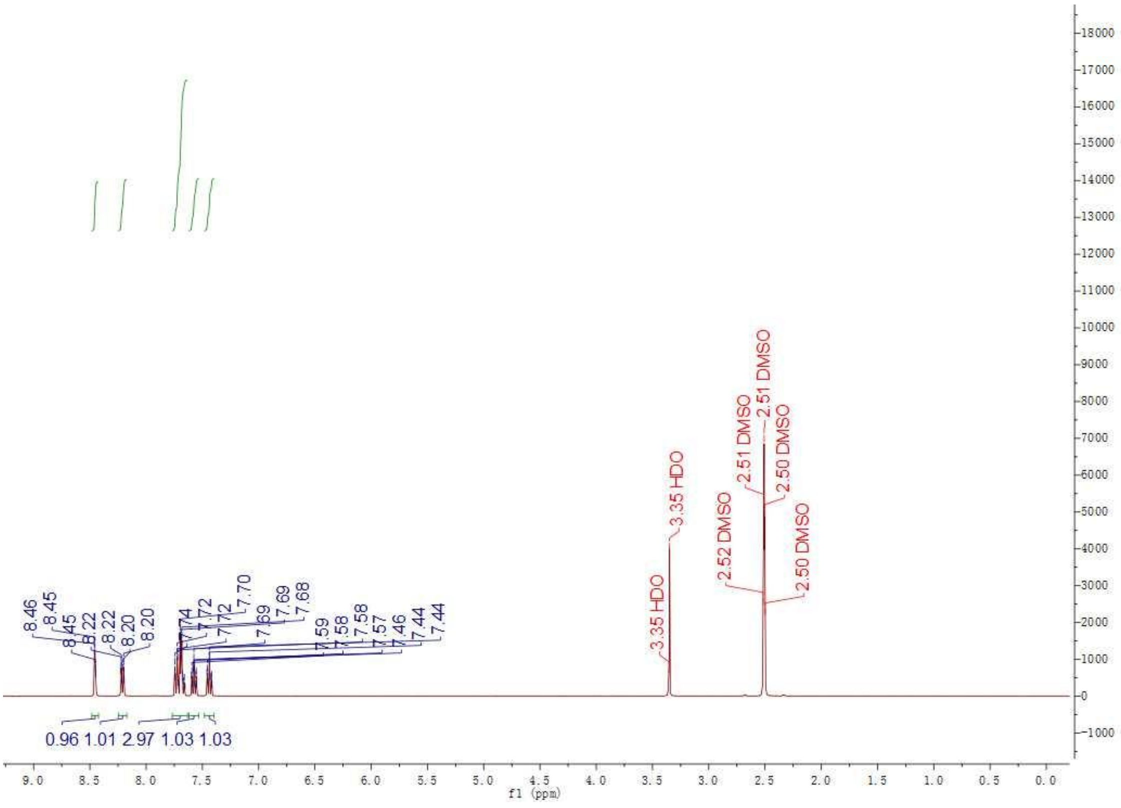

Fig. S97. ^{1}H NMR spectrum of **DBF** (in DMSO-d_6).

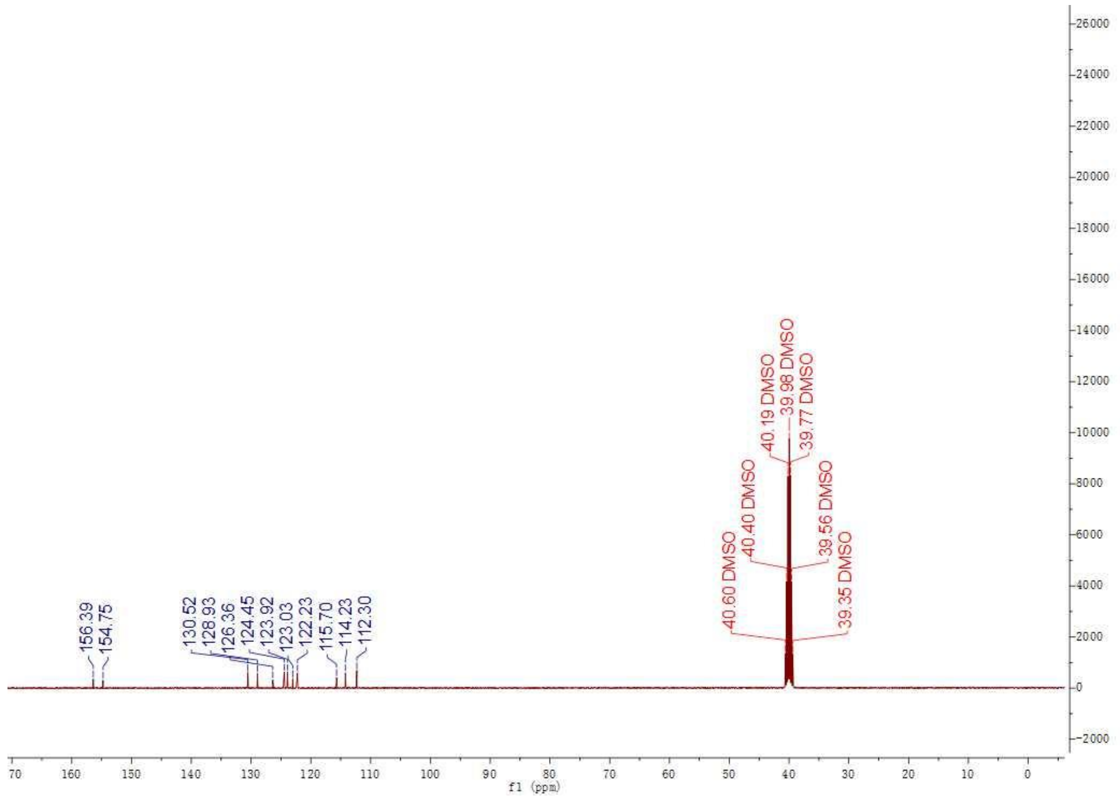

Fig. S98. ${}^{13}C$ NMR spectrum of **DBF** (in DMSO-d_6).

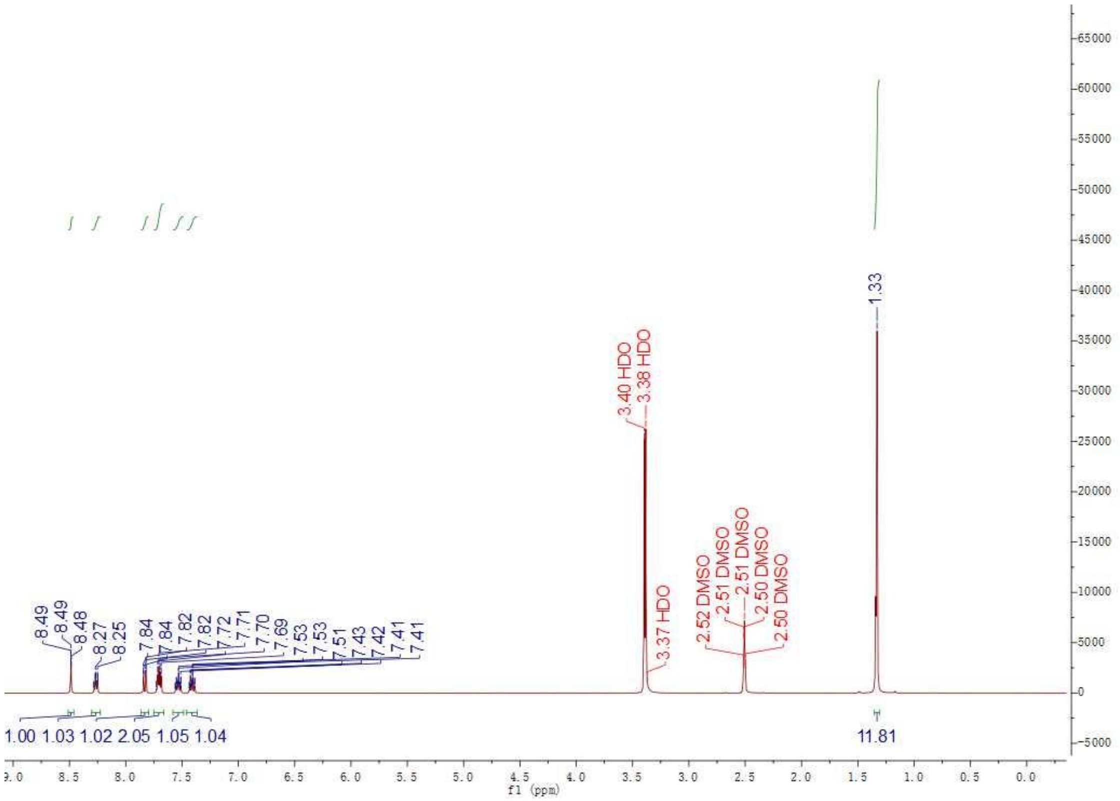

Fig. S99. ${}^{1}H$ NMR spectrum of **DBF-Bpin** (in DMSO-d_6).

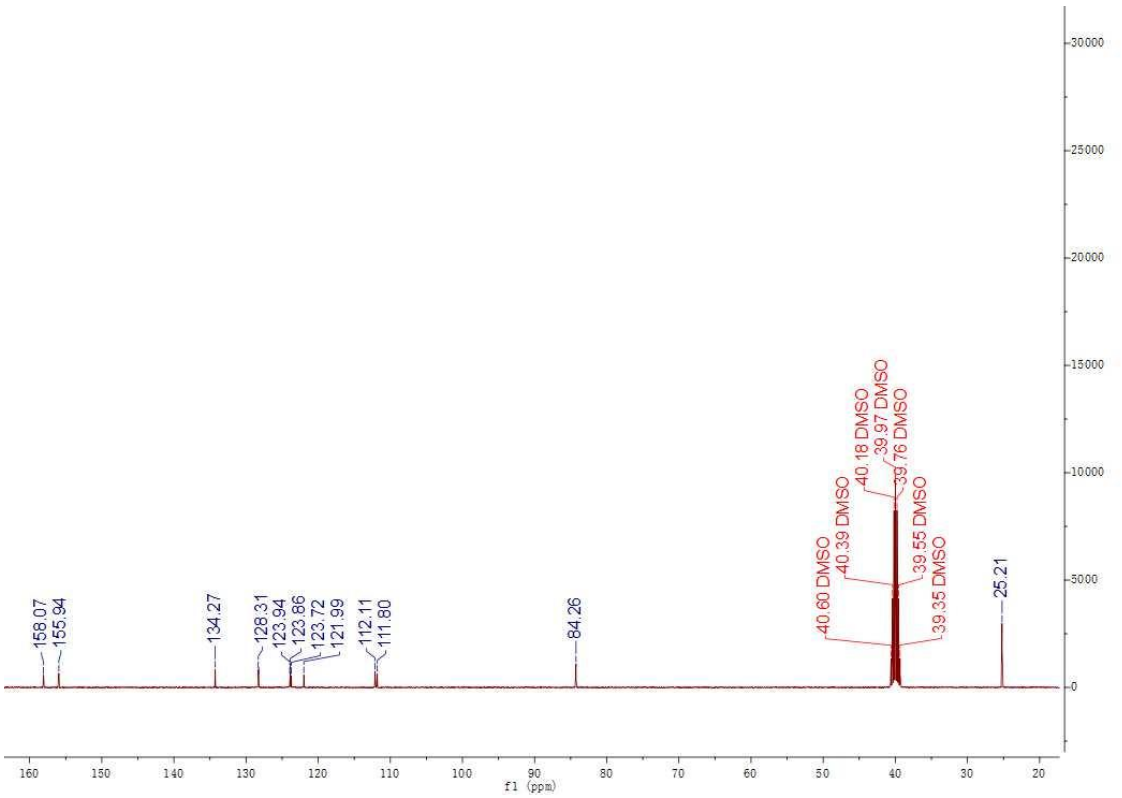

Fig. S100. ^{13}C NMR spectrum of **DBF-Bpin** (in DMSO-d_6).

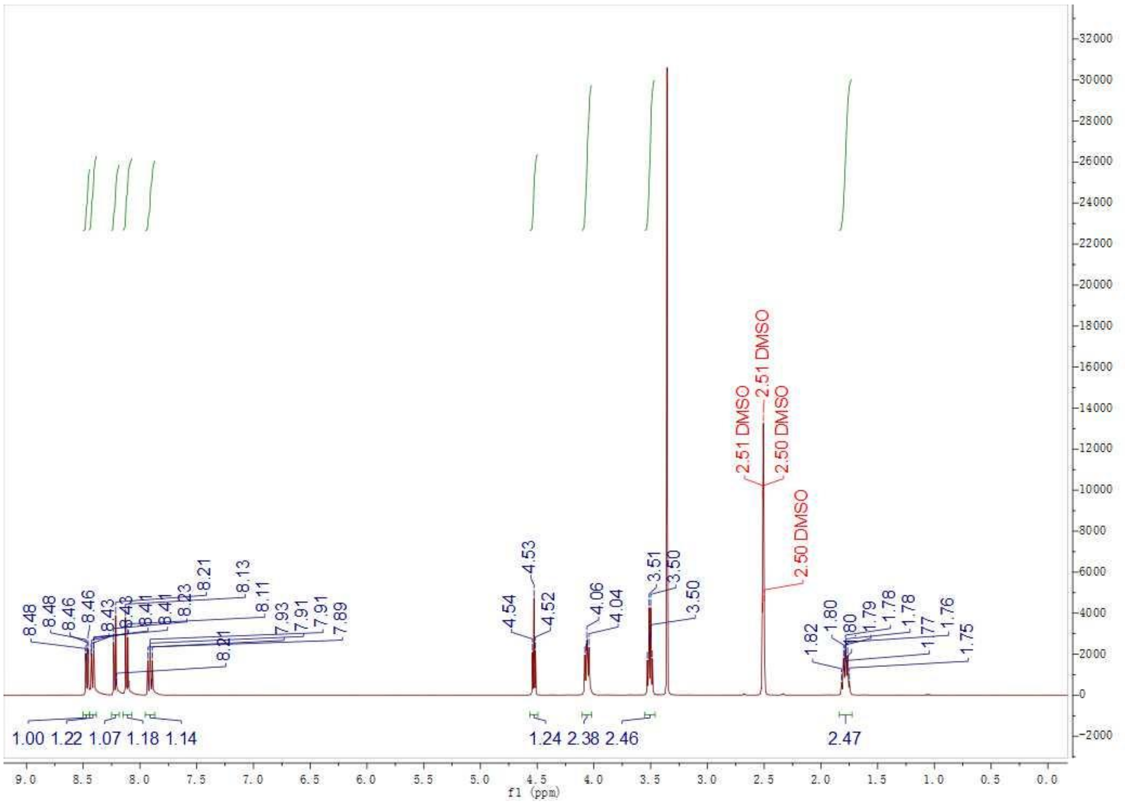

Fig. S101. ^{1}H NMR spectrum of **NBOH** (in DMSO-d_6).

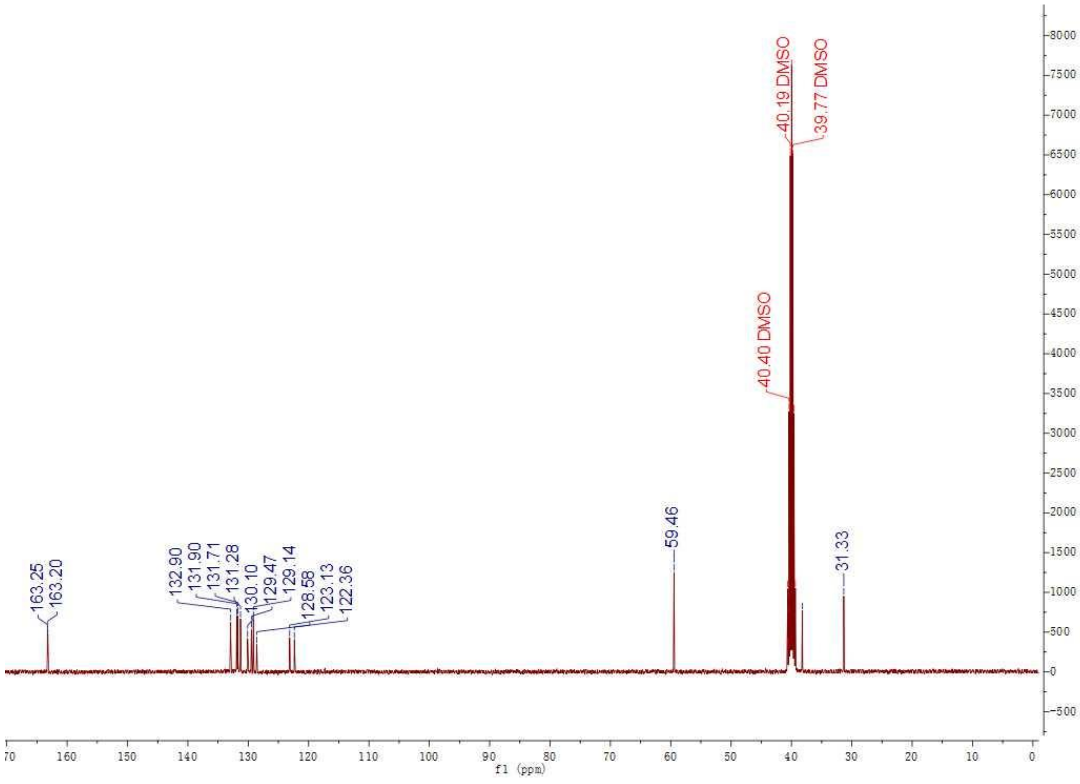

Fig. S102. ^{13}C NMR spectrum of **NBOH** (in DMSO-d_6).

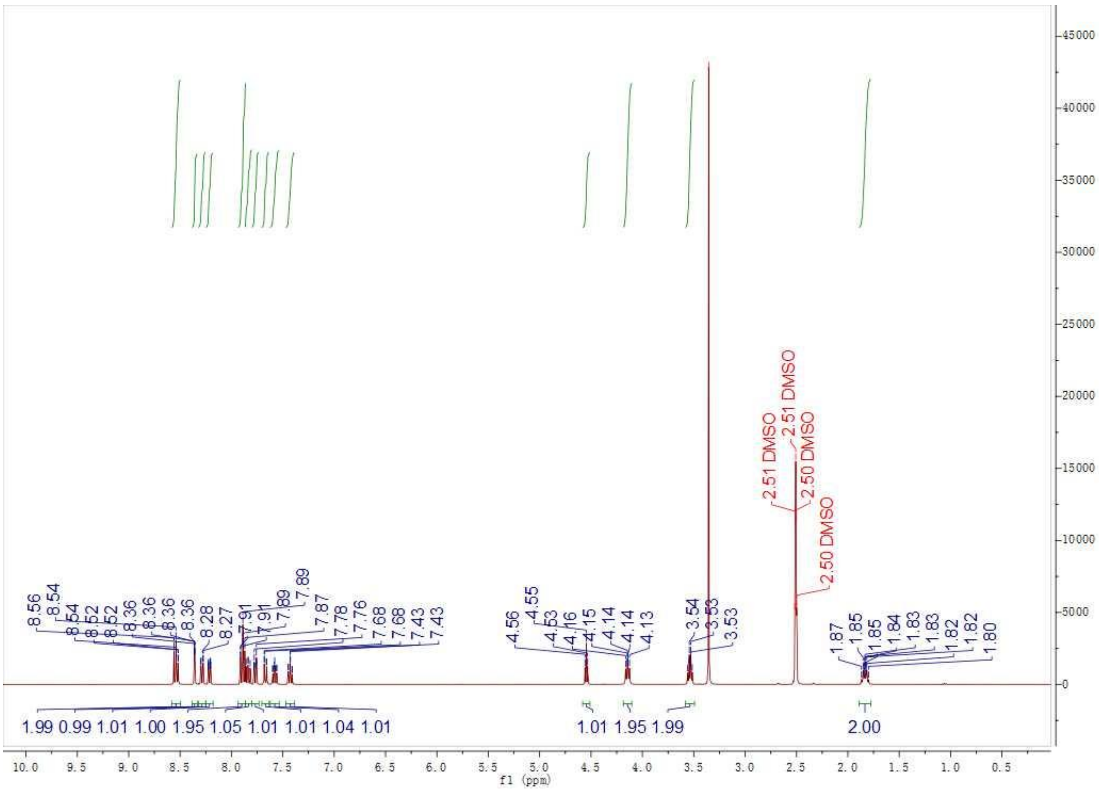

Fig. S103. ^{1}H NMR spectrum of **NDOH** (in DMSO-d_6).

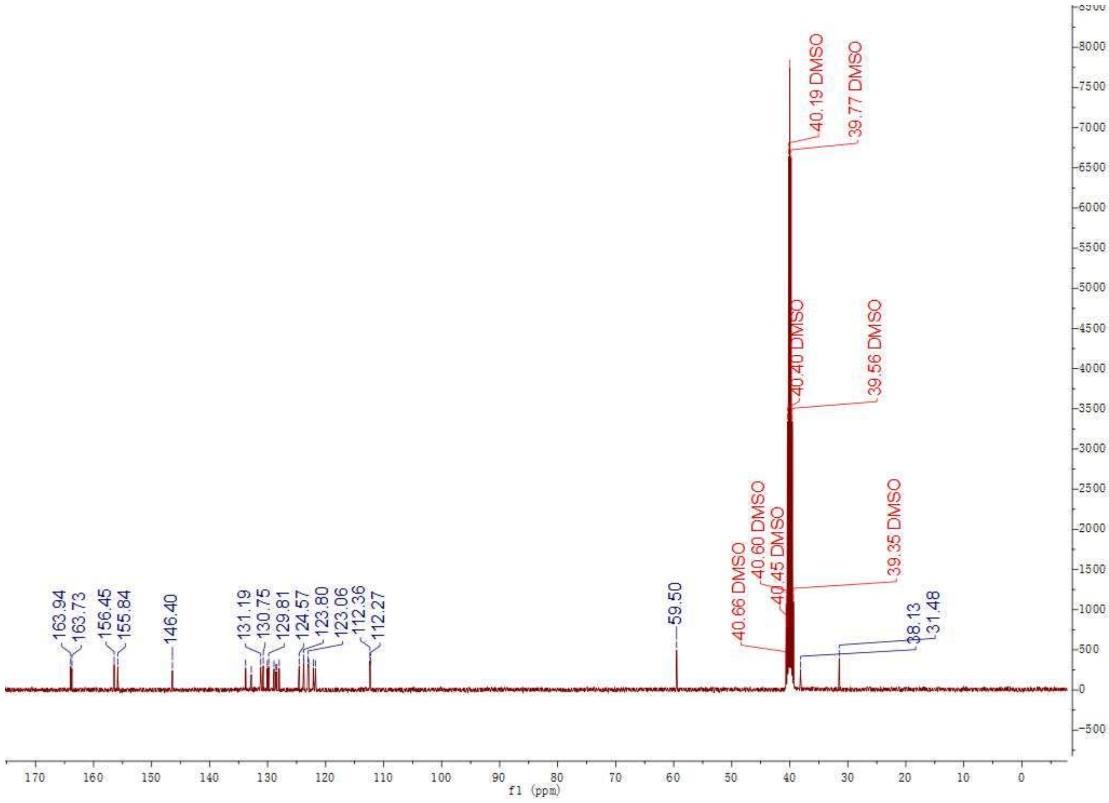

Fig. S104. ^{13}C NMR spectrum of **NDOH** (in DMSO-d_6).

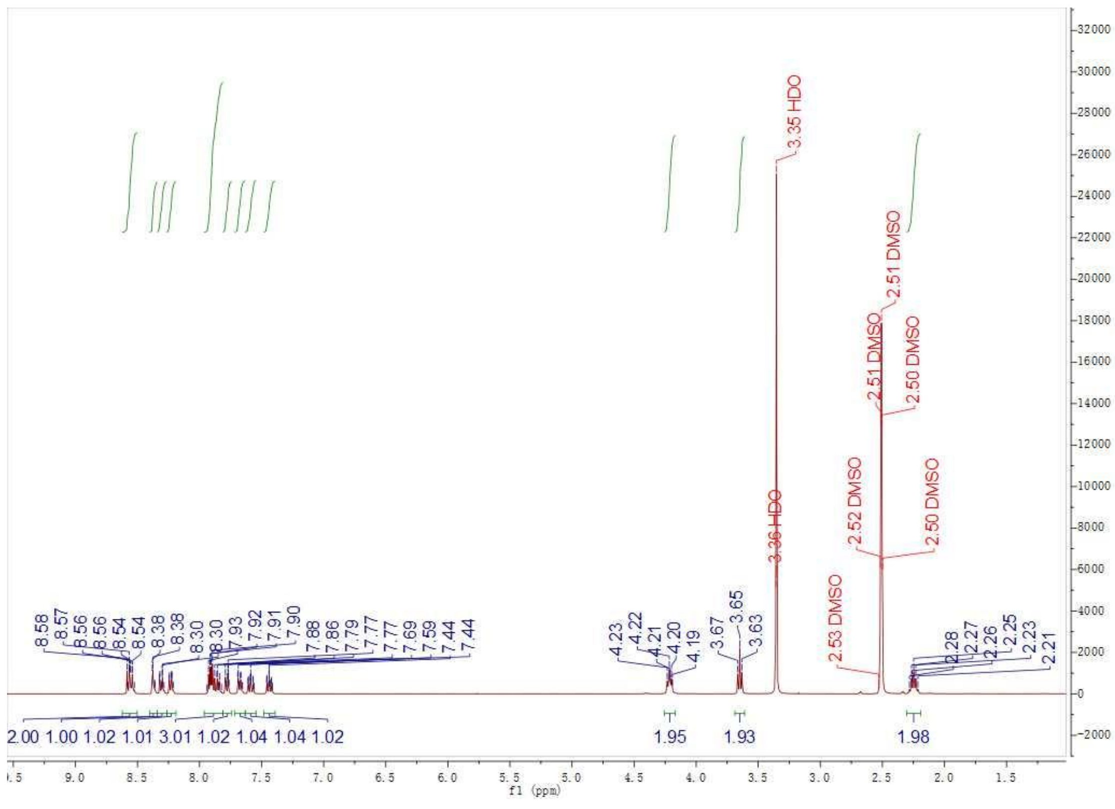

Fig. S105. ^{1}H NMR spectrum of **NDB** (in DMSO-d_6).

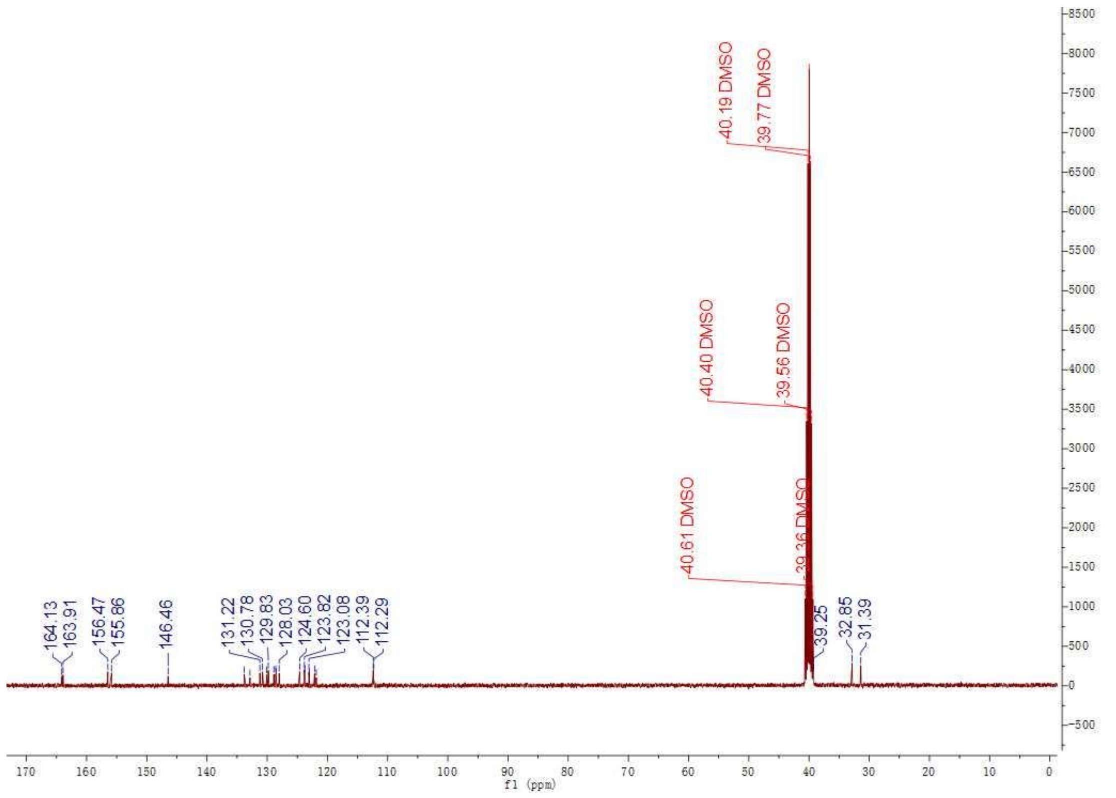

Fig. S106. ^{13}C NMR spectrum of **NDB** (in DMSO-d_6).

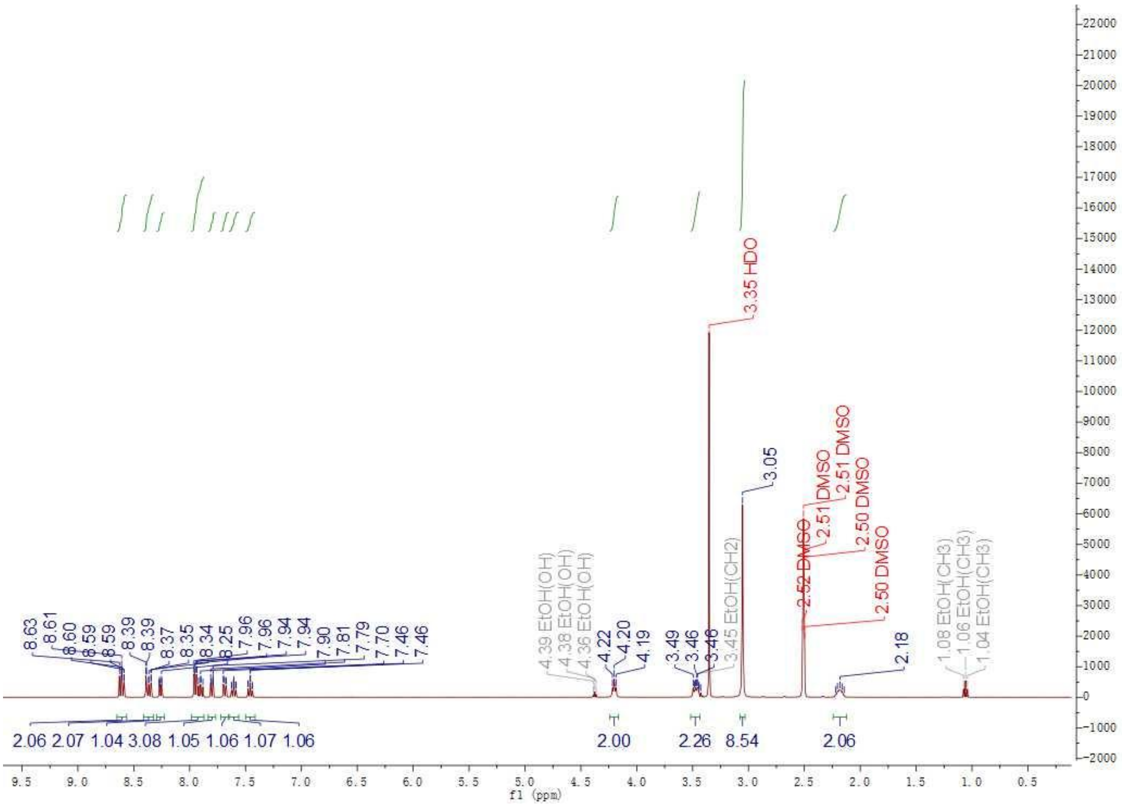

Fig. S107. ^{1}H NMR spectrum of **NDNC** (in DMSO-d_6).

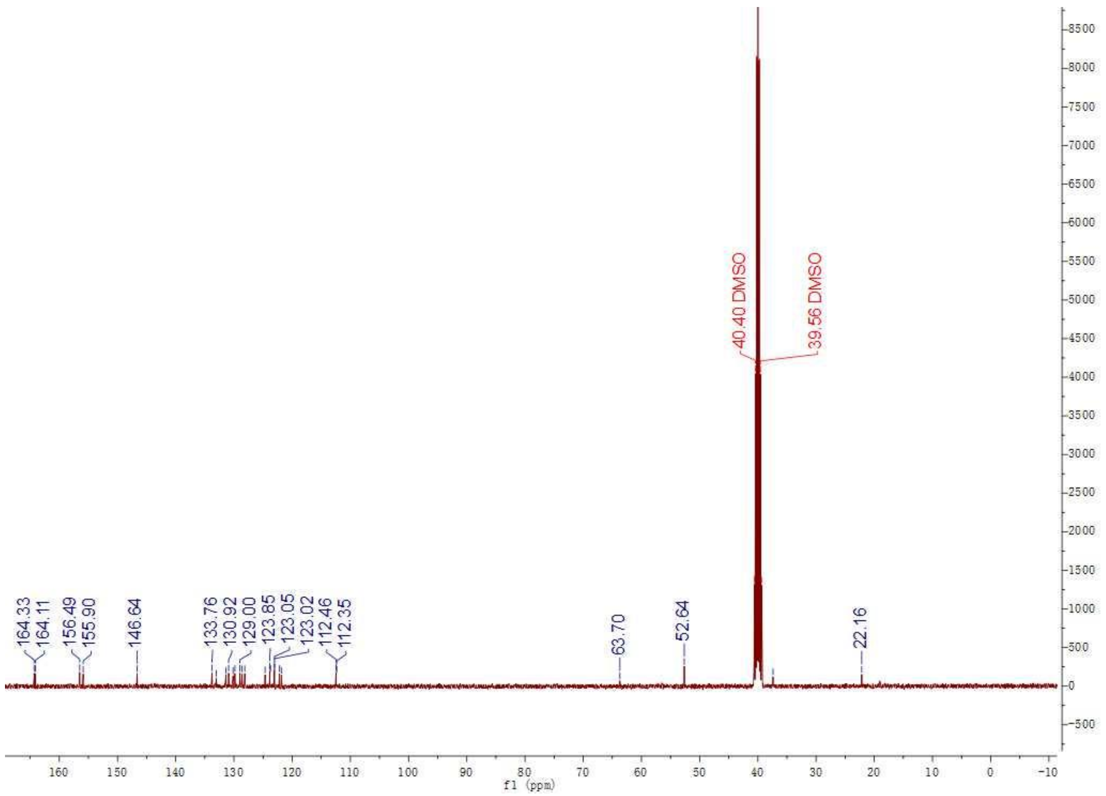

Fig. S108. ^{13}C NMR spectrum of **NDNC** (in DMSO-d_6).

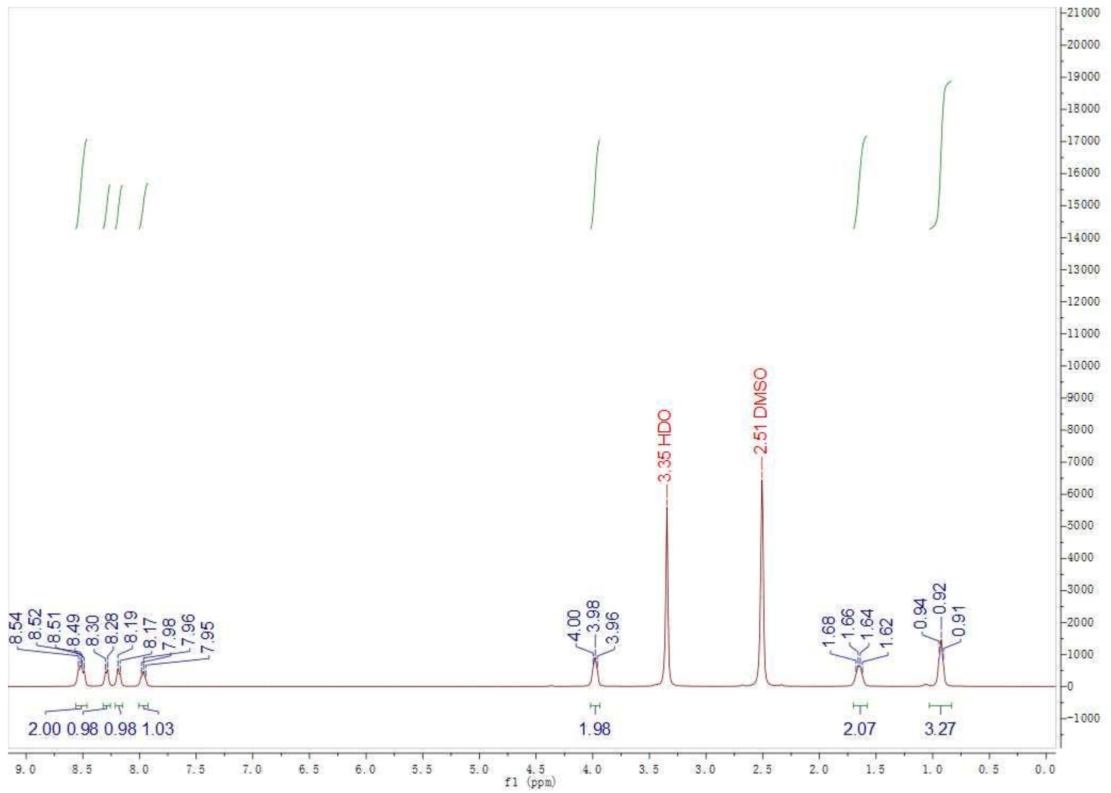

Fig. S109. ^{1}H NMR spectrum of **NBH** (in DMSO-d_6).

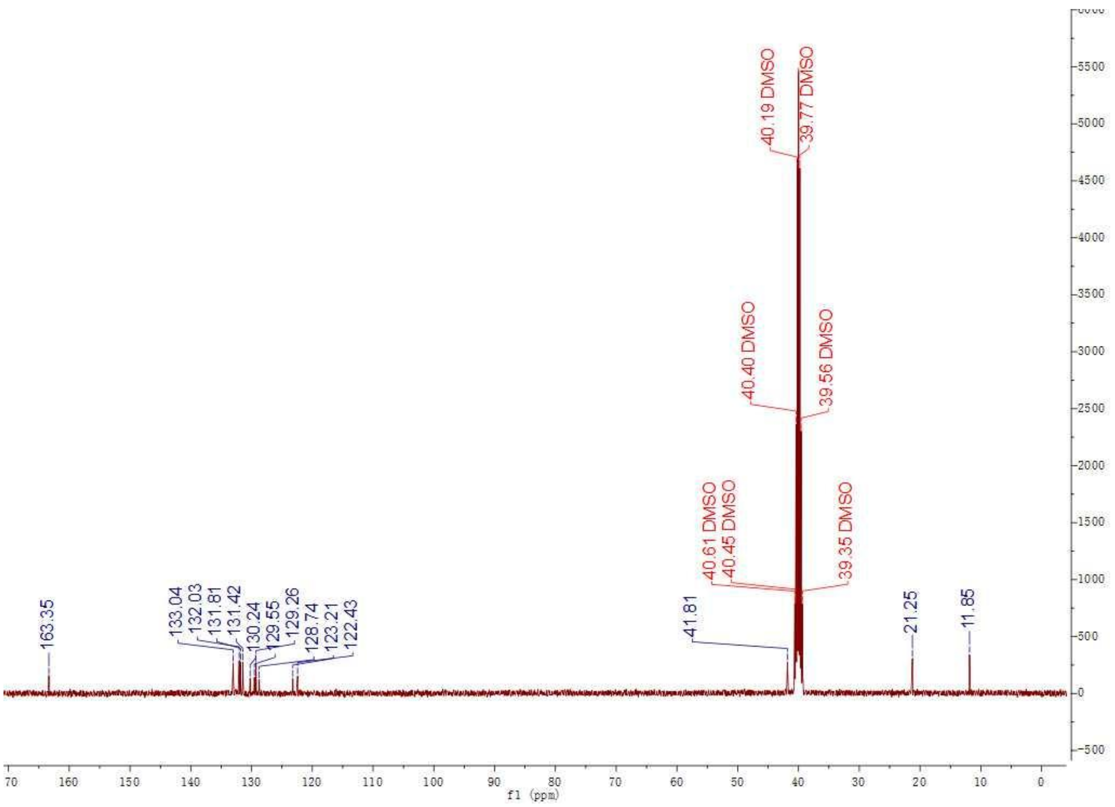

Fig. S110. ^{13}C NMR spectrum of **NBH** (in DMSO-d_6).

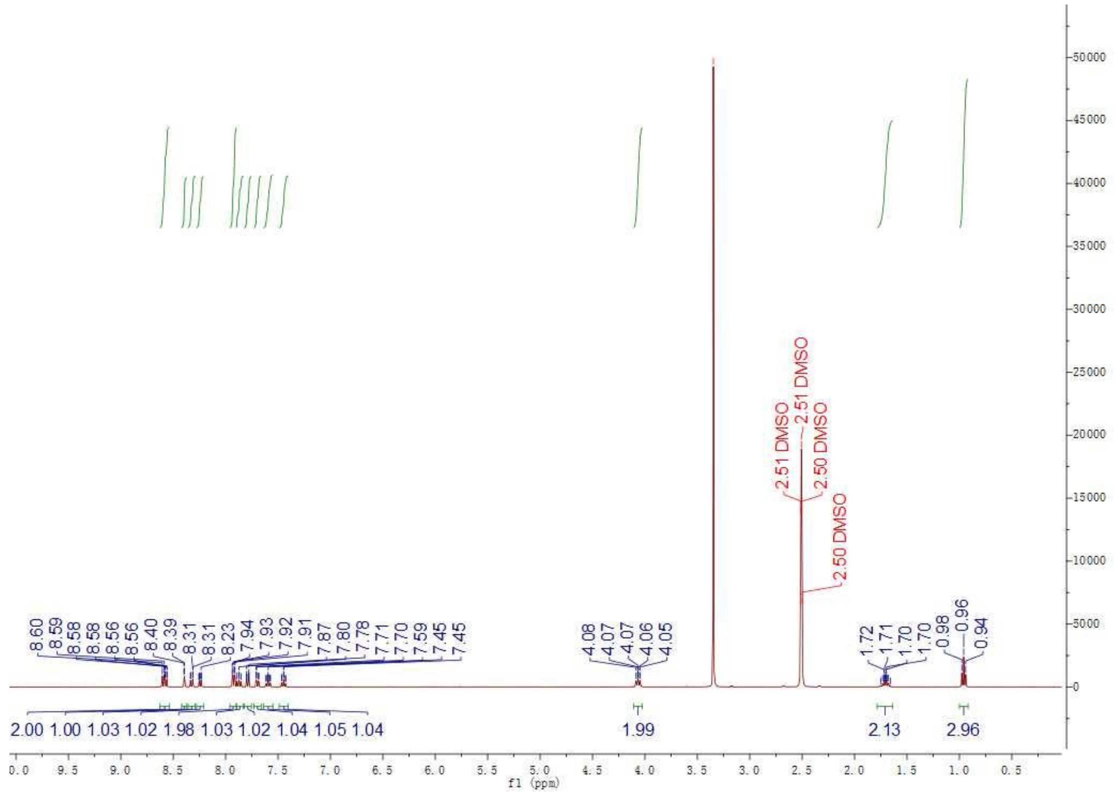

Fig. S111. ^{1}H NMR spectrum of **NDH** (in DMSO-d_6).

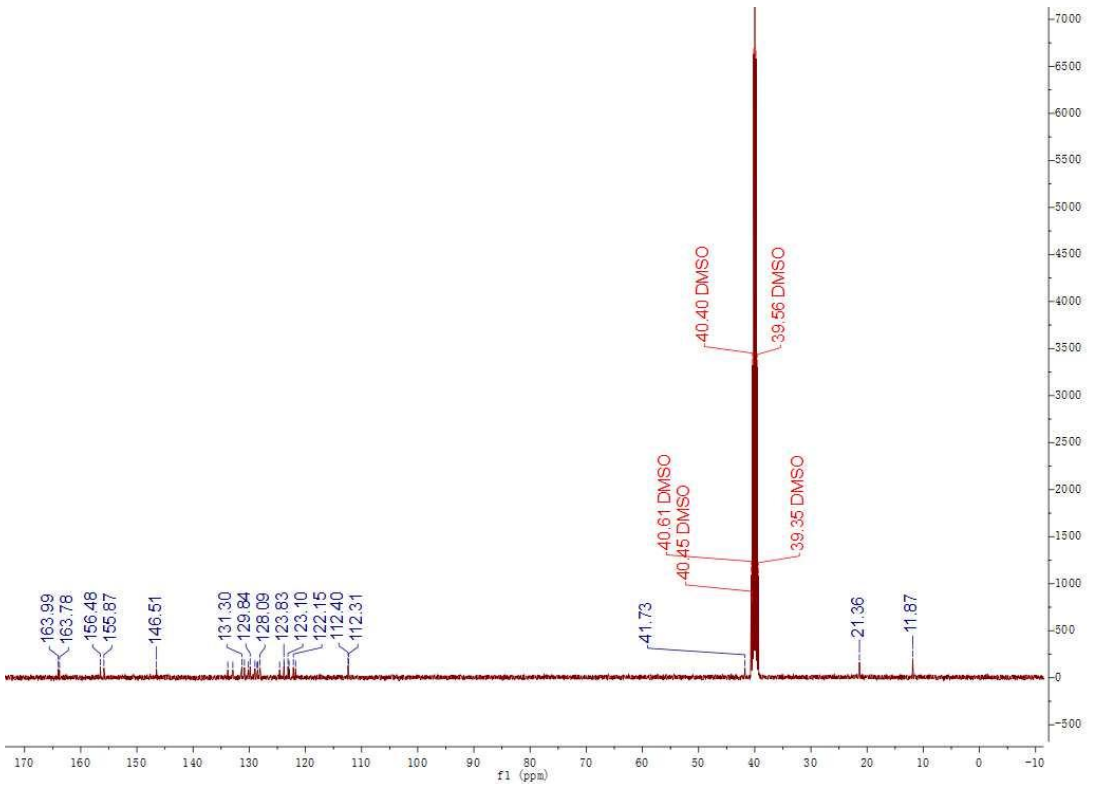

Fig. S112. ^{13}C NMR spectrum of **NDH** (in DMSO-d_6).

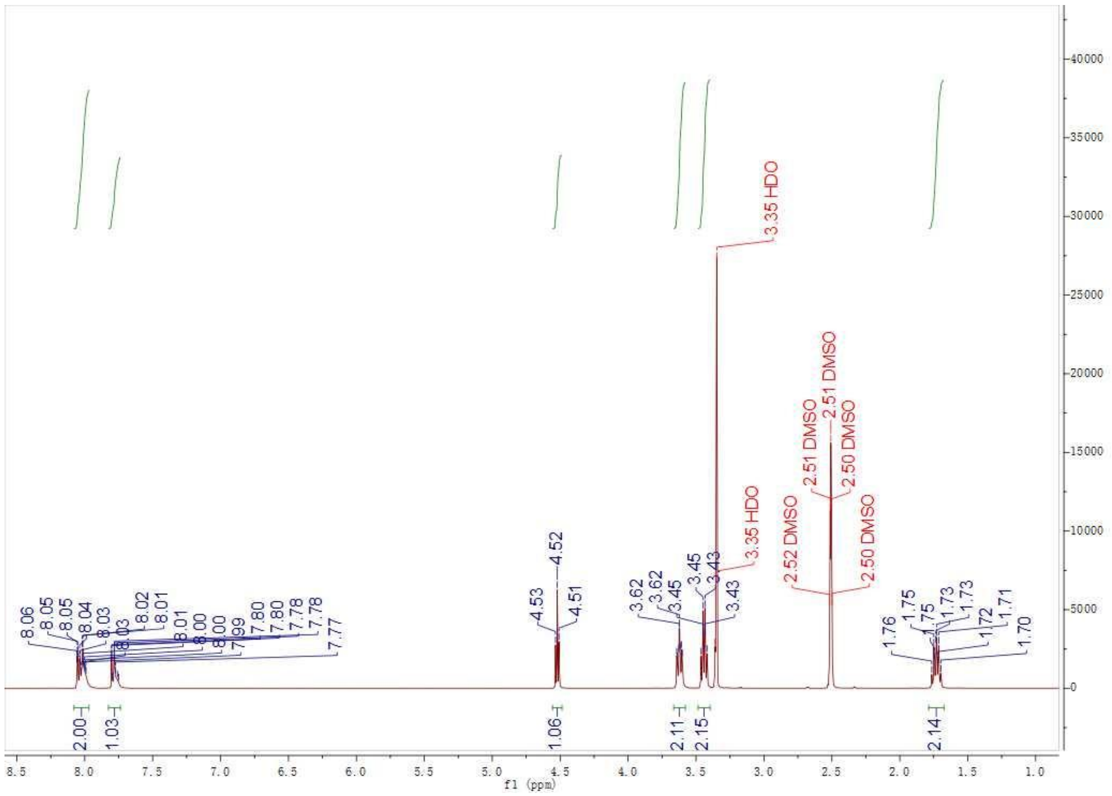

Fig. S113. ^{1}H NMR spectrum of **PBOH** (in DMSO-d_6).

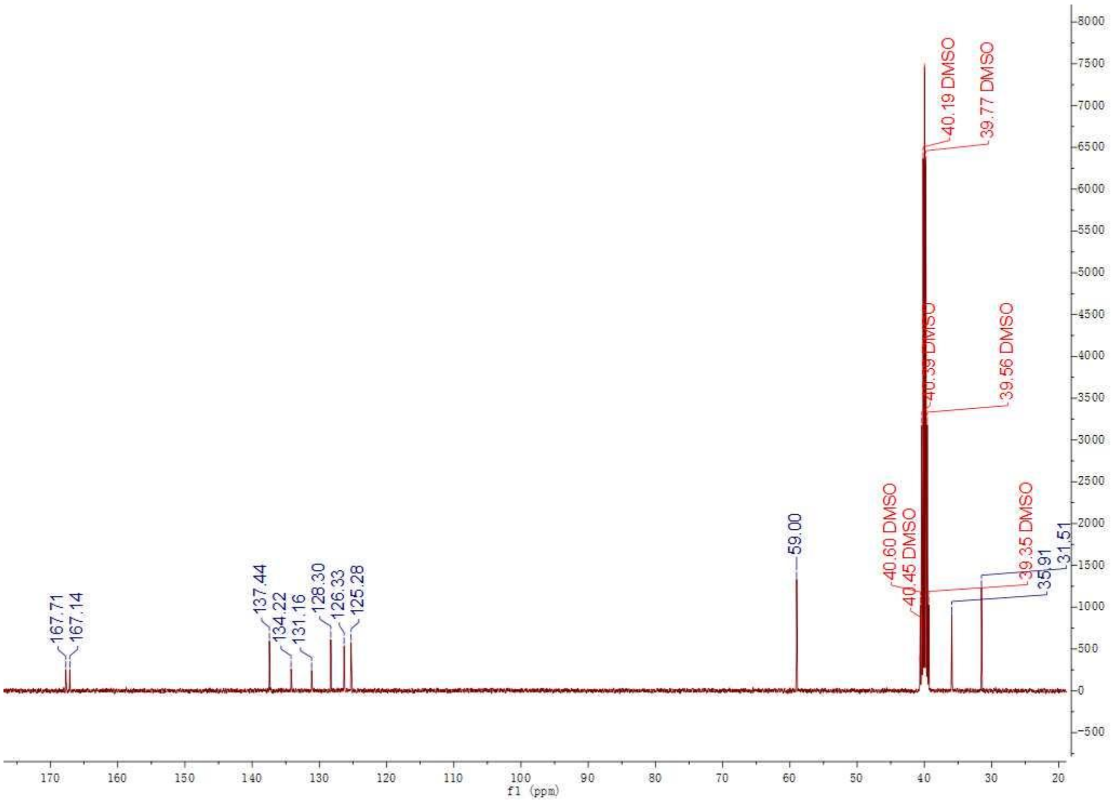

Fig. S114. ^{13}C NMR spectrum of **PBOH** (in DMSO-d_6).

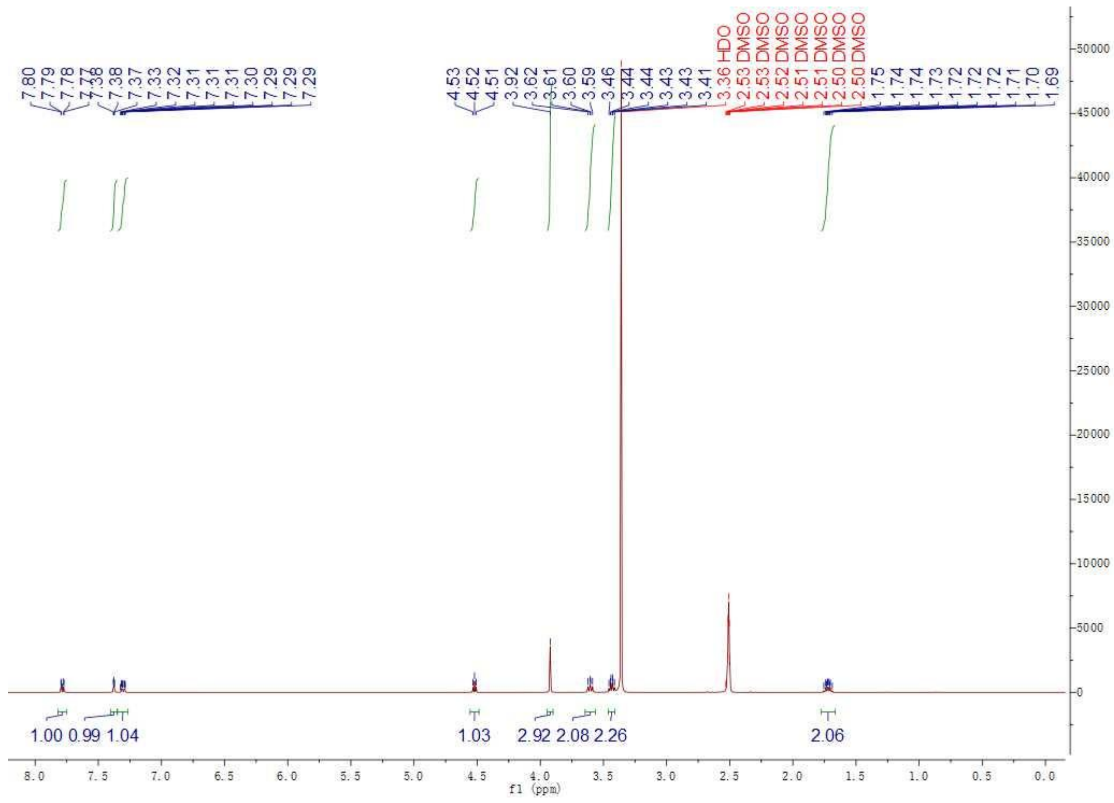

Fig. S115. ^{1}H NMR spectrum of **POOH** (in DMSO-d_6).

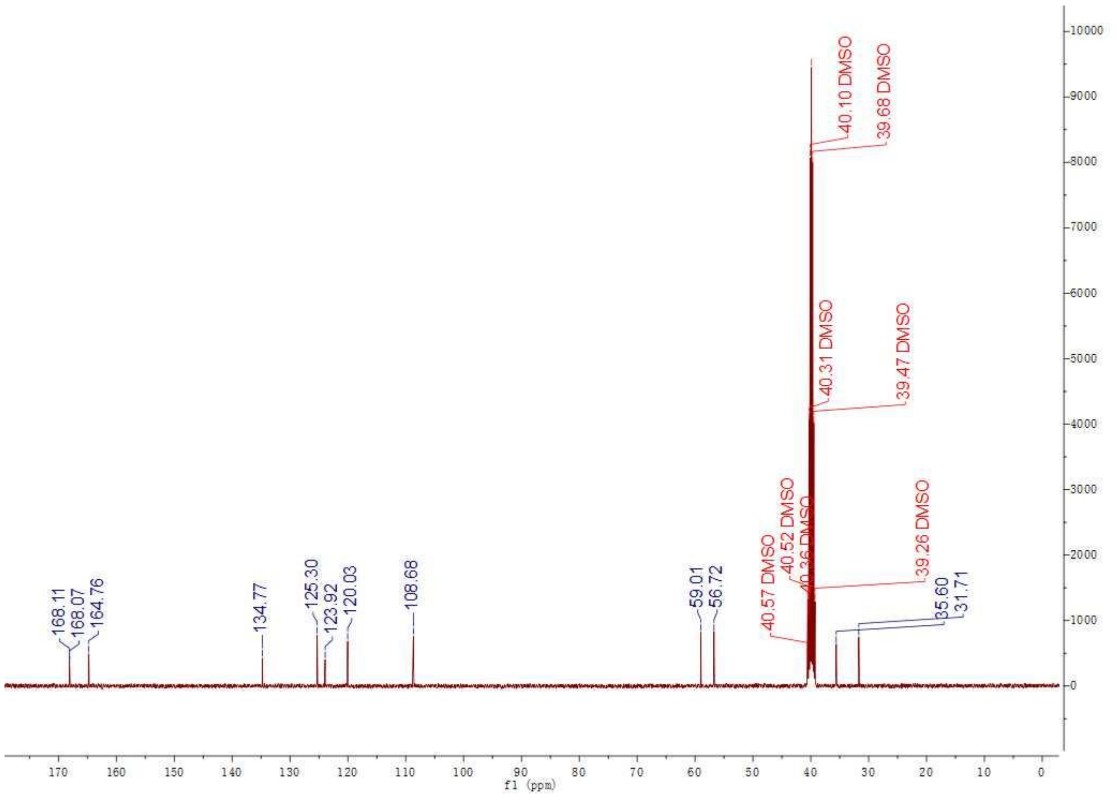

Fig. S116. ^{13}C NMR spectrum of **POOH** (in DMSO-d_6).

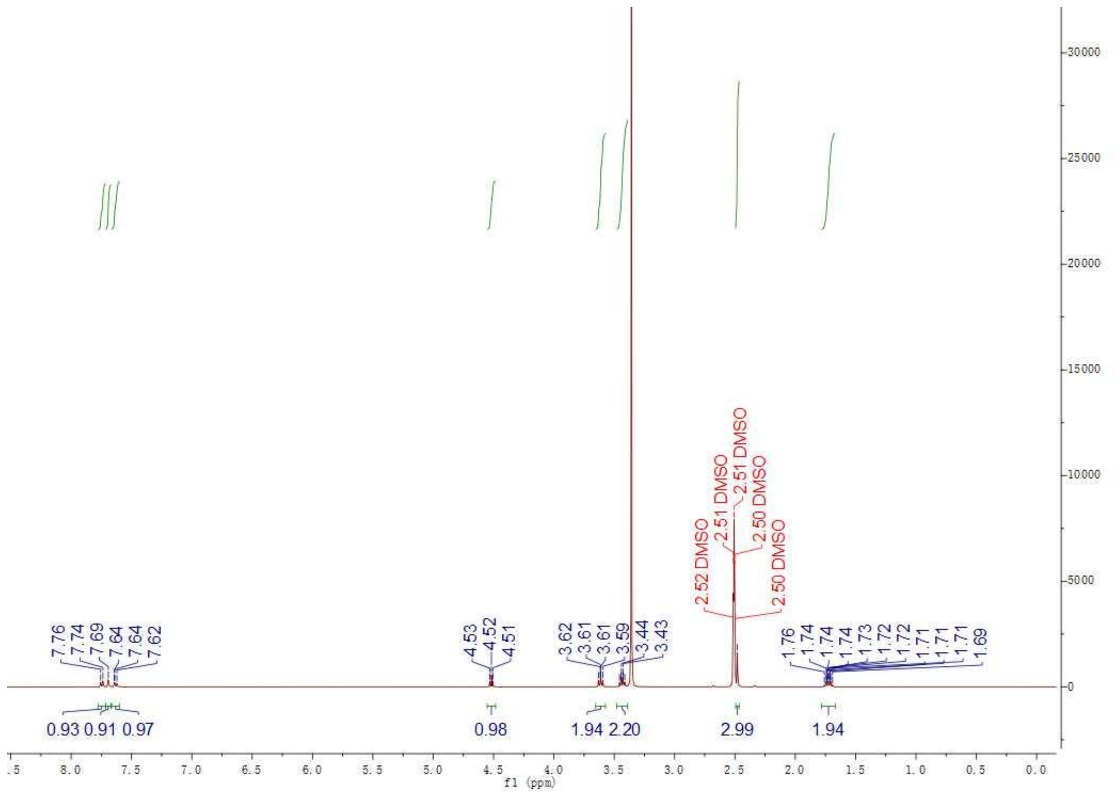

Fig. S117. ^{1}H NMR spectrum of **PMOH** (in DMSO-d_6).

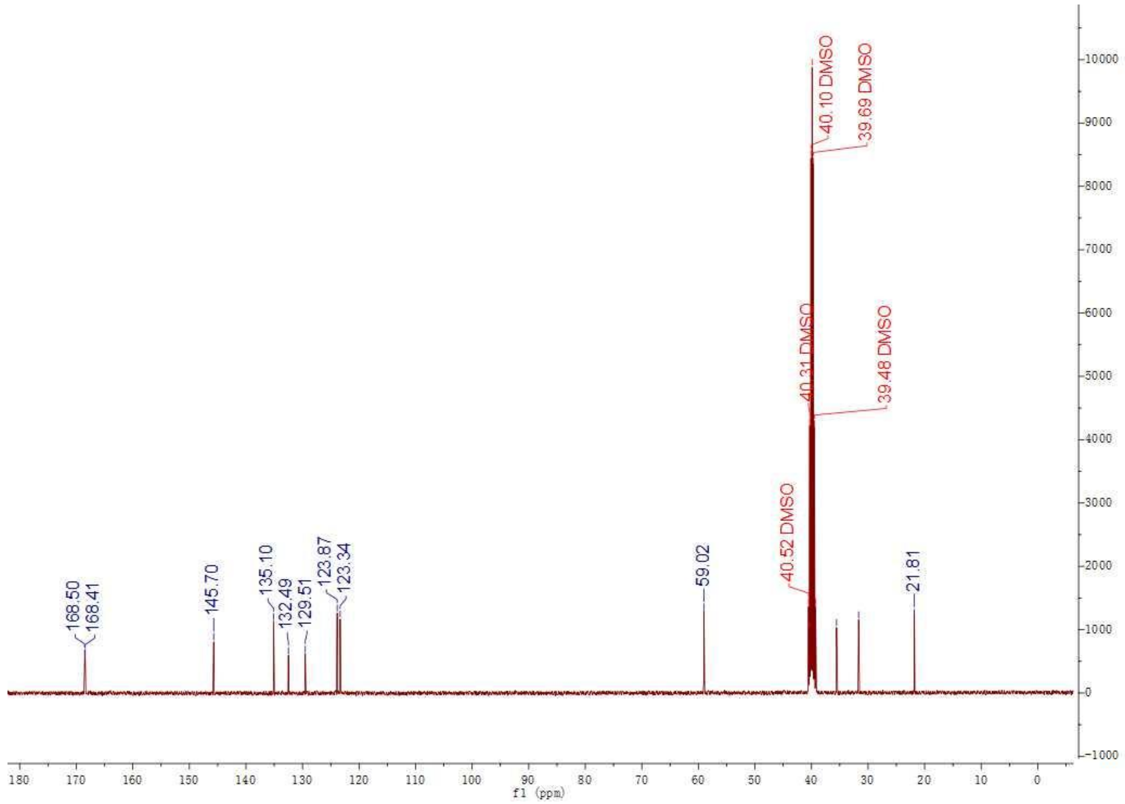

Fig. S118. ^{13}C NMR spectrum of **PMOH** (in DMSO-d_6).

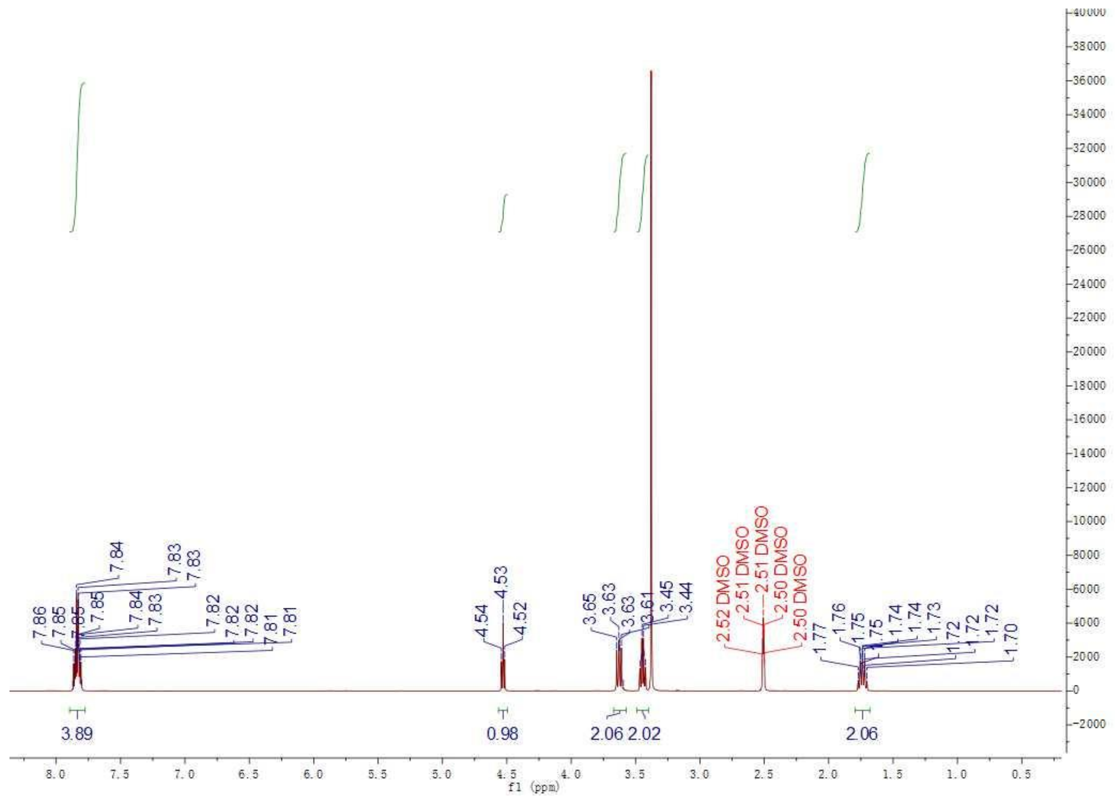

Fig. S119. ^{1}H NMR spectrum of **PHOH** (in DMSO-d_6).

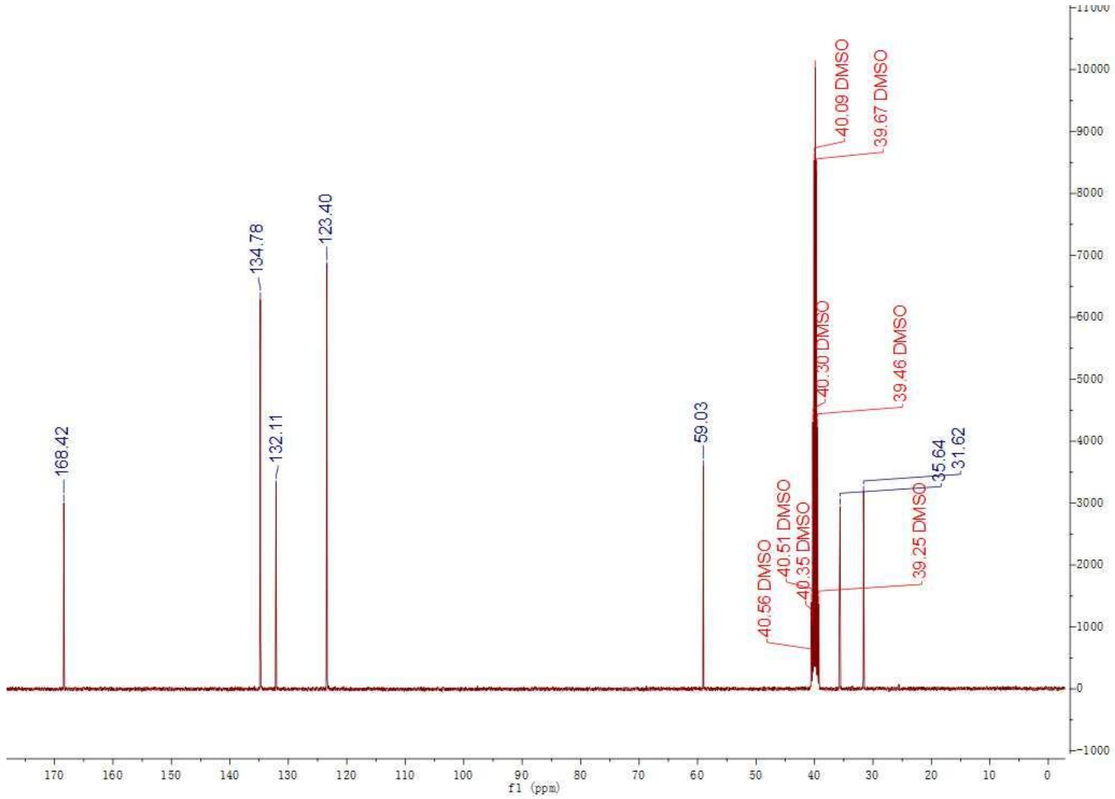

Fig. S120. ^{13}C NMR spectrum of **PHOH** (in DMSO-d_6).

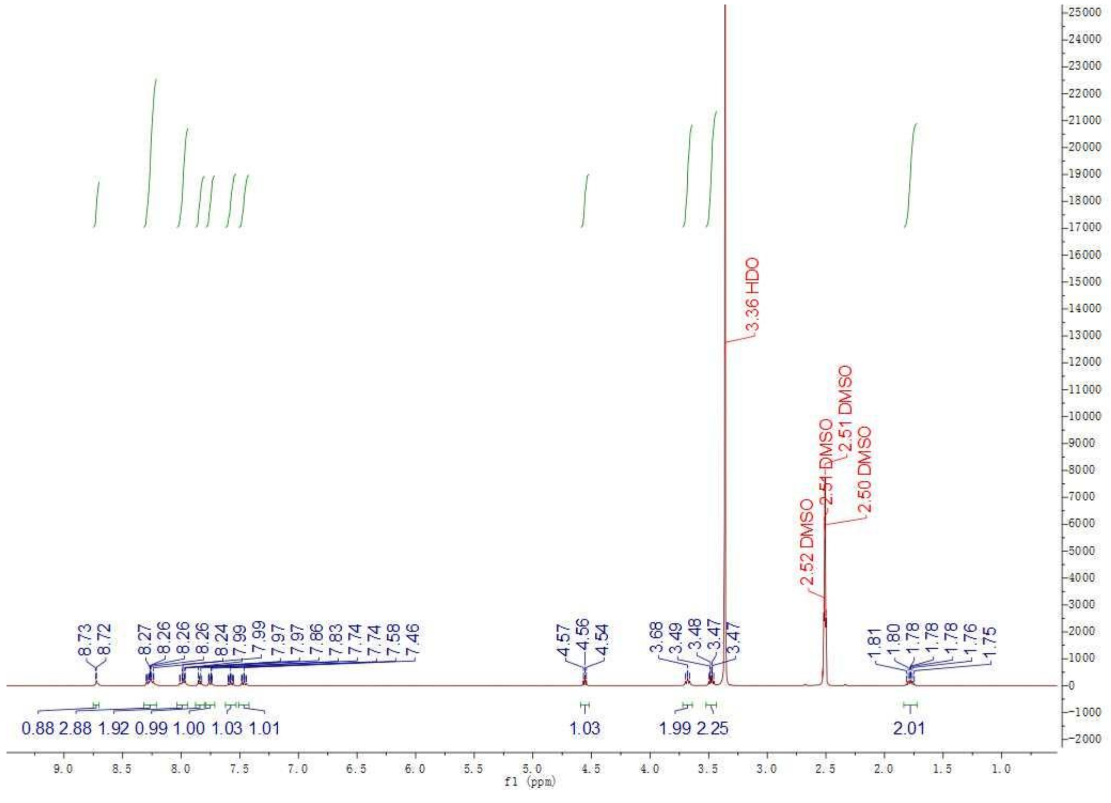

Fig. S121. ^{1}H NMR spectrum of **PDOH** (in DMSO-d_6).

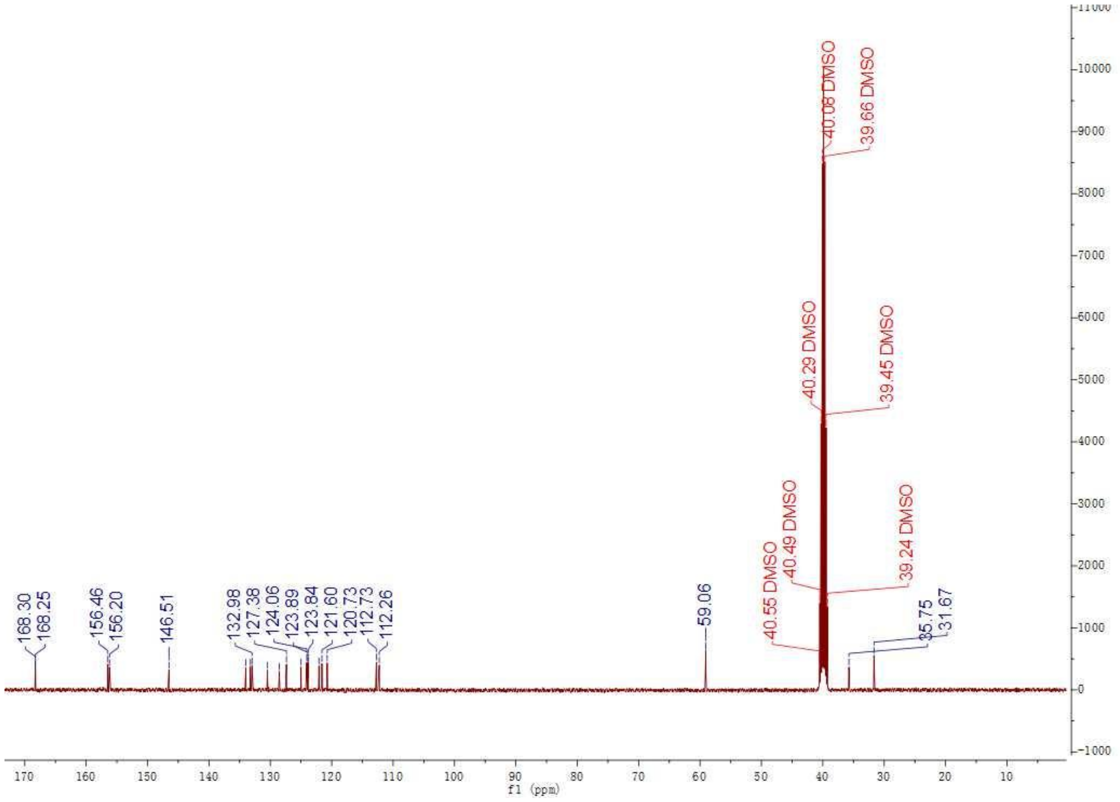

Fig. S122. ^{13}C NMR spectrum of **PDOH** (in DMSO-d_6).

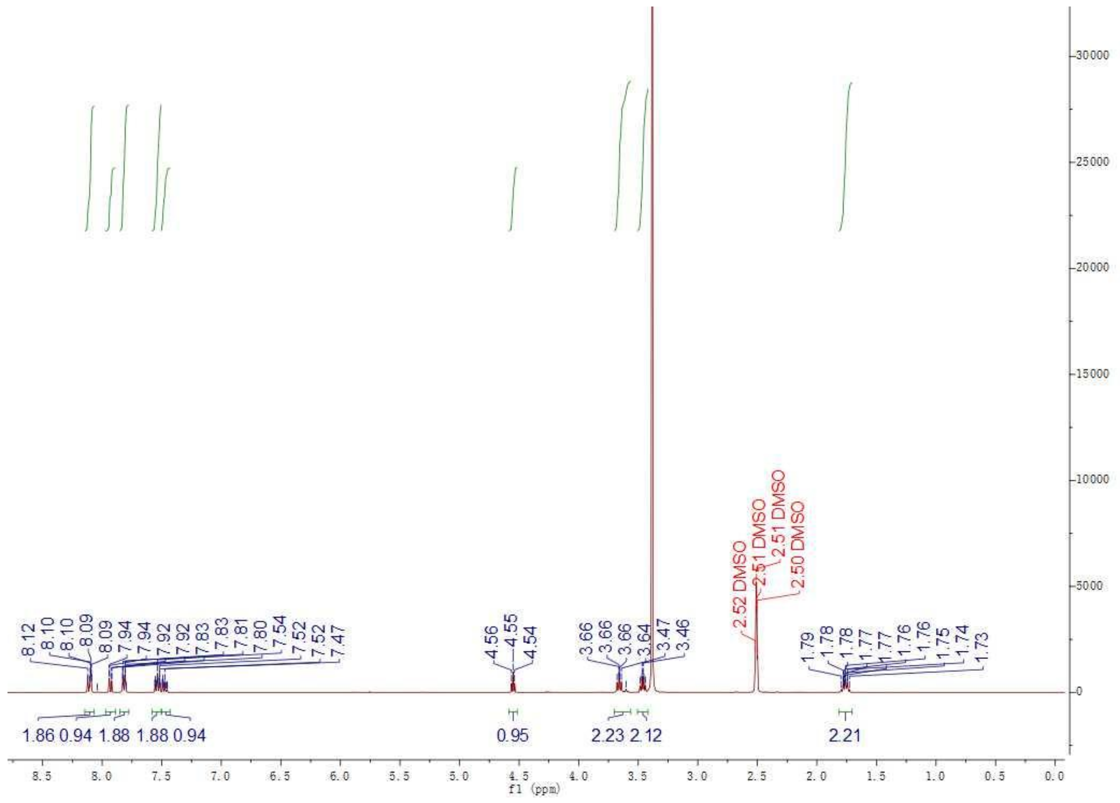

Fig. S123. ^{1}H NMR spectrum of **PPOH** (in DMSO-d_6).

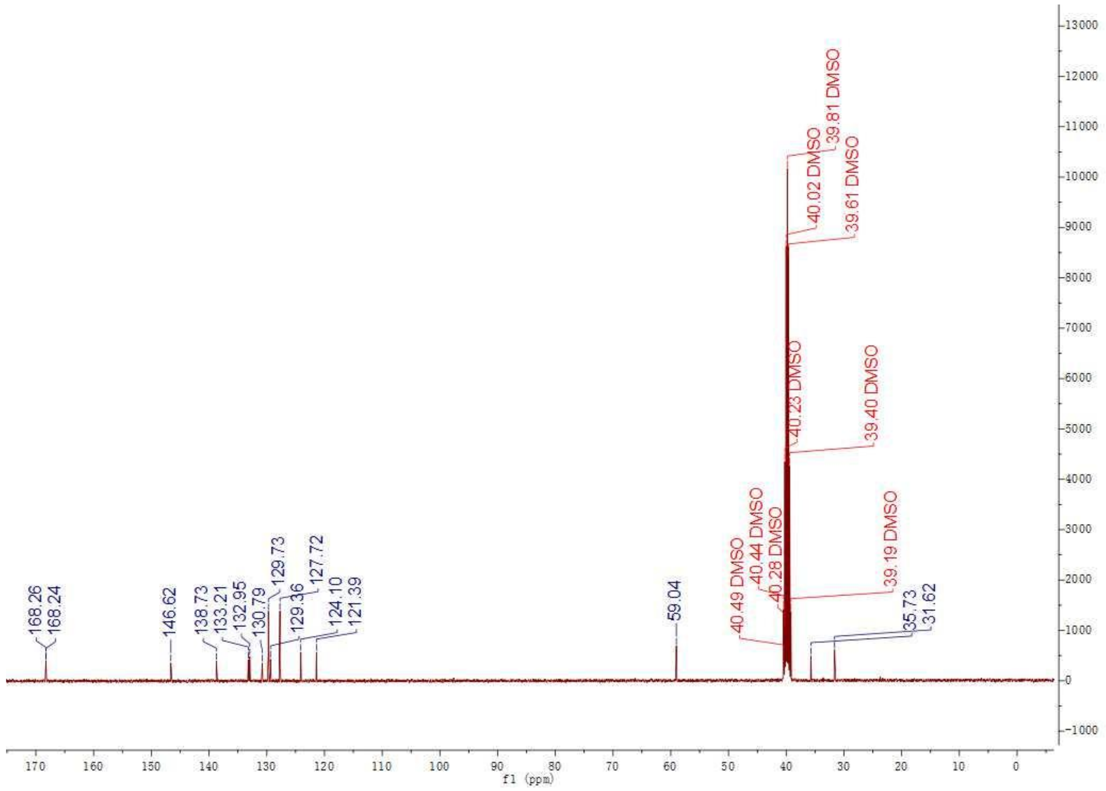

Fig. S124. ^{13}C NMR spectrum of **PPOH** (in DMSO-d_6).

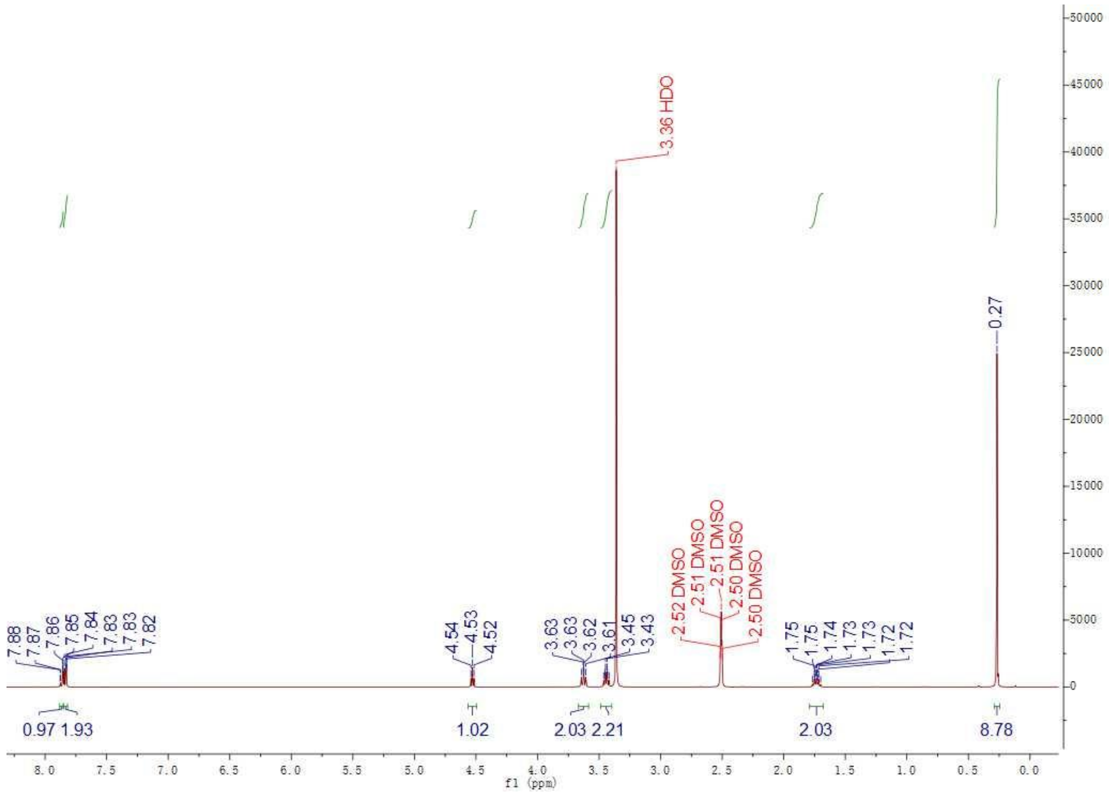

Fig. S125. ^{1}H NMR spectrum of **PEOH** (in DMSO-d_6).

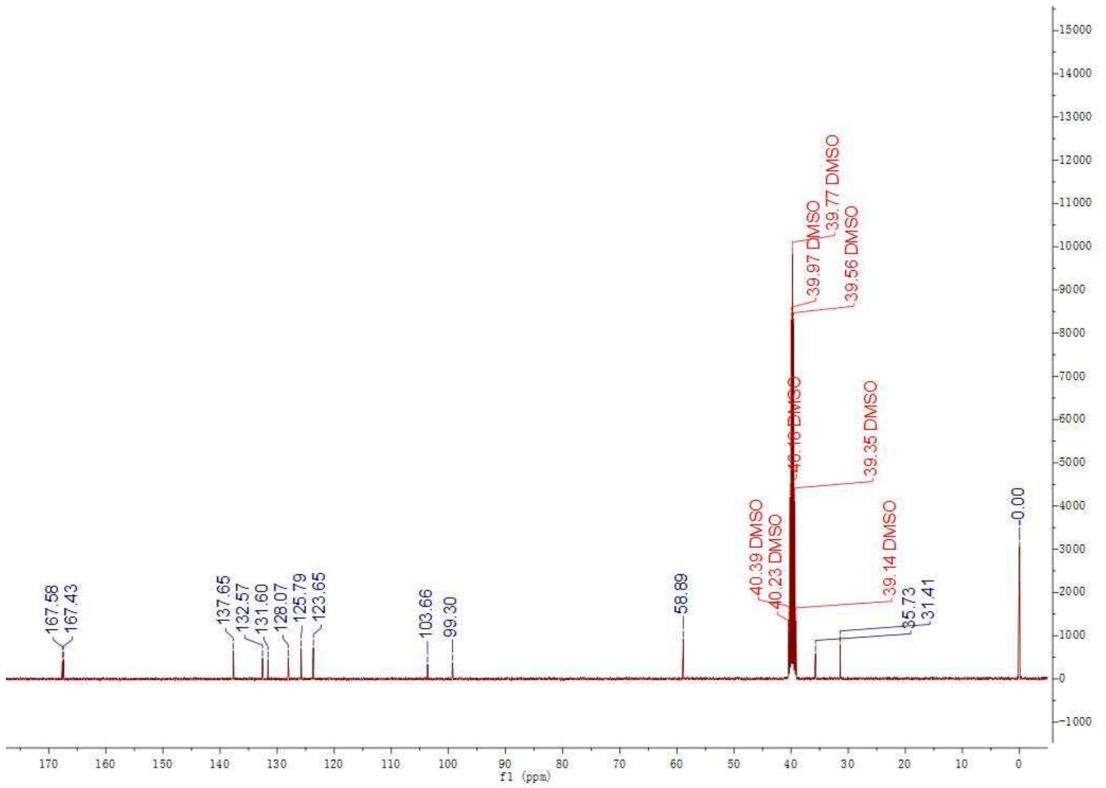

Fig. S126. ^{13}C NMR spectrum of **PEOH** (in DMSO-d_6).

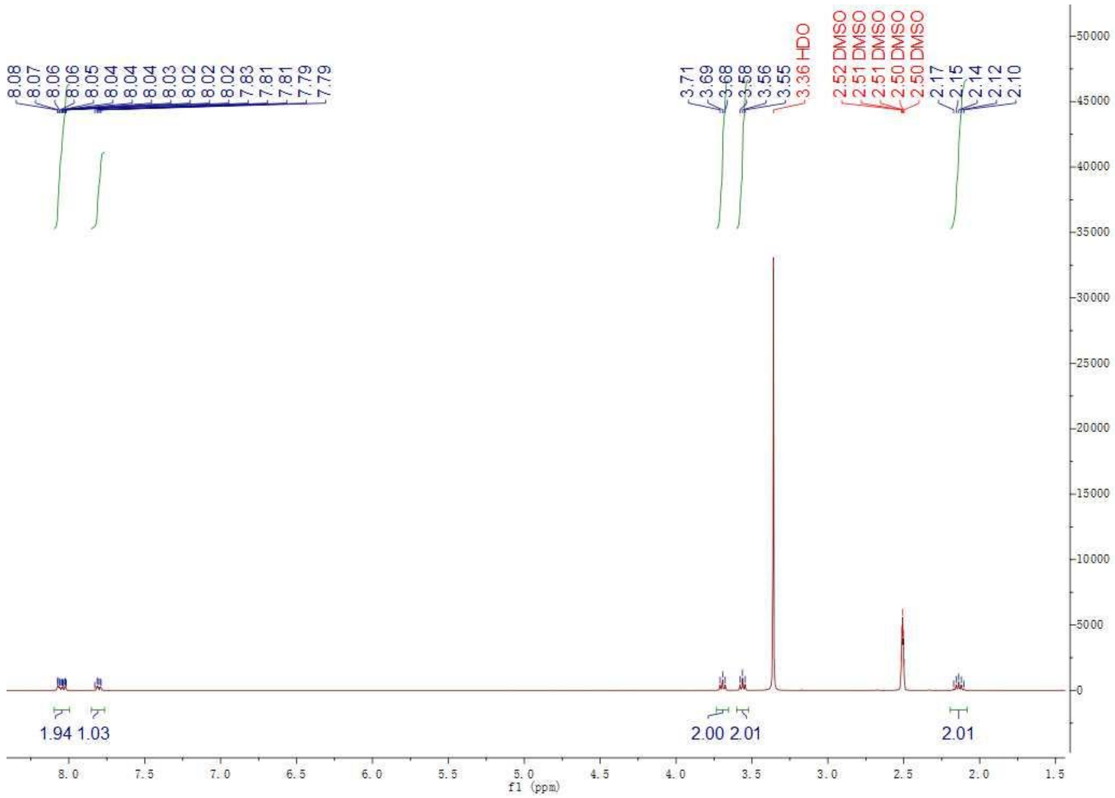

Fig. S127. ^{1}H NMR spectrum of **PBB** (in DMSO-d_6).

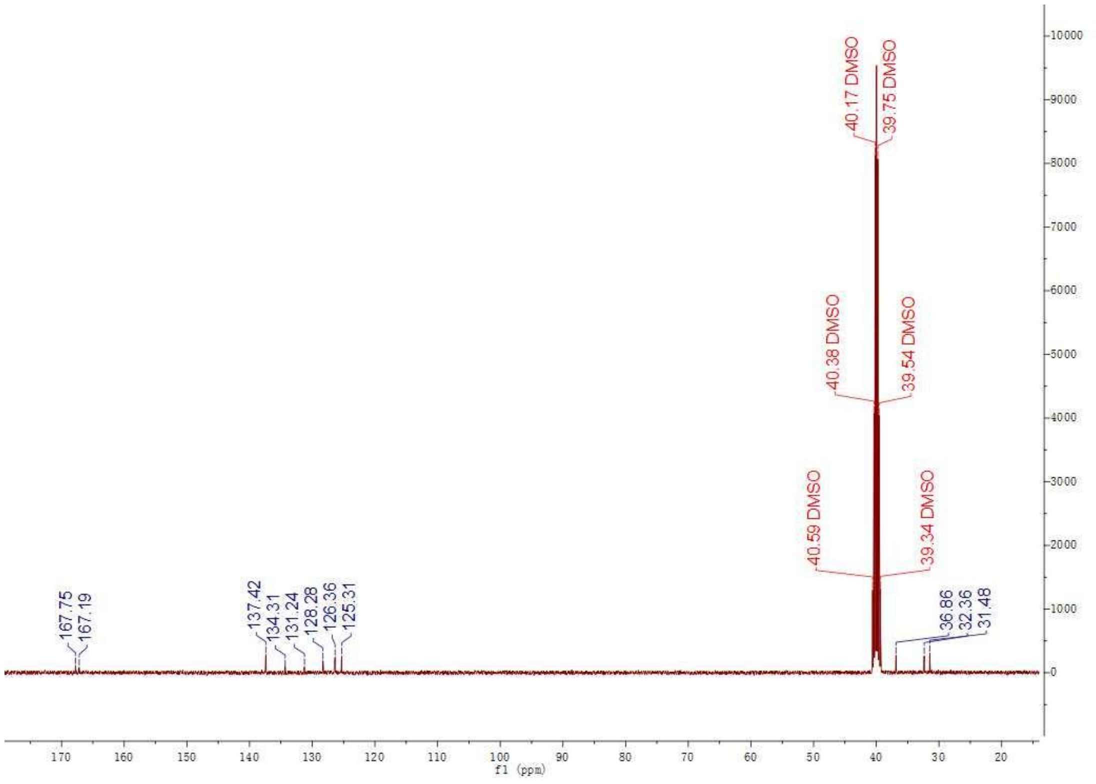

Fig. S128. ^{13}C NMR spectrum of **PBB** (in DMSO-d_6).

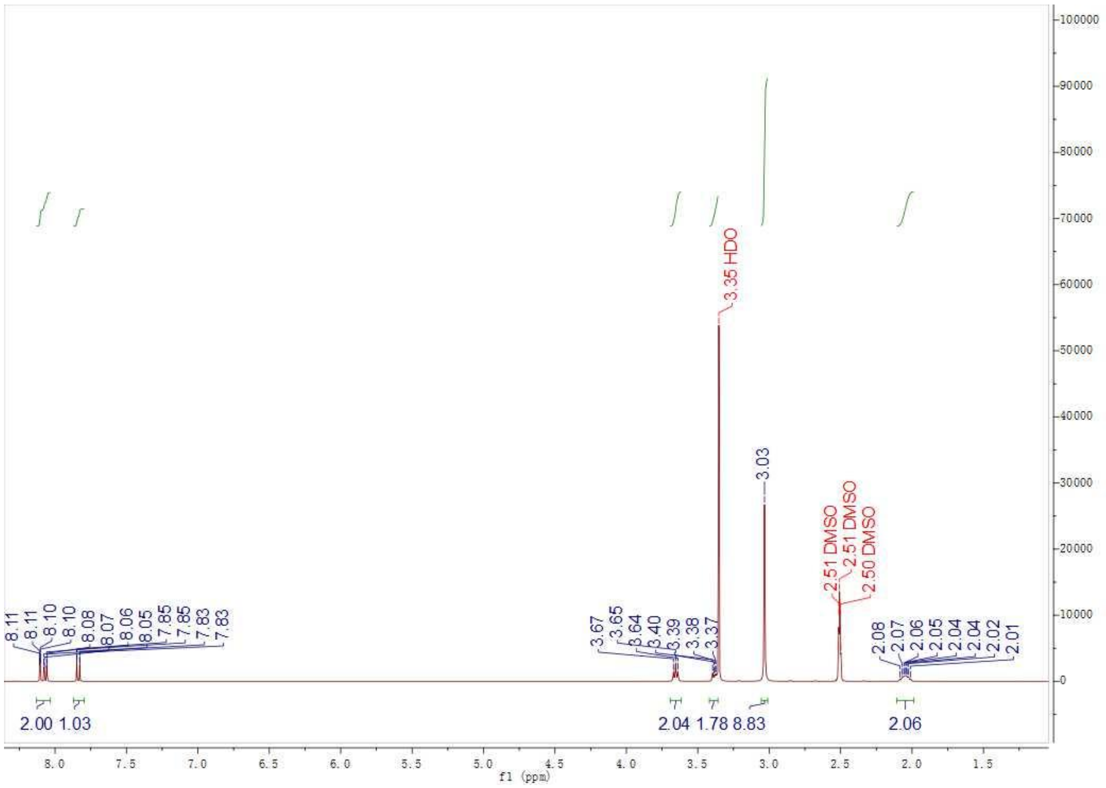

Fig. S129. ^{1}H NMR spectrum of **PBNC** (in DMSO-d_6).

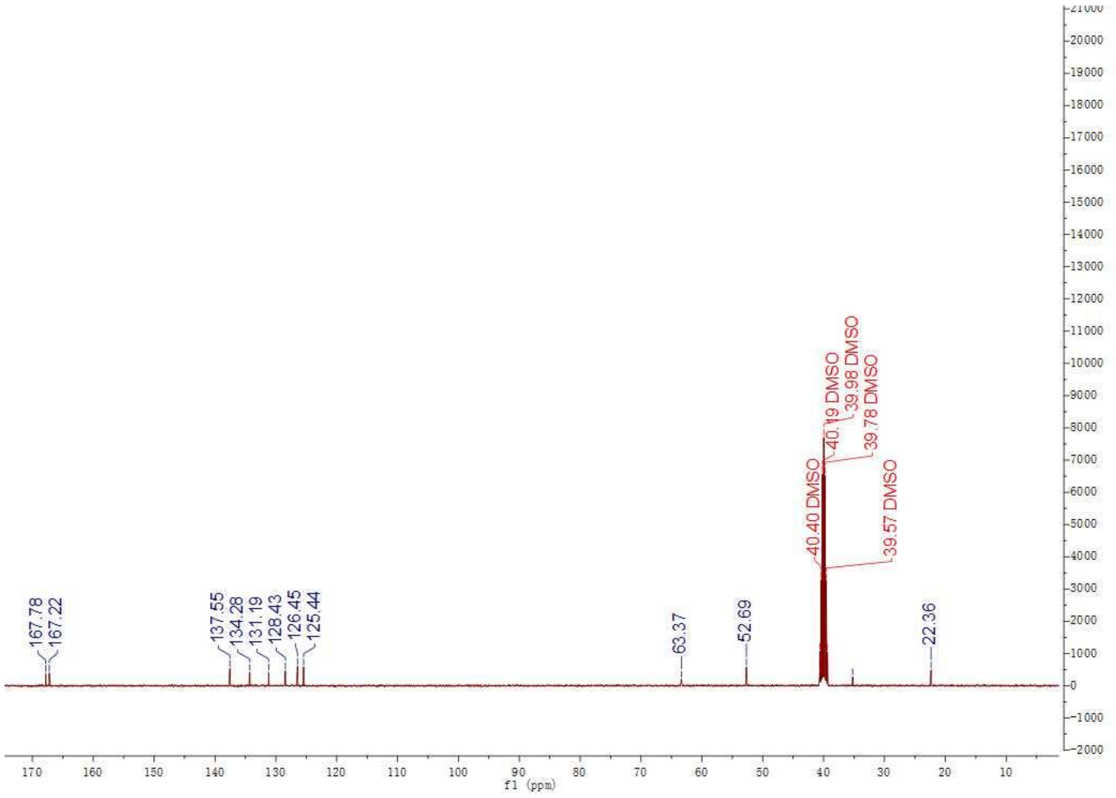

Fig. S130. ^{13}C NMR spectrum of **PBNC** (in DMSO-d_6).

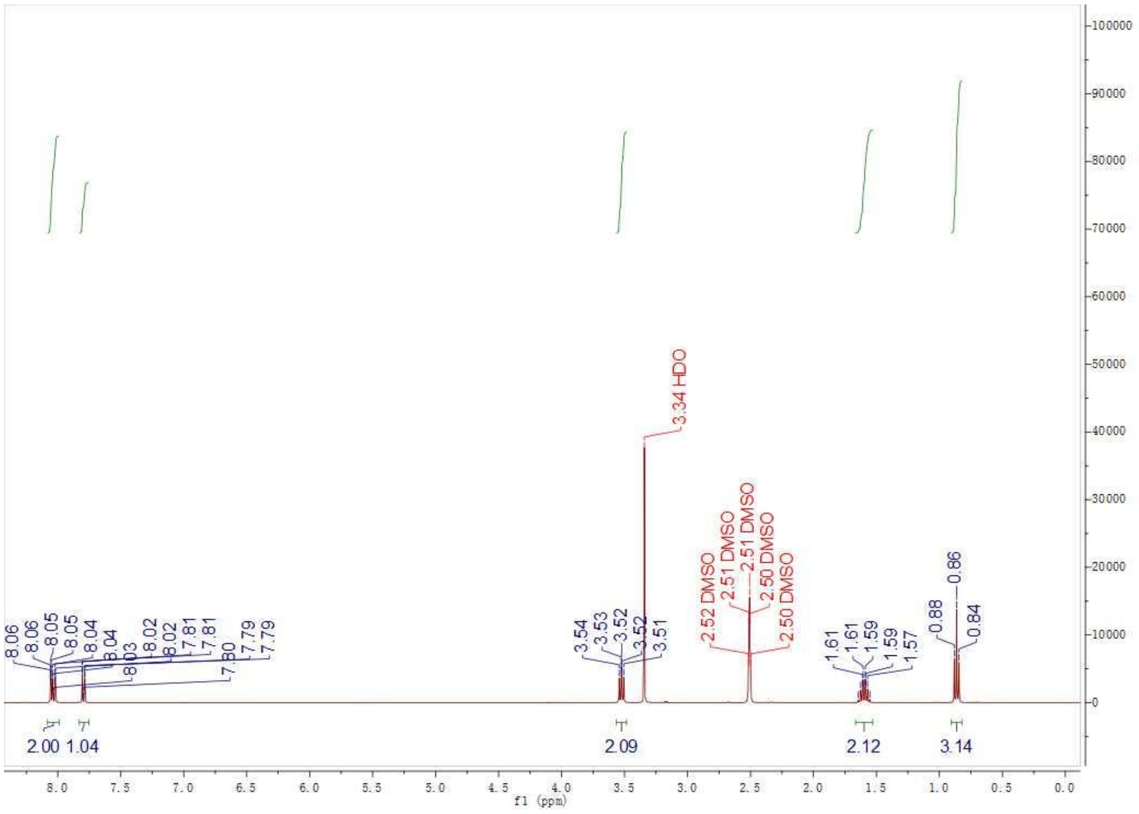

Fig. S131. ^{1}H NMR spectrum of **PBH** (in DMSO-d_6).

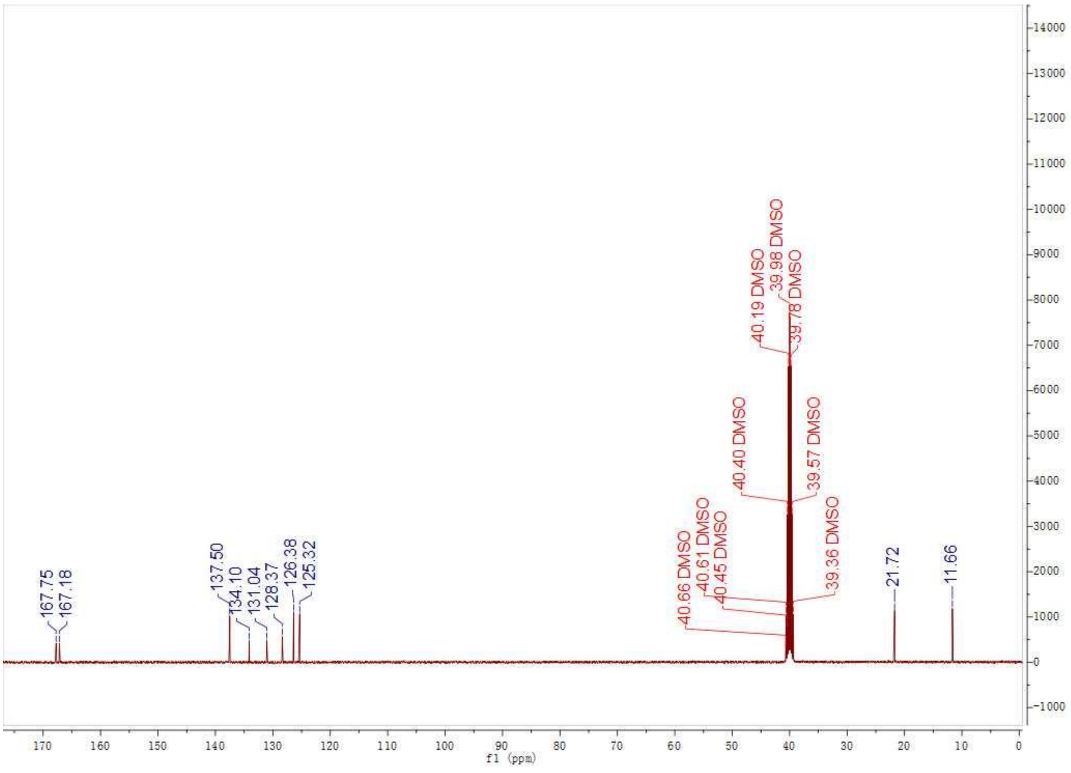

Fig. S132. ^{13}C NMR spectrum of **PBH** (in DMSO-d_6).

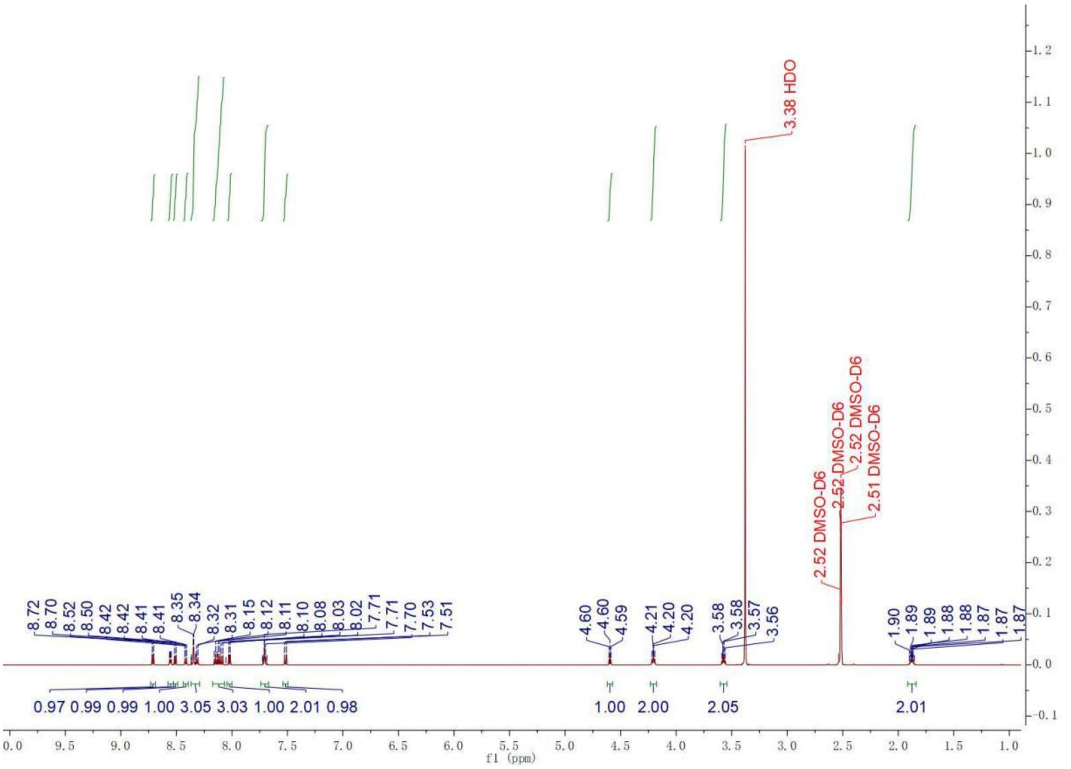

Fig. S133. ^{1}H NMR spectrum of **NPOH** (in DMSO-d_6).

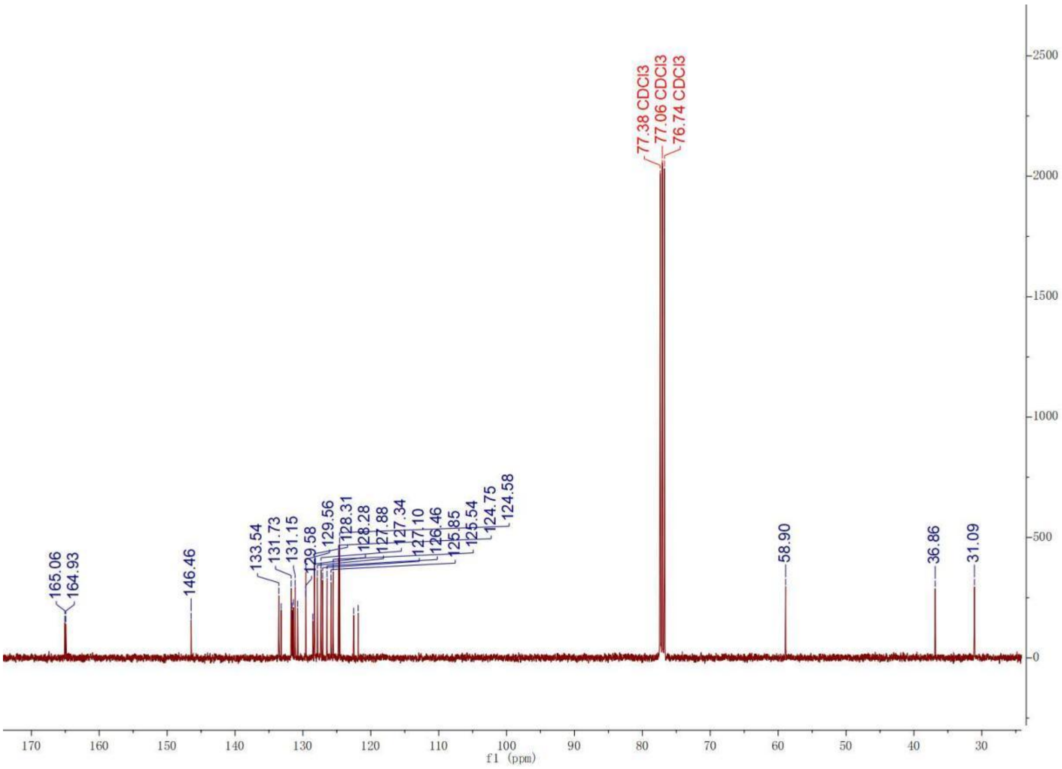

Fig. S134. ^{13}C NMR spectrum of **NPOH** (in $CDCl_3$).

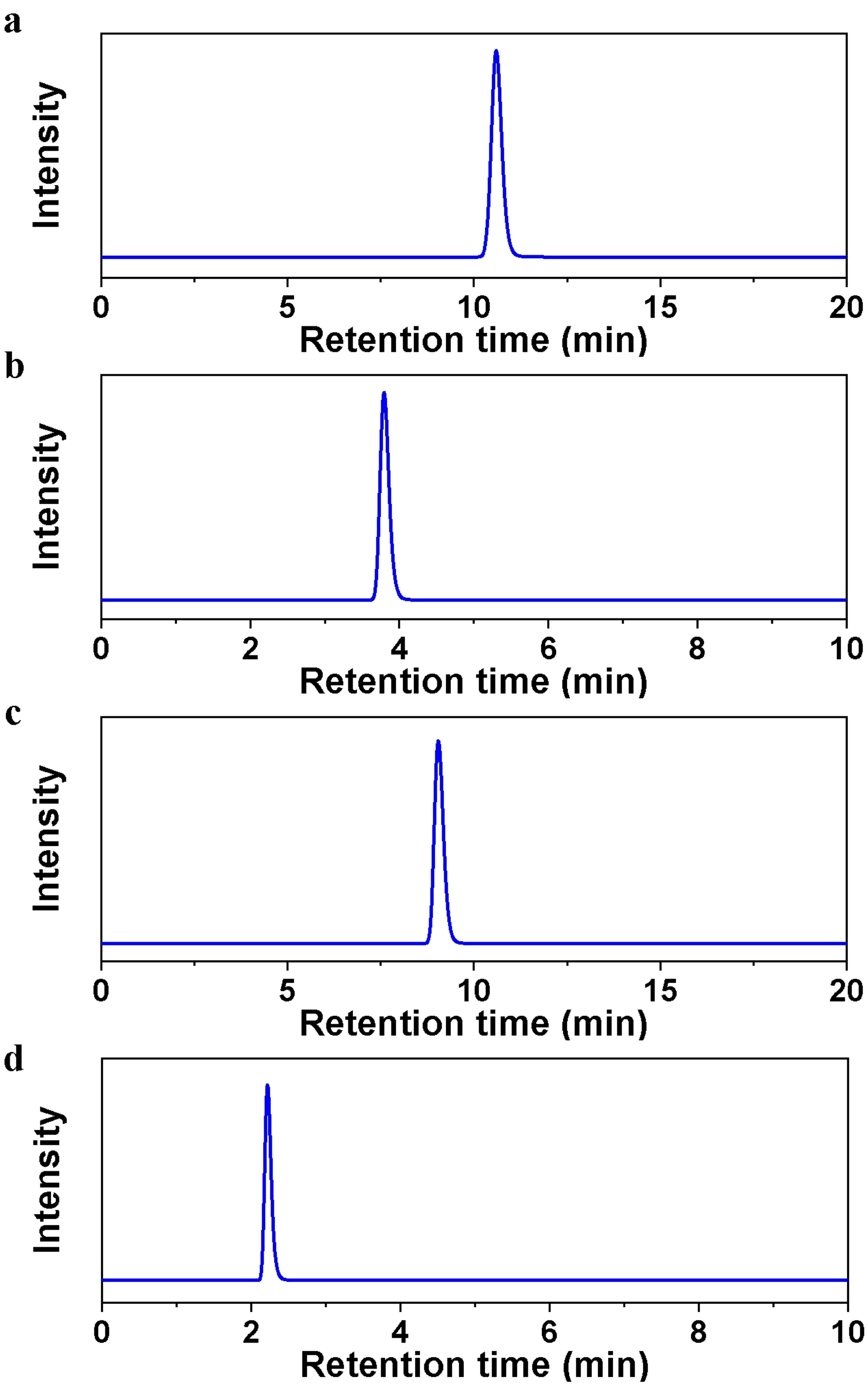

Fig. S135. HPLC curves for a) **PDOH**, b) **PPOH**, c) **PEOH** and d) **POOH** using H_2O/MeCN as mobile phase.

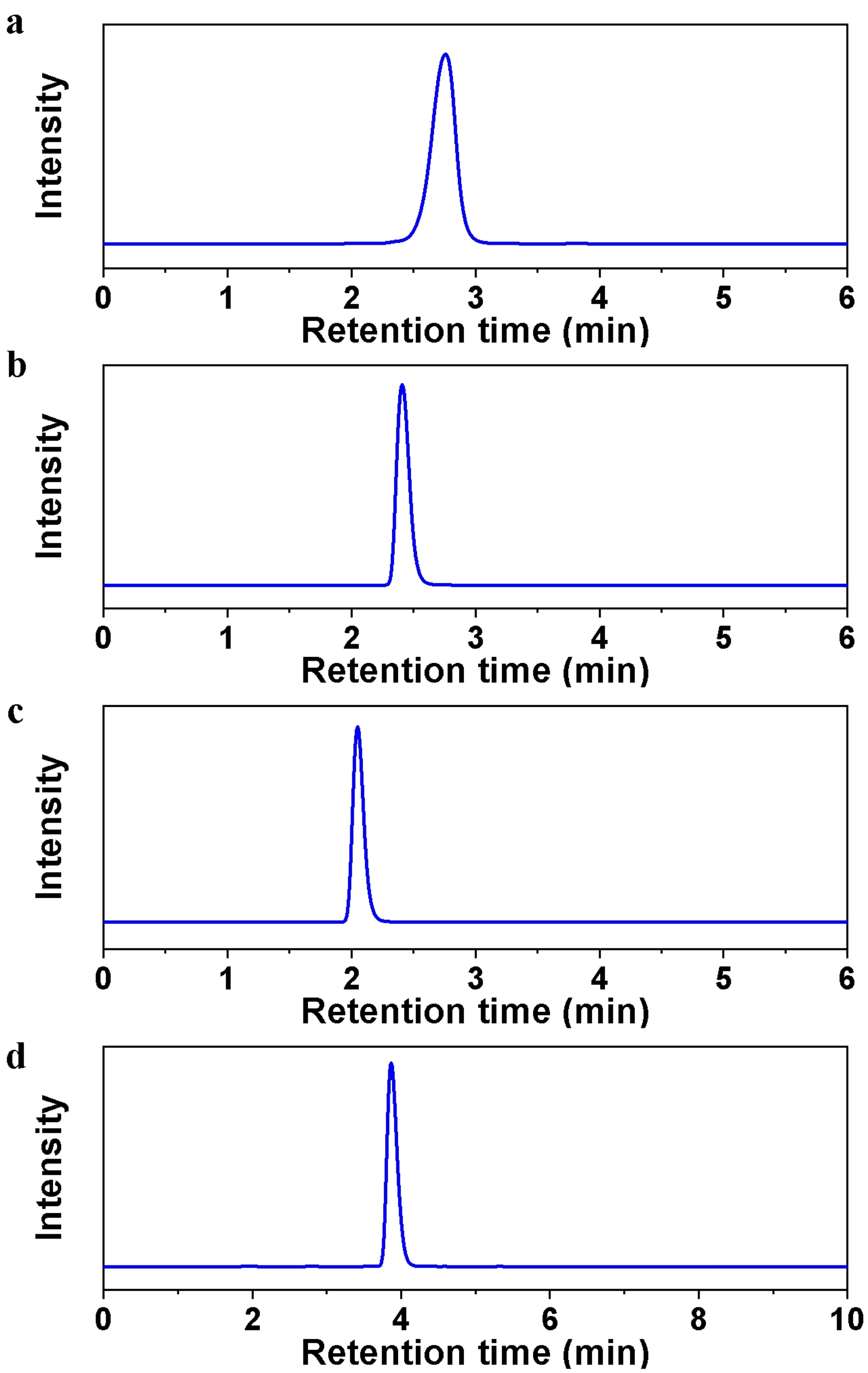

Fig. S136. HPLC curves for a) **PBOH**, b) **PMOH**, c) **PHOH** and d) **PBH** using H_2O/MeCN as mobile phase.

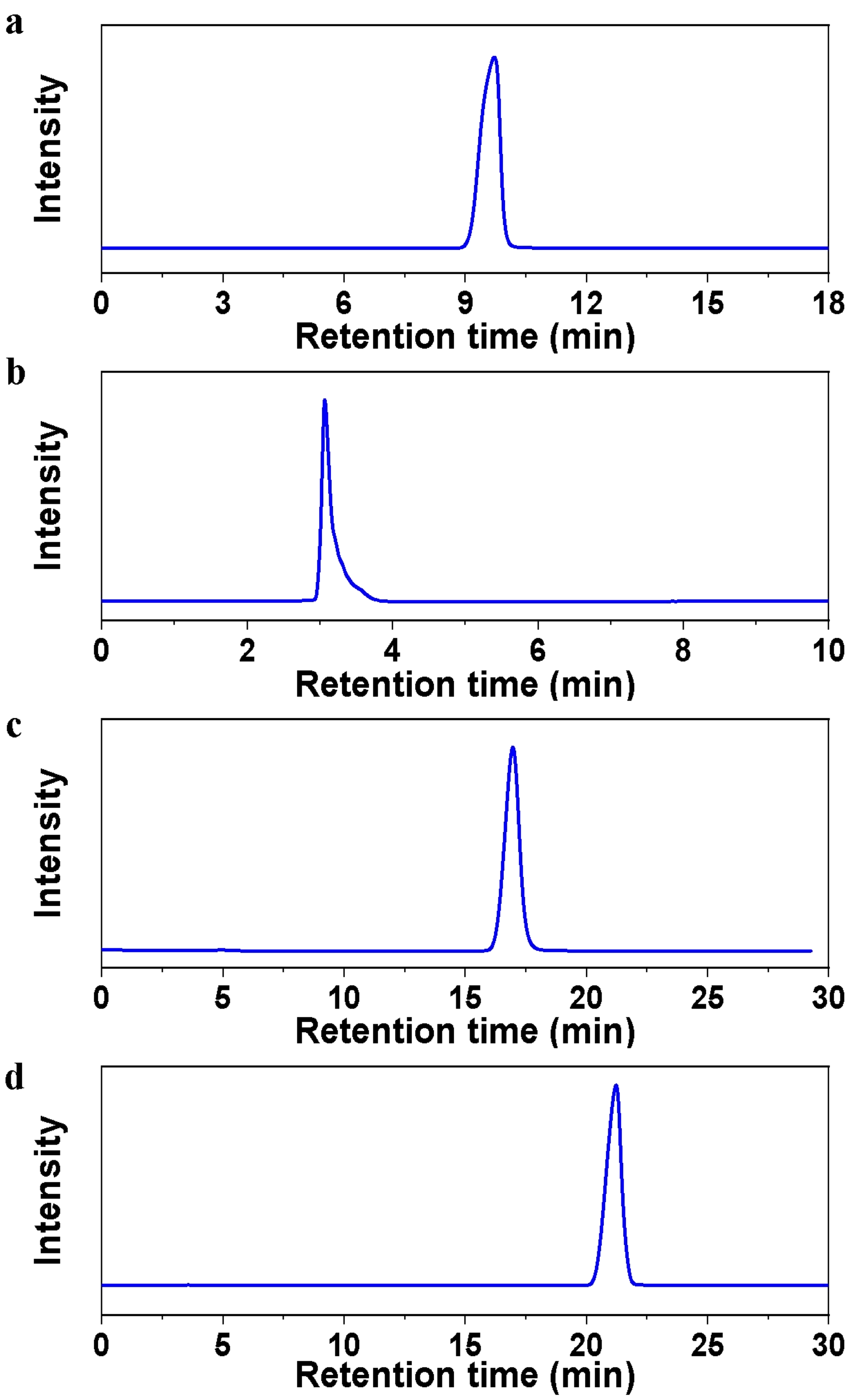

Fig. S137. HPLC curves for a) **PBB**, b) **PBNC**, c) **NDH** and d) **NDOH** using H_2O/MeCN as mobile phase.

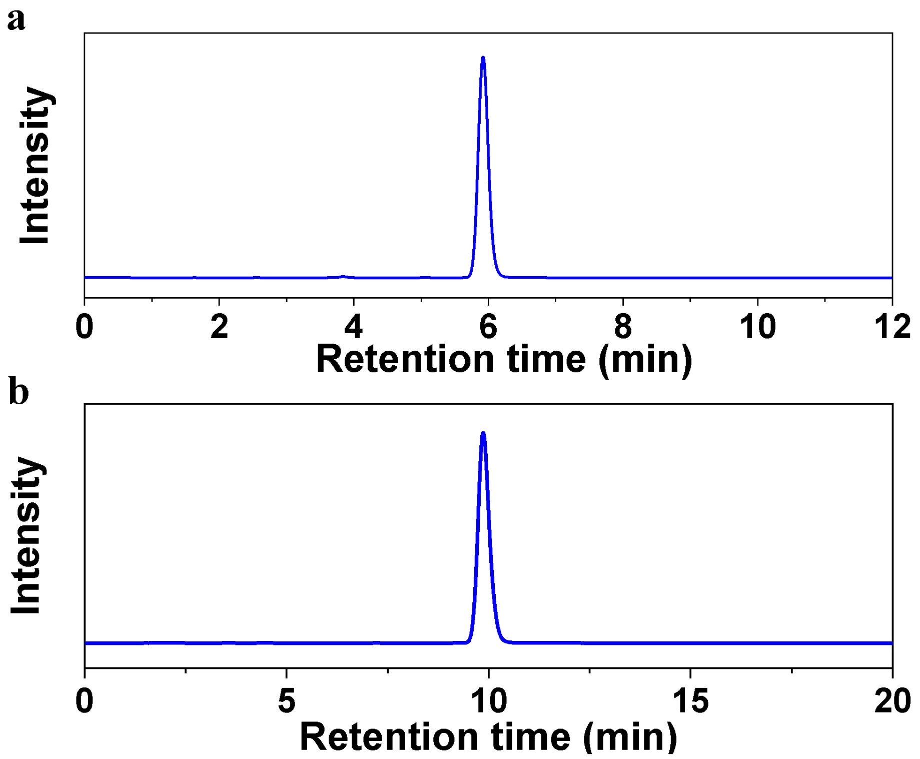

Fig. S138. HPLC curves for a) **NDB** and b) **NPOH** using H_2O/MeCN as mobile phase.

III. References

1. Zeng, W., et al. Achieving nearly 30% external quantum efficiency for orange-red organic light emitting diodes by employing thermally activated delayed fluorescence emitters composed of 1,8-naphthalimide-acridine hybrids. *Advanced Materials* **30**, 1704961 (2018).
2. Wu, Y., et al. Two thermally stable and AIE active 1,8-naphthalimide derivatives with red efficient thermally activated delayed fluorescence. *Dyes and Pigments* **169**, 81-88 (2019).
3. Qi, S., et al. Highly efficient aggregation-induced red-emissive organic thermally activated delayed fluorescence materials with prolonged fluorescence lifetime for time-resolved luminescence bioimaging. *ACS Applied Materials & Interfaces* **12**, 51293-51301 (2020).

4. Li, J. A., et al. Transient and persistent room-temperature mechanoluminescence from a white-light-emitting AIEgen with tricolor emission switching triggered by light. *Angewandte Chemie International Edition* **57**, 6449-6453 (2018).
5. Liu, W., et al. Room-temperature phosphorescence invoked through norbornyl-driven intermolecular interaction intensification with anomalous reversible solid-state photochromism. *Angewandte Chemie International Edition* **59**, 20161-20166 (2020).
